# A catalytic protein–proteomimetic complex: using aromatic oligoamide foldamers as activators of RNase S†
†Electronic supplementary information (ESI) available. See DOI: 10.1039/c9sc00374f


**DOI:** 10.1039/c9sc00374f

**Published:** 2019-02-21

**Authors:** Zsofia Hegedus, Claire M. Grison, Jennifer A. Miles, Silvia Rodriguez-Marin, Stuart L. Warriner, Michael E. Webb, Andrew J. Wilson

**Affiliations:** a School of Chemistry , University of Leeds , Woodhouse Lane , Leeds LS2 9JT , UK . Email: a.j.wilson@leeds.ac.uk; b Astbury Centre For Structural Molecular Biology , University of Leeds , Woodhouse Lane , Leeds LS2 9JT , UK

## Abstract

Foldamers are abiotic molecules that mimic the ability of bio-macromolecules to adopt well-defined and organised secondary, tertiary or quaternary structure. Such templates have enabled the generation of defined architectures which present structurally defined surfaces that can achieve molecular recognition of diverse and complex targets. Far less explored is whether this mimicry of nature can extend to more advanced functions of biological macromolecules such as the generation and activation of catalytic function. In this work, we adopt a novel replacement strategy whereby a segment of protein structure (the S-peptide from RNase S) is replaced by a foldamer that mimics an α-helix. The resultant prosthetic replacement forms a non-covalent complex with the S-protein leading to restoration of catalytic function, despite the absence of a key catalytic residue. Thus this functional protein–proteomimetic complex provides proof that significant segments of protein can be replaced with non-natural building blocks that may, in turn, confer advantageous properties.

## Abstract 2

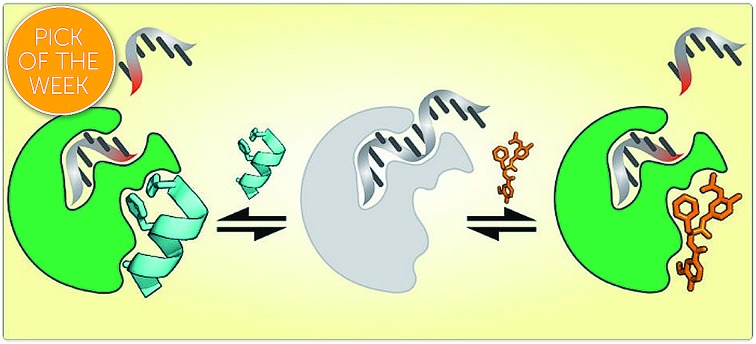
An aromatic oligoamide foldamer acts as an α-helix mimetic and binds to the RNase S-protein resulting in restoration of its catalytic function.

## Introduction

**Fig. 1:**
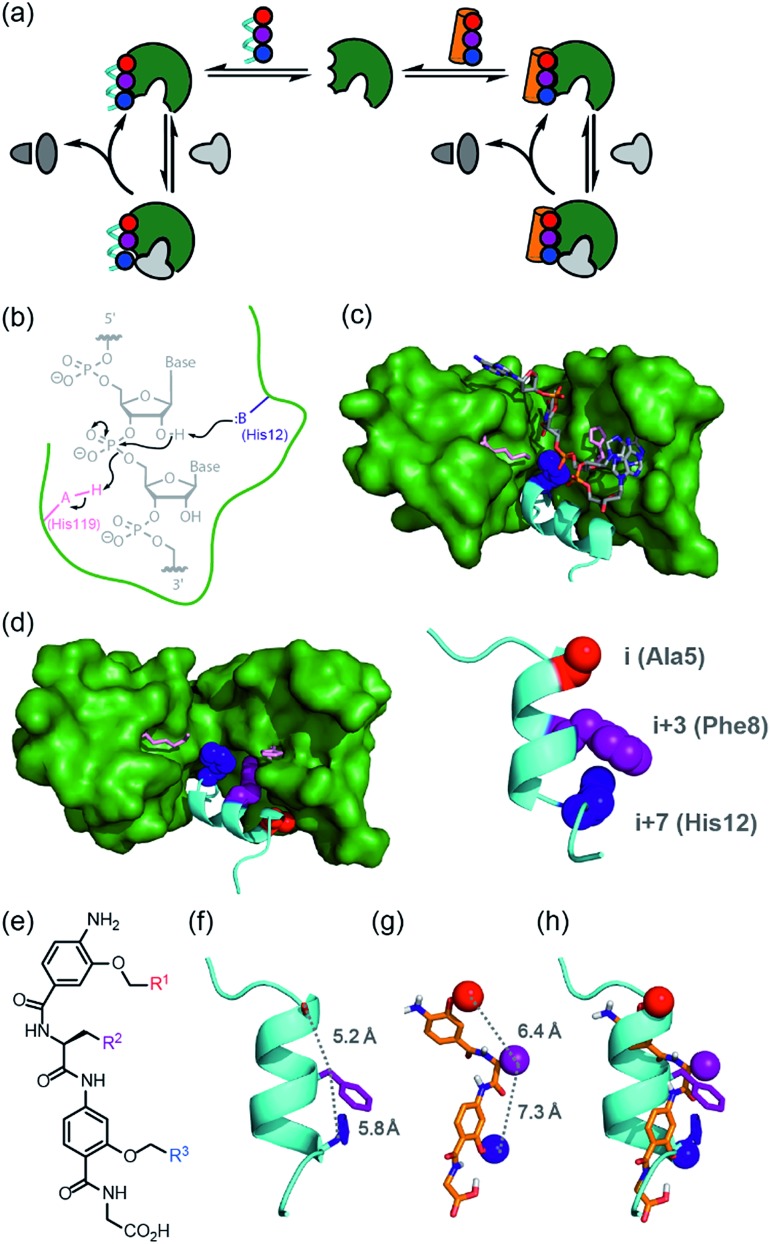
Approach to develop a functional non-covalent protein–proteomimetic complex; (a) schematic depicting strategy to replace a segment of protein structure with a proteomimetic; (b) mechanism for general acid-base catalysed RNA hydrolysis; (c) X-ray crystal structure of bovine ribonuclease A (green) and dApTpApApG (element) complex (PDB ID: 1RCN),^36^ highlighting the key catalytic residues His12 (S-peptide) and Lys41 and His119 (S-protein); (d) X-ray crystal structure of bovine ribonuclease S-protein (green)/S-peptide (cyan) complex (PDB ID: ; 1CJQ),^37^ highlighting key catalytic residues and an *i*, *i* + 3 and *i* + 7 constellation of side chains within the S-peptide sequence (expansion) upon which to design a helix mimetic; (e) chemical structure of an oligoamide foldamer designed to mimic α-helices; (f) S-peptide α-helix illustrating the *i*, *i* + 3 and *i* + 7 side-chains; (g) molecular model of the oligoamide foldamer; (h) overlay of the α-helix and the foldamer highlighting effective side-chain correspondence.

Nature uses a stunning selection of molecular architectures to carry out complex tasks such as catalysis and cell signalling; proteins adopt a specific three-dimensional compact conformation using defined secondary structure elements to spatially position chemical groups so as to perform work. However, our ability to reproduce functions such as catalysis, using synthetic systems remains rudimentary.^1^–^7^ Chemists have readily accepted the challenge of designing and synthesising architectures of comparable complexity to biomacromolecules using non-natural building blocks, specifically through development of foldamers;^8^–^10^ sequences of non-natural monomers that adopt both biomimetic and abiotic secondary, tertiary and quaternary structures that topologically^11^,^12^ and topographically^13^–^15^ mimic native structures. This has allowed creation of foldamers that sterically occlude the binding of natural ligands *e.g.* to inhibit protein–protein interactions,^11^,^13^,^16^–^18^ and which are processed by biomacromolecular machinery.^19^–^21^ however, the control, plasticity and exquisite selectivity of proteins also lies within their dynamic properties, particularly during the catalysis of chemical reactions. We posed the question “Can structural mimics also recapitulate the intrinsic properties required for regulation and catalysis?” If so, a tantalizing alternative to bottom-up design of foldamers would be realizable by replacing components of proteins piece-by-piece^22^,^23^ with non-natural building blocks whilst retaining function.^24^ Such an approach has been termed “protein-prosthesis”^25^ and may even lead to “bionic proteins”.^26^ So far, largely conservative single residue mutations to protein sequence have been made.^27^,^28^ Replacement of entire secondary structural motifs with *topological* mimics (*i.e.* foldamers mimicking local conformation) is less established.^29^–^32^ Replacement of up to three amino acids with peptoid residues in RNAse A afforded a protein-conjugate which, at ten-fold higher concentration than the native protein afforded 10% of the activity. Sequence based replacement of amino acid residues in chorismate mutase afforded proteins with comparable *k*_cat_/*K*_m_ values to the wild type protein (although mutations at the active site further diminished efficiency). Finally, replacement of an α-helix in IL-8 with a designed β-peptide variant resulted in a synthetic protein that retained IL-8 signalling ability. In this work, we study the classic model enzyme complex RNase S, replacing the S-peptide at the *active site* with a small-molecule *topographical* mimic (*i.e.* a foldamer mimicking the shape and surface) of protein secondary structure (Fig. 1a).^33^,^34^ The resulting non-covalent complex recovers the ability to bind and catalyse the cleavage of RNA elicited by the natural RNase S complex despite the unprecedented extent of protein-structure replacement.

## Results

### Selection of RNase S and aromatic oligoamides for construction of bionic proteins

RNase S can be obtained by subtilisin mediated cleavage of RNase A to yield the non-covalently associated S-protein^35^ and short N-terminal S-peptide. In the absence of the S-peptide, the S-protein is catalytically inert but addition of the S-peptide results in restoration of catalytic competence;^38^ this complementation system has thus found use in several applications.^39^–^42^ The S-peptide is bound in an α-helical conformation within a cleft on the S-protein^37^ and bears one of the two histidine residues required for acid-base catalysed hydrolysis of the phosphodiester linkage in RNA (Fig. 1b–d).^42^ The helical nature of this N-terminal segment of the protein thus renders it an ideal motif for replacement with secondary structure mimics. Our group previously introduced a series of proteomimetic scaffolds that mimic the spatial projection of *i*, *i* + (3)4 and *i* + 7 residues on an α-helix (Fig. 1e–h).^14^,^43^,^44^ These scaffolds, accessible *via* solid-phase synthesis,^43^,^45^ can be decorated with side chains that mimic hot-spot residues^46^ on template helices found at protein–protein interfaces to achieve inhibition of protein–protein interactions.^14^,^43^,^44^ We hypothesized that helix mimetics capable of reproducing the spatial orientation and composition of the *i*, *i* + 3 and *i* + 7 side chains found on the S-peptide should bind to the S-protein and were curious if these mimics would also activate the RNA processing function.

### An RNase activity screen identifies activators of RNase S

**Fig. 2:**
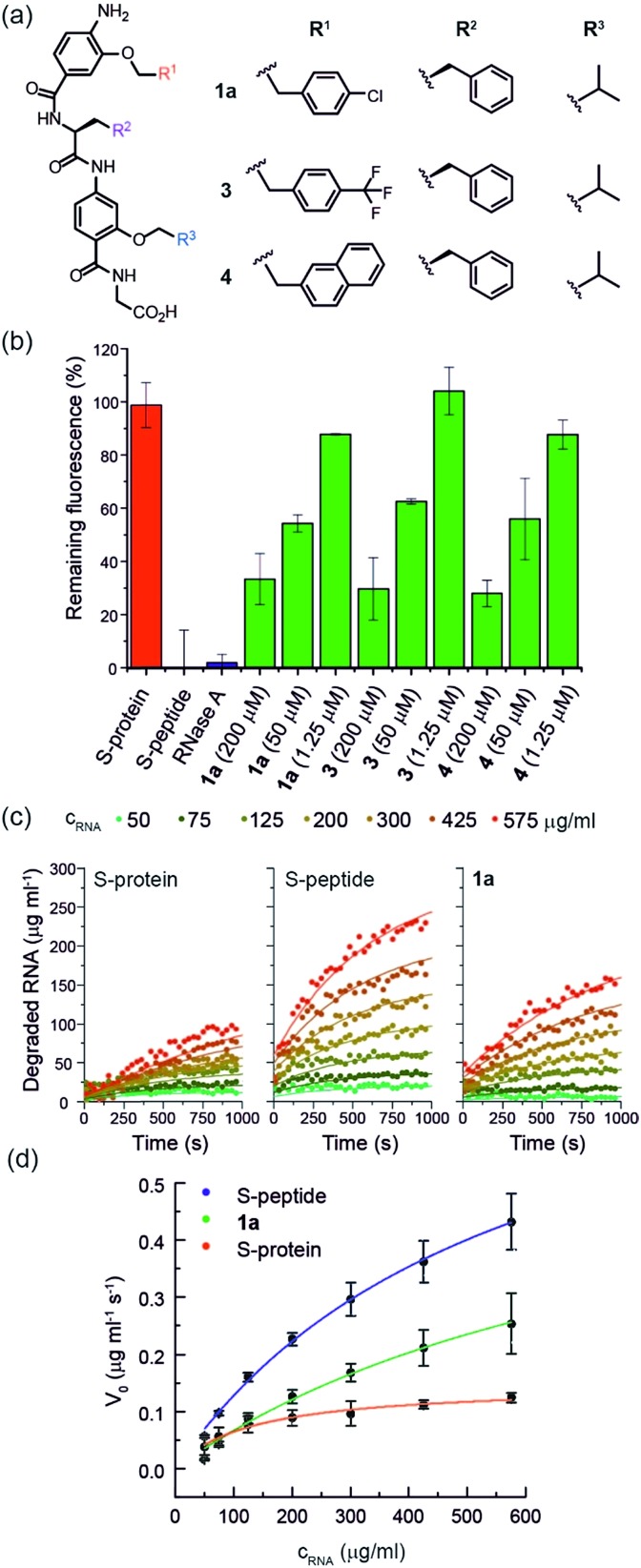
(a) Helix mimetics **1a**, **3**, and **4** as activators of RNase S-protein; (b) RNA degradation at 3 concentrations of selected foldamers (200, 50 and 1.25 μM) shown to be active in the screen (see ESI† for full data set at single concentration and dose response curves) RNA (0.15 mg mL^–1^), S-protein (0.05 μM), S-peptide (0.4 μM), RNase A (0.05 μM) in Tris buffer (50 mM Tris, 100 mM NaCl, pH = 6.5). Fluorescence was measured immediately and every 30 s for 45 minutes of incubation at 25 °C. Remaining fluorescence was calculated using the 45 minute time-point; (c) variation in rate of RNA hydrolysis with substrate concentration for S-protein, S-protein/S-peptide and S-protein/**1a**; S-protein (0.05 μM), S-peptide (0.4 μM), **1a** (100 μM) (for progression curves including RNase A see ESI Fig. S5–S9†); (d) Michaelis–Menten plots for S-protein, S-protein/S-peptide and S-protein/**1a** (see ESI Fig. S10† for Michaelis–Menten plots including RNase A).

Using the S-protein obtained from an established biochemical protocol^35^,^47^ and synthetic S-peptide (obtained *via* solid-phase synthesis), we established an enzymatic assay to assess the ability of reconstituted RNase S to hydrolyse RNA. The assay relies upon binding of ethidium bromide to unhydrolysed RNA.^48^ Using the remaining fluorescence as an approximation of activity, we used the assay to screen libraries of previously prepared aromatic oligoamide helix-mimetics (see ESI for structures; ESI Tables S1 and S2†) derived from *O*-alkylated,^44^,^49^,^50^ *N*-alkylated^14^ and hybrid scaffolds,^43^,^51^ for reactivation of the S-protein (ESI Fig. S1†). Despite the limited prevalence of helix mimetics in our library bearing histidine-mimicking side chains, we were intrigued as to whether binding and global structural emulation would be sufficient to support enzymatic hydrolysis of RNA upon binding to the S-protein. Indeed, a number of the compounds elicited dose-dependent reactivation of the RNase S-protein (Fig. 2a and b and ESI Fig. S2† for representative examples), although the solubility plateau of the mimetics prevented saturation and therefore determination of the maximum concentration at which maximal enzymatic activity is attained. Analysis of the screening hits point to a requirement for a hybrid helix-mimetic scaffold comprising a 3-*O*-alkylated benzamide at the N-terminus with an aromatic R^1^ group, a central phenylalanine in the R^2^ position, a 2-*O*-alkylated benzamide with an R^3^ isopropyl group and a C-terminal glycine (Fig. 2a).

### *h*DM2 can be used to negatively regulate RNase S-protein function through competition for proteomimetics

**Fig. 3:**
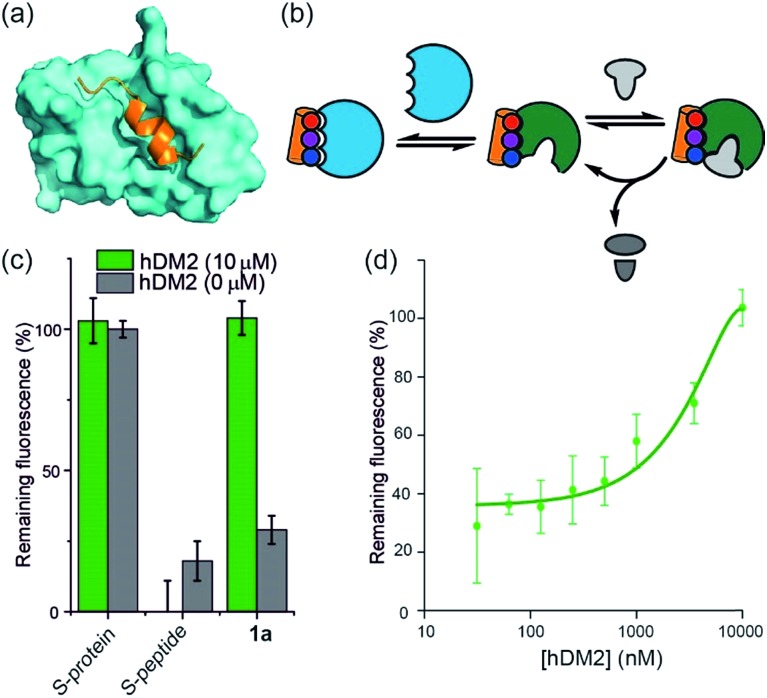
Use of *h*DM2 to create an artificially regulated protein/small molecule enzyme system (a) structure of *h*DM2 (cyan) in complex with p53 (orange) (PDB ID: 1 YCR); (b) schematic depicting strategy to modulate the restoration of S-protein activity in presence of an S-peptide mimetic; (c) comparison of RNA degradation in presence (10 μM) and absence of *h*DM2 for S-protein, S-protein/S-peptide and S-protein/helix mimetic **1a** (100 μM); (d) dose-response of RNA degradation *versus h*DM2 concentration and in presence of S-protein/helix mimetic **1a** (100 μM).

To further establish that a non-covalent interaction between the foldamer and S-protein is responsible for restoration of catalysis, we used a competition experiment with a second protein target of the identified foldamers (Fig. 3). The hybrid helix-mimetic scaffolds had previously been developed as p53/*h*DM2 inhibitors,^43^ thus *h*DM2 was selected for this purpose. Compound **1a** was shown by fluorescence anisotropy competition to act as a low micromolar inhibitor of the p53/*h*DM2 interaction (IC_50_ ∼ 10 μM, ESI Fig. S3 and Table S3† for additional compounds). Addition of excess *h*DM2 suppressed the foldamer-dependent activation of RNA hydrolysis (Fig. 3c and d). Notably, the addition of *h*DM2 to S-protein/S-peptide complex had no effect on catalysis (Fig. 3c) and S-peptide did not compete with fluorescently labelled p53 peptides in an assay against the *h*DM2/p53 interaction (ESI Fig. S4†). It should be noted, RNase S activation appears to be suppressed with sub-stoichiometric *h*DM2 concentrations relative to helix mimetic. Such behaviour could arise due to ternary complex formation between *h*DM2, the helix mimetic **1a** and RNase S, opening up an untapped mechanism of non-covalent RNase S regulation. In addition, the tested oligoamides exhibit a degree of non-specific interaction (see later), which could result in lower effective free mimetic concentration for RNAse S activation when *h*DM2 is present. A more detailed analysis is beyond the scope of the current manuscript and will be the focus of future studies.

### Proteomimetics bind directly to RNase S-protein at the S-peptide binding site

**Fig. 4:**
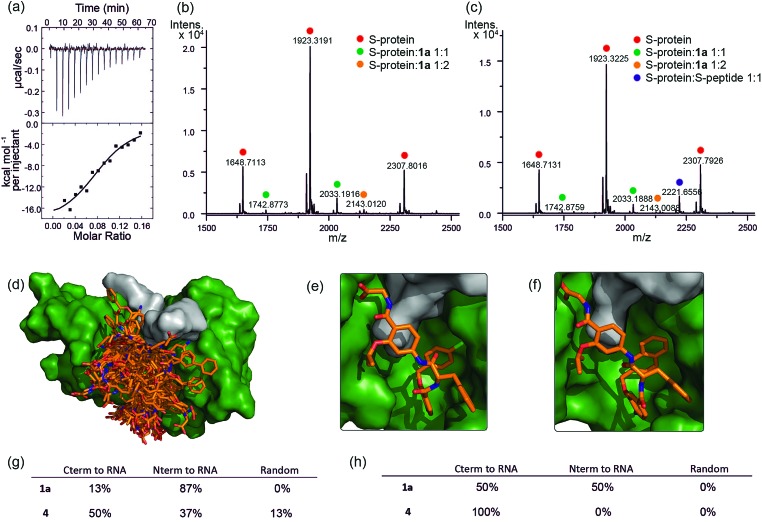
Analyses of binding interaction between helix mimetics and RNase S (a) ITC thermogram of S-protein titrated to compound **1a.** Native MS analysis of 2 μM S-protein in complex with (b) 10 μM **1a** showing S-protein/**1a** complexes having 1 : 1 and 1 : 2 ratio and (c) 10 μM **1a** and 2.5 μM S-peptide showing S-protein/**1a** and S-protein/S-peptide complexes, no ternary complex detected. Modelling studies on helix mimetics as activators of RNase S-protein: (d) overlay of molecular models of 123 conformers of hybrid helix mimetics docked with S-protein (green) using rigid docking. Lowest energy poses of (e) **1a**, and (f) **4** using flexible docking optimized by induced-fit with S-protein; (g) distribution of the different orientations of the docked mimetics (top 20% lowest energy poses) relative to RNA binding site using rigid docking; (h) distribution of the different orientations of the top 20% lowest energy poses of the docked mimetics relative to RNA binding site after induced fit optimization.

To further characterize the RNase S-protein/helix mimetic interaction we performed more detailed analyses focussing on mimetic **1a.** In kinetic analyses, the rate of RNA hydrolysis for the S-protein/**1a** complex increased at higher RNA concentration (Fig. 2c, ESI Fig. S5–S9† and Table S4†) allowing kinetic parameters to be assessed (Fig. 2d and ESI Fig. S10†). *k*_cat_ values could not be extracted from the kinetic data due to the unknown molar concentration of RNA cleavage sites. In order to numerically compare the enzymatic efficiency we therefore fitted *V*_max_/*K*_M_ values using the first 3 data points of the Michaelis–Menten plots. Comparing at constant S-protein concentration (0.05 μM), the data for S-protein/**1a** (*V*_max_/*K*_M_ = 9.52 ± 0.48 × 10^–4^ s^–1^) indicate that *V*_max_/*K*_M_ (the first order rate constant at low substrate concentration) decreases in comparison to S-protein/S-peptide (*V*_max_/*K*_M_ = 1.36 ± 0.07 × 10^–3^ s^–1^), but increases in comparison to the S-protein alone (*V*_max_/*K*_M_ = 5.17 ± 0.84 × 10^–4^ s^–1^), indicating that **1a** effectively activates the S-protein. ITC analysis provided further evidence for a direct interaction between the proteomimetic **1a** and S-protein (Fig. 4a). This experiment was performed by titration of S-protein into mimetic; binding was observed to occur with low micromolar affinity (*K*_D_ = 1.85 ± 0.97 μM, *n* = 0.1, Table S5†).

The affinity is only one order of magnitude weaker than the interaction of the native S-peptide with S-protein (*K*_D_ = 0.13 ± 0.02 μM, ESI Fig. S11†), whilst the non-stoichiometric interaction may arise in part due to some non-specific binding which we discuss further below. Further evidence of an interaction between the S-protein and helix mimetic **1a** was obtained *via* native ESI-MS. Using a 5-fold excess of the mimetic relative to the S-protein, a 1 : 1 complex and a lower intensity 1 : 2 S-protein/mimetic **1a** complex could be detected (Fig. 4b). This minor species may again imply a degree of nonspecific binding to S-protein and suggests that the observed apparent *K*_D_ from ITC may be an average of the simultaneously occurring binding events. Further native MS competition-binding experiments were performed to probe the binding site for the mimetic by keeping the concentrations of S-protein and **1a** constant while using increasing concentrations of S-peptide; no ternary complex could be detected (Fig. 4c), suggesting the S-peptide and mimetic **1a** compete for the same binding site on S-protein.

To provide additional insight into the mode of recognition between the mimetics and S-protein a range of docking analyses were performed (see ESI† for full details of the analyses). Helix mimetics were energy-minimized and subjected to *Monte Carlo* conformational analysis and then lowest energy conformers retained for docking experiments. These lowest energy conformers were docked into the rigid-body structures of either (i) the S-protein or (ii) RNase A (lacking the S-peptide residues) in complex with a tetranucleotide. All input ligands gave a pose in the S-protein binding groove in the region occupied by His12 of the S-peptide (*e.g.*Fig. 4d). For docking to the S-protein alone, ligands were observed in an extended form in both the S-peptide and the RNA-binding pocket while docking against the nucleotide-RNase A complex (lacking the S-peptide residues) led to binding in the S-peptide pocket but without a well-defined orientation (ESI Fig. S12†). In contrast, flexible docking followed by induced fit optimization gave a more consistent orientation with the majority of the lowest energy poses aligned with the C-terminus of the aromatic oligoamide close to the RNA (Fig. 4e and f for representative examples **1a** and **4** and ESI Fig. S13† for the results without induced fit-optimization). This suggests that the C-terminal carboxylate might fulfil the role of an ionisable group in mediating acid-base catalysed hydrolysis of RNA. To test this hypothesis, we prepared derivatives of **1a** bearing an N-terminal acetanilide **1b**, a C-terminal carboxamide **1c** and with both modifications **1d** (see ESI†). Unfortunately, the solubility of these derivatives was such that inconsistent and therefore inconclusive behaviour was observed in the kinetic studies although docking afforded similar results to those observed with the parent hybrid mimetics, *i.e.* with the C-terminus of the mimetic oriented close to the RNA (ESI Fig. S14†). Finally, the docking analyses highlight some imperfections in the original design: generally, the aromatic side-chains of the mimetics occupy the designed binding sites in the majority of the docked ligands, however, we observed diverse conformations and multiple different orientations of the ligand due to the increased conformational flexibility originating from the central α-amino acid. This points towards an induced fit mechanism of binding, where the aromatic side-chain acts as anchor residue (see ESI Fig. S15,† for an overlay of the S-peptide with docked poses obtained by flexible docking).

With the goal of improving catalytic activity we also: (i) attempted to prepare helix mimetics bearing imidazole groups on the *O*-alkyl side chains, and (ii) helix mimetics bearing d- or l-histidine instead of glycine at the C-terminus; our synthesis was not compatible with the former approach due to facile elimination of the imidazole side chain during synthesis whilst the later compounds did not reactivate S-protein. It is noteworthy however that removal of the catalytic histidine residues need not abrogate catalysis; RNase A variants with either His12Ala and His119Ala changes to primary sequence were previously shown to retain some catalytic activity.^52^ Loss of the His12 imidazole was shown to decrease the affinity for the transition state by 10^4^ fold for a range of substrates (Poly(C), UpA and UpOC_6_H_4_-*p*-NO_2_ hydrolysis), whereas eliminating the imidazole in His119 had a comparable effect on Poly(C) and UpA hydrolysis, but not the cleavage of UpOC_6_H_4_-*p*-NO_2_ indicating the role of general acid for His119 to protonate the leaving group.^52^ Assuming a water fulfils the role of general base *in lieu* of His12, the rate of RNA hydrolysis would be anticipated to be reduced significantly – such behavior is thus consistent with our observations *i.e.* it may be the case that **1a** binds to the S-protein so as to form a structurally competent site for catalysis whilst the role of general base is fulfilled by water.

**Fig. 5:**
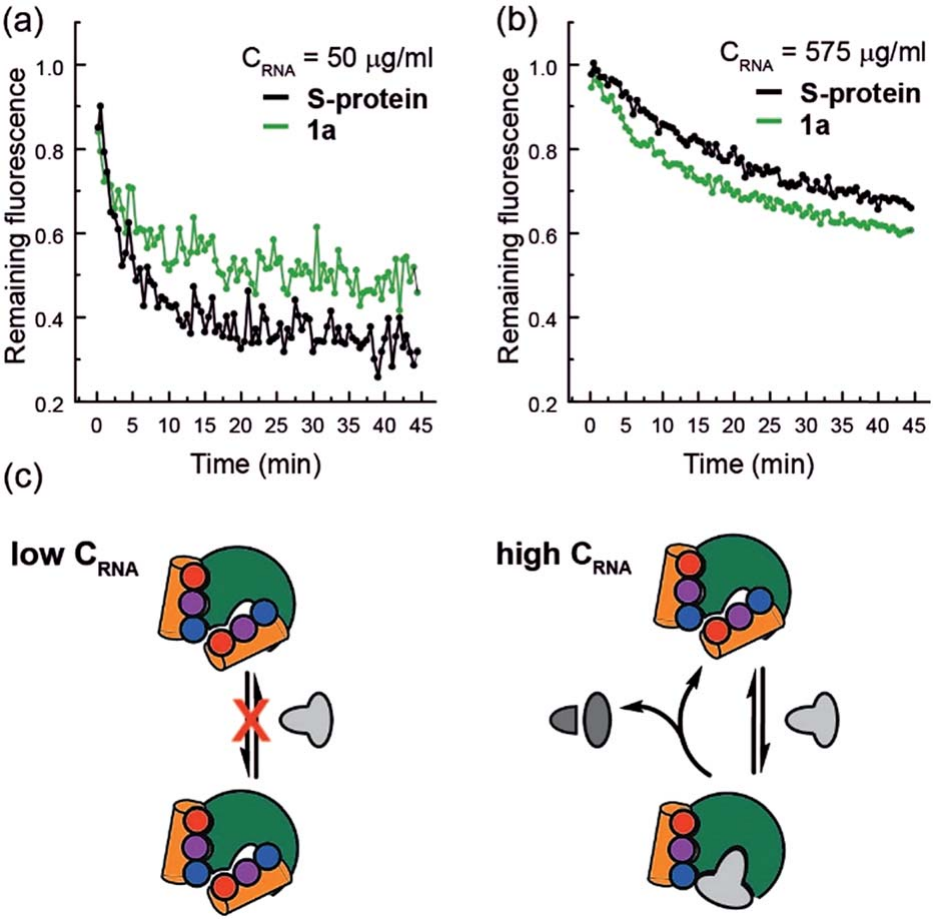
RNA hydrolysis assessed by remaining fluorescence of the EtBr labelled RNA at (a) 50 μg mL^–1^ RNA concentration and (b) 575 μg mL^–1^ RNA concentration; (c) schematic depicting the hypothesized mechanism of enzyme inhibition at low RNA (left) and activation at high RNA concentrations (right).

Finally, closer analysis of the kinetic data allow formulation of a plausible hypothesis that can rationalize the ITC and ESI-MS binding data. At low concentrations of RNA, it appears that the mimetic acts as an inhibitor of RNA hydrolysis (Fig. 5a), whereas at higher RNA concentrations (Fig. 5b), presence of the mimetic accelerates hydrolysis. These data imply that the mimetic can bind to both the S-protein-binding site, and competitively with the RNA binding site (Fig. 5c), the observation that helix mimetics can interact with the RNA binding site in the docking analyses performed in the absence of RNA (see ESI Fig. S12†), support this hypothesis.

## Conclusion

In this work we have demonstrated that aromatic oligoamide foldamers can act as a component of a functional quaternary structure that performs RNA hydrolysis. Screening a panel of helix mimetics in an RNase assay led to identification of compounds that upon binding S-protein led to partial recovery of enzymatic function observed for the S-protein/S-peptide complex. The proteomimetics were shown to display first-order kinetics with enzymatic efficiency dependent on both concentration of proteomimetic, and RNA, and, directly bind to S-protein with low μM affinity. As further evidence of the potential to generate new capabilities using this approach, we demonstrate that the S-protein/proteomimetic enzyme function can be readily regulated using an additional protein – *h*DM2 – which competitively binds to the proteomimetics, a property not observed for the S-protein/S-peptide complex. Non-covalent MS analyses suggest the mimetics bind to the S-peptide binding site on the S-protein, a conclusion supported by molecular modelling. Crucially none of the proteomimetics bear an imidazole side chain to facilitate catalysis; consequently, the efficiency of RNA hydrolysis is >10^4^-fold lower than for fully functional RNase S-protein/S-peptide complex. Such an observation is consistent with prior studies on RNase A which established that His12 (within the S-peptide sequence) could be removed without complete loss of catalytic competence.^52^ Significantly, our experimental and *in silico* analyses, point to opportunities to address imperfections in the design through future studies; the solubility threshold, and lower selectivity of the mimetics (exploited advantageously here in the *h*DM2 competition experiment), together with improved potency should be addressed to achieve superior designs. We note that aromatic oligoamides of this nature have shown a measure of selectivity previously^14^,^43^,^44^ and that solubility and selectivity can be improved using a number of strategies including the use of peptide-foldamer hybrids.^26^,^53^,^54^ Incorporation of further functionality that allows mimicry of more than the *i*, *i* + 3 and *i* + 7 constellation on an α-helix should further aid these objectives.

A significant goal in biomimetic chemistry is to reproduce structure and functionality of biomacromolecules using synthetic molecules. Foldamers do so firstly by mimicking the conformations adopted by bio-macromolecules and then their functions *e.g.* molecular recognition,^15^ control over reactivity,^55^ transduction of information.^56^ Catalytic foldamers^57^ have proven considerably more elusive; aldolase foldamers have been described which are more efficient than designed aldolases,^58^ but do not reach the activities of natural enzymes, whilst the addition of catalytic groups to foldamers results in stereoselective oxidations.^59^ In asking the question: “Can the astonishing 3D structures and functions of proteins be attained with building blocks other than α-amino acids?” we have taken a different approach in replacing parts of a functional protein; applied to an extremely efficient enzyme such as RNase S as we have done here places a high bar for success. Hence, although moderately active, the “bionic” S-protein/proteomimetic complexes described in this work provide for the first time, proof of concept that significant tranches of protein can be replaced with *topographical* mimics of protein structure, which may confer advantageous properties *e.g.* thermal stability, resistance to proteolysis, ability to function in organic solvents; a horizon we will explore in future work.

## Conflicts of interest

There are no conflicts to declare.

## Supplementary Material

Supplementary information
