# Supplementary material for: A catalytic protein–proteomimetic complex: using aromatic oligoamide foldamers as activators of RNase S
Source: Chem Sci. 2019 Feb 21;10(14):3956–62. doi: 10.1039/c9sc00374f (PMC6461108; doi:10.1039/c9sc00374f)
Supplement: Supplementary file 1 [file SC-010-C9SC00374F-s001.pdf]

Electronic Supplementary Material (ESI) for Chemical Science.
This journal is © The Royal Society of Chemistry 2019

A Catalytic Protein-Proteomimetic Complex: Using Aromatic Oligoamide Foldamers as Activators of RNase S

Supporting Information

Claire M. Grison,[a],[b] Zsofia Hegedus, [a],[b] Jennifer A. Miles,[a],[b], Silvia Rodriguez-Marin,[a],[b] Stuart L. Warriner,[a],[b] Michael E. Webb[a],[b] and Andrew J. Wilson,*[a],[b]

[a] School of Chemistry, University of Leeds, Woodhouse Lane, Leeds LS2 9JT, UK.
[b] Astbury Centre For Structural Molecular Biology, University of Leeds, Woodhouse Lane, Leeds LS2 9JT, UK
E-mail: A.J.Wilson@leeds.ac.uk

Supplementary figure and tables ....
Library of mimetics tested **2**
RNA degradation assays **10**
Regulation of S-protein activity **11**
Docking experiments **16**

Supplementary Methods ....
Preparation of S protein **18**
General procedure for RNA degradation assays **18**
Library of mimetics 19
Dose-response of best mimetics 19
Kinetic data 19
Dose-response for SAR activity 20
Regulation of S-protein activity 20
Isothermal titration calorimetry **21**
Native MS **21**
Docking experiments **21**
Protein preparation 21
Grid generation 22
Ligand preparation: Molecular Mechanics calculation 22
Docking and score **23**
Fluorescence anisotropy **23**
Synthesis of mimetics **24**
General Comments 24
General procedure for the synthesis of S peptide 25
LCMS characterization of S-peptide **26**
General procedure for the synthesis of mimetics **27**
Numbering system for proteomimetic scaffolds **29**
LCMS characterization of mimetics 1a-d **30**

Syntheses and Characterization for Previously unpublished Helix Mimetics ....

Supplementary figure and tables

Library of mimetics tested

Supplementary Table S1. General Structures of the tested mimetics

Hybrid mimetics	O-alkylated mimetics	N-alkylated mimetics
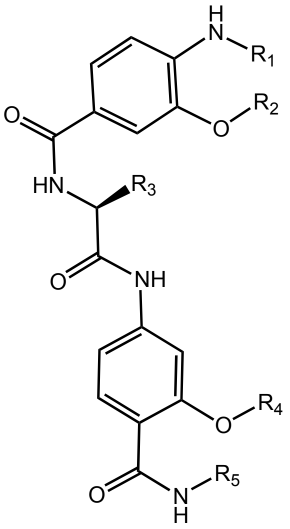	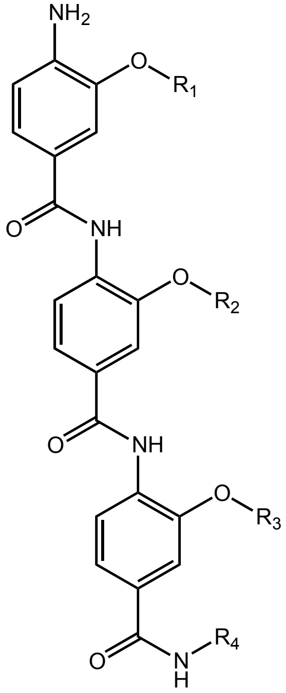	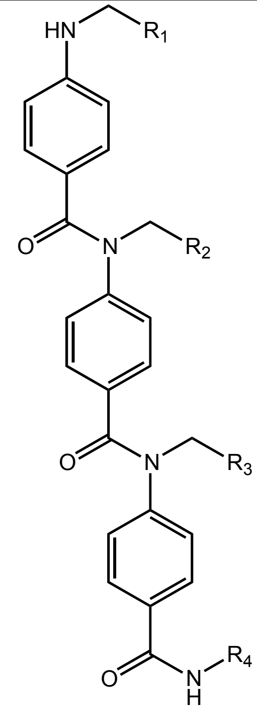

Methods for synthesis of helix mimetics are described later, compounds **19-26**, **28-32**, **46**, **51-54**, all[1]; **27**, **33-41**, **47-48**, **50** all[2]; **49**, **42-45**, all[3]; **59**,[4] **60**,[5] **61-64**,[6] **65-68**, **70-76**, **78-85**, all[7]; **69**, **77** both[8] were all described previously. The remaining compounds are novel and their characterization is included later.

Supplementary Table S2. Sequences of the tested mimetics

Hybrid mimetics					
Compound No.	N-ter (R1)	Arylamide (R2)	Amino acid (R3)	Arylamide (R4)	C-ter (R5)
**1 (=1a)**	H	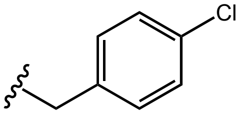	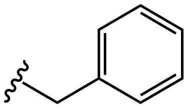	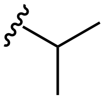	Gly
**2**	H	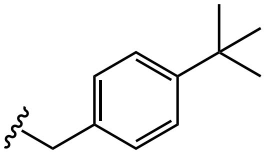	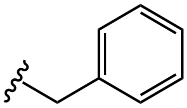	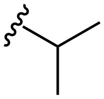	Gly
**3**	H	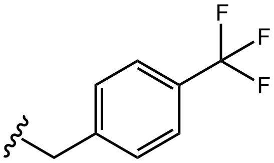	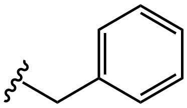	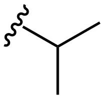	Gly
**4**	H	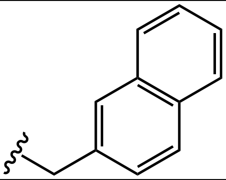	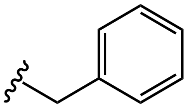	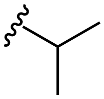	Gly
**5**	H	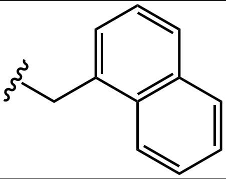	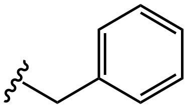	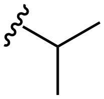	Gly
**6**	H	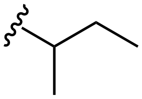	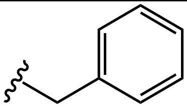	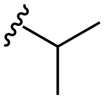	Gly
**7**	H	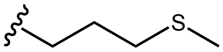	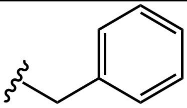	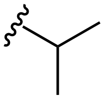	Gly
**8**	H	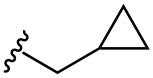	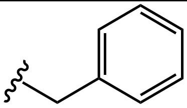	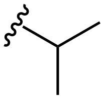	Gly
**9**	H	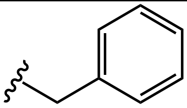	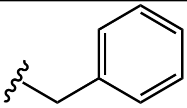	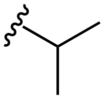	Lys
**10**	H	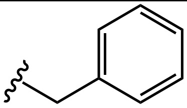	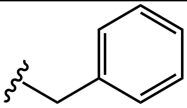	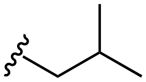	Gly
**11**	H	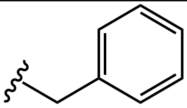	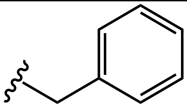	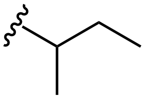	Gly
**12**	H	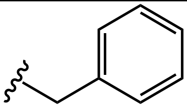	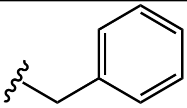	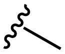	Gly

**13**	H	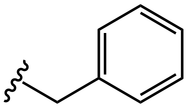	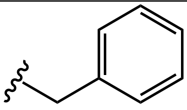 (N-Me)	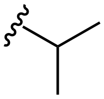	Gly
**14**	H	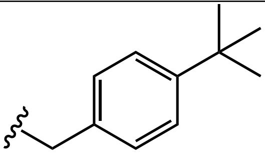	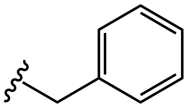 (R)	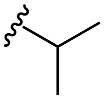	Gly
**15**	Amino-propyl	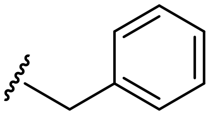	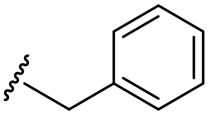	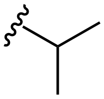	Gly
**16**	H	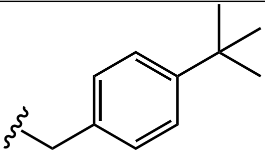	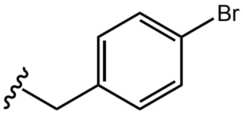	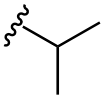	Gly
**17**	H	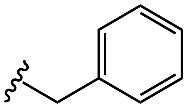	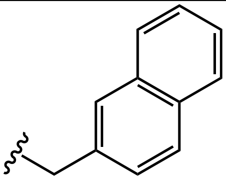 (S)	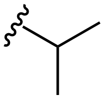	Gly
**18**	H	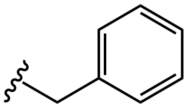	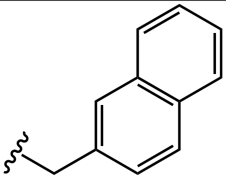 (R)	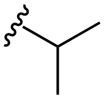	Gly
**19**	H	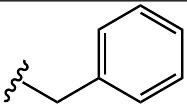	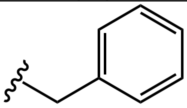	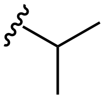	Gly
**20**	–Bn	/	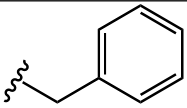	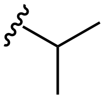	Gly
**21**	H	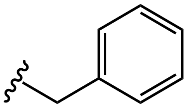	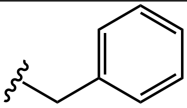 (R)	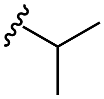	Gly
**22**	H	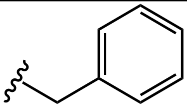 (2-O)	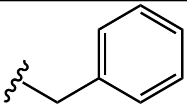	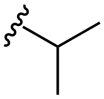	Gly
**23**	H	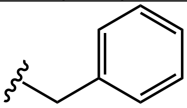	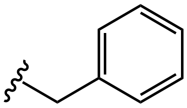	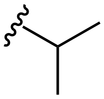 (3-O)	Gly
**24**	–Bn	/	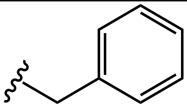	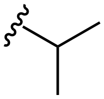	Gly

				(3-O)	
**25**	H	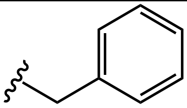 (2-O)	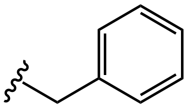	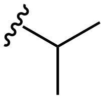 (3-O)	Gly
**26**	H	/	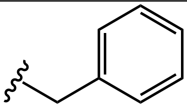	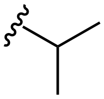	Gly
**27**	H	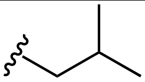 (2-O)	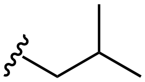	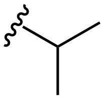	Gly
**28**	H	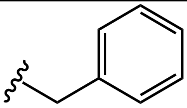	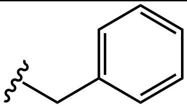	/	Gly
**29**	–Bn	/	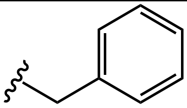	/	Gly
**30**	H	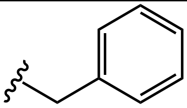 (2-O)	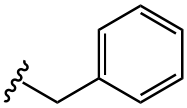	/	Gly
**31**	H	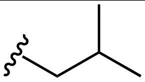 (2-O)	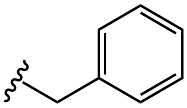	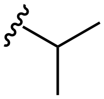	Gly
**32**	H	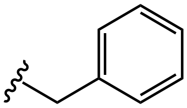	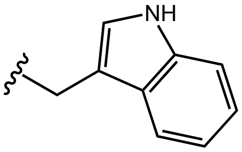	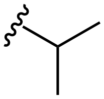	Gly
**33**	H	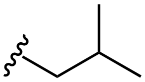 (2-O)	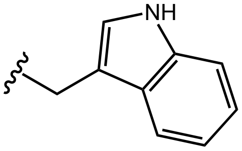	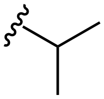	Gly
**34**	H	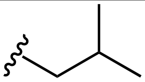 (2-O)	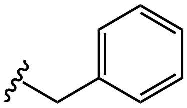	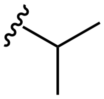	Glu
**35**	H	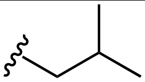 (2-O)	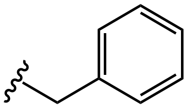	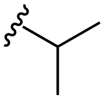	Pro
**36**	Glu	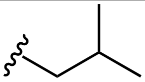 (2-O)	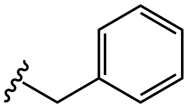	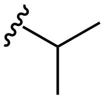	Gly
**37**	H	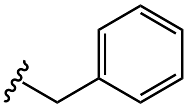	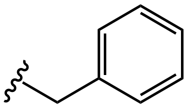	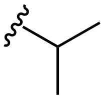	[1-biotin-3-triazol-propyl-

					Gly
**38**	Arg	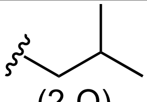 (2-O)	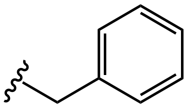	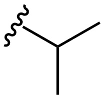	Gly
**39**	Glu	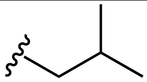 (2-O)	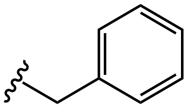	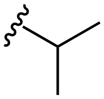	Pro
**40**	Arg	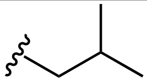 (2-O)	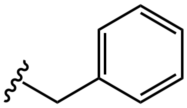	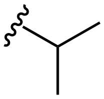	Pro
**41**	Arg	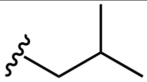 (2-O)	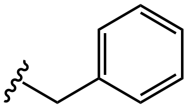	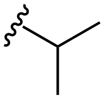	Asp
**42**	H	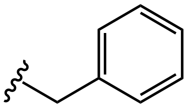	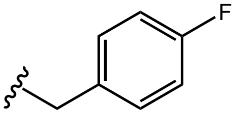	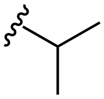	Gly
**43**	H	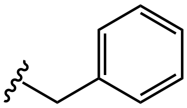	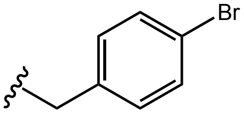	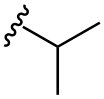	Gly
**44**	H	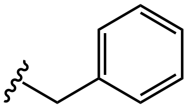	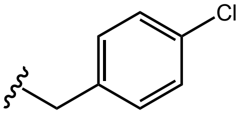	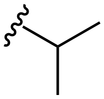	Gly
**45**	H	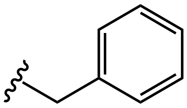	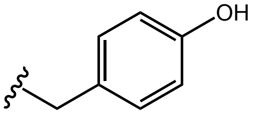	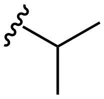	Gly
**46**	H	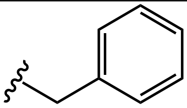	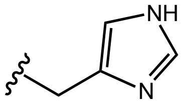	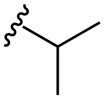	Gly
**47**	H	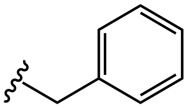 (2-O)	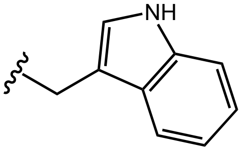	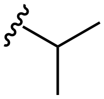	Gly
**48**	H	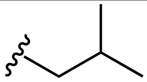 (2-O)	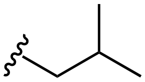	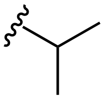	Leu
**49**	H	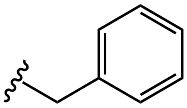	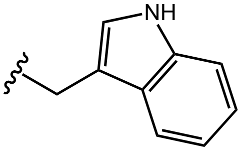 (R)	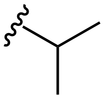	Gly
**50**	H	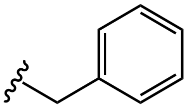 (2-O)	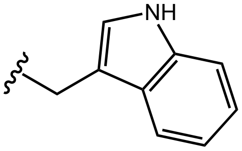 (R)	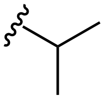	Gly

**51**	Ac–	/	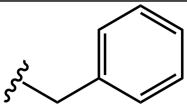	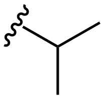	Gly
**52**	H	/	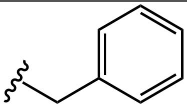	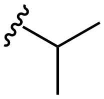	Gly
**53**	H	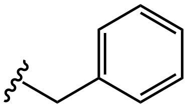	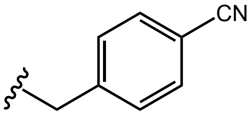	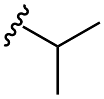	Gly
**54**	H	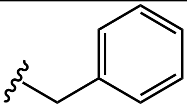	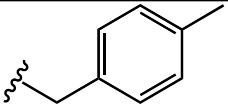	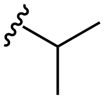	Gly

**O-alkylated mimetics**				
Compound No.	Arylamide (R1)	Arylamide (R2)	Arylamide (R3)	C-ter (R4)
**55**	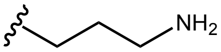	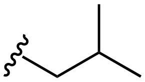	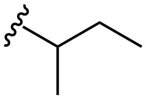	Gly
**56**	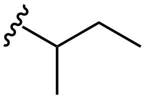	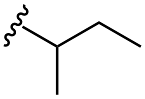	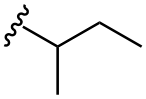	Gly
**57**	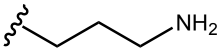	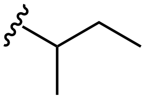	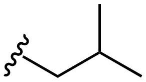	Gly
**58**	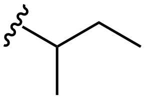	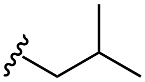	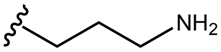	Gly
**59**	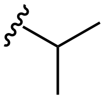	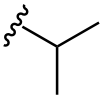	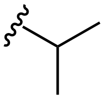	/
**60**	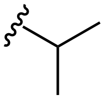	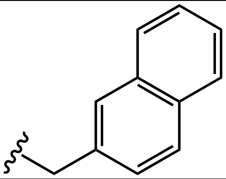	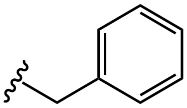	/
**61**	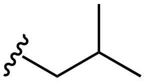	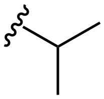	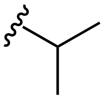	/
**62**	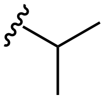	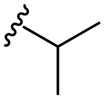	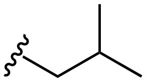	/
**63**	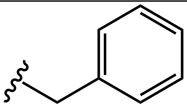	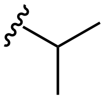	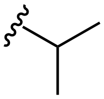	/

**64**	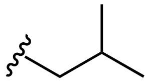	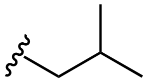	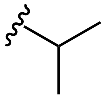	/

**N-alkylated mimetcs**				
Compound No.	Arylamide (R1)	Arylamide (R2)	Arylamide (R3)	C-ter (R4)
**65**	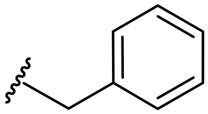	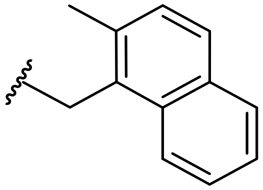	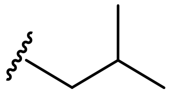	Gly
**66**	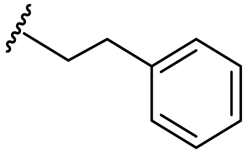	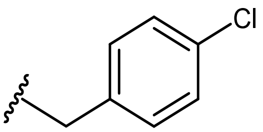	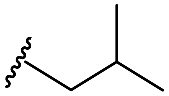	Gly
**67**	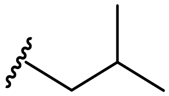	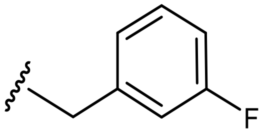	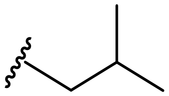	Gly
**68**	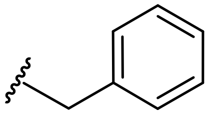	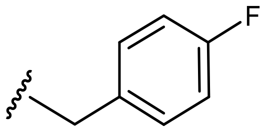	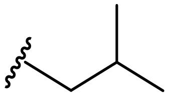	Gly
**69**	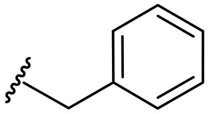	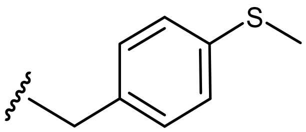	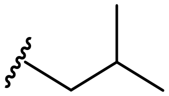	Gly
**70**	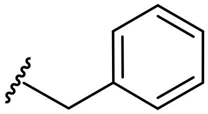	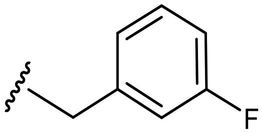	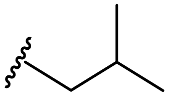	Gly
**71**	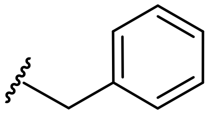	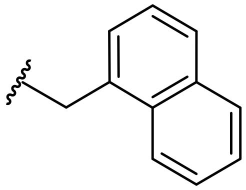	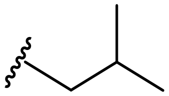	Gly
**72**	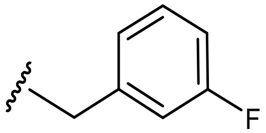	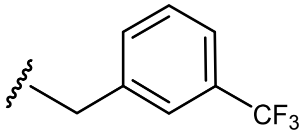	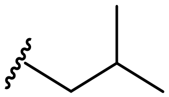	Gly
**73**	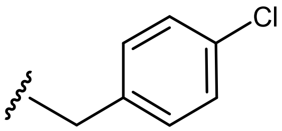	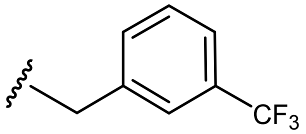	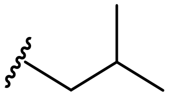	Gly

**74**	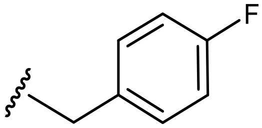	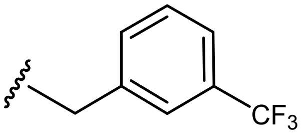	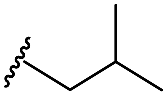	Gly
**75**	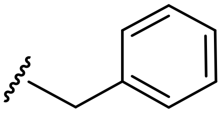	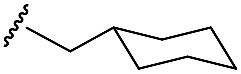	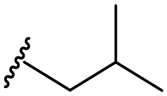	Gly
**76**	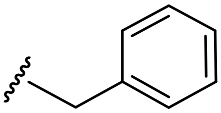	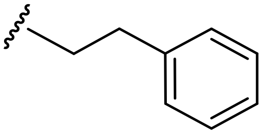	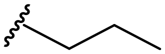	Gly
**77**	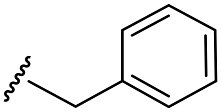	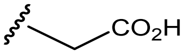	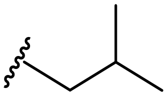	Gly
**78**	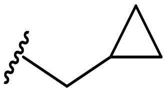	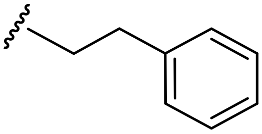	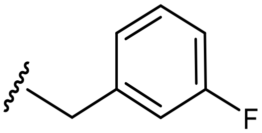	Gly
**79**	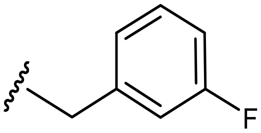	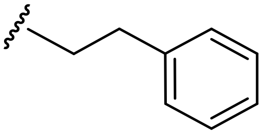	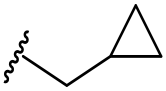	Gly
**80**	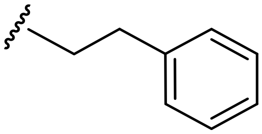	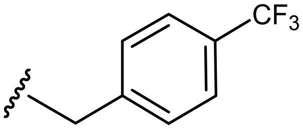	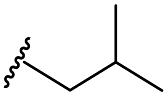	Gly
**81**	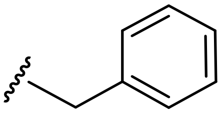	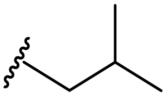	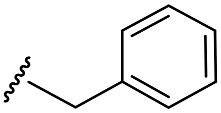	Gly
**82**	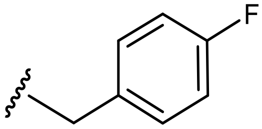	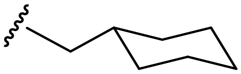	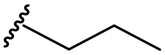	Gly
**83**	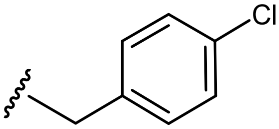	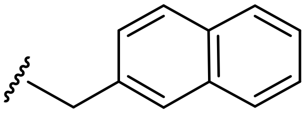	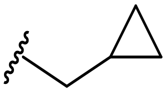	Gly
**84**	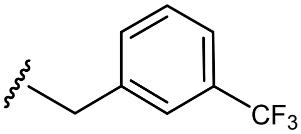	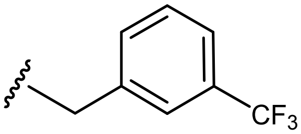	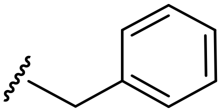	Gly
**85**	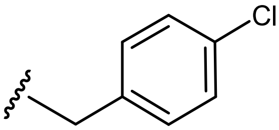	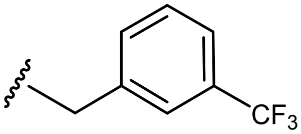	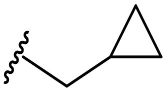	Gly

RNA degradation assays

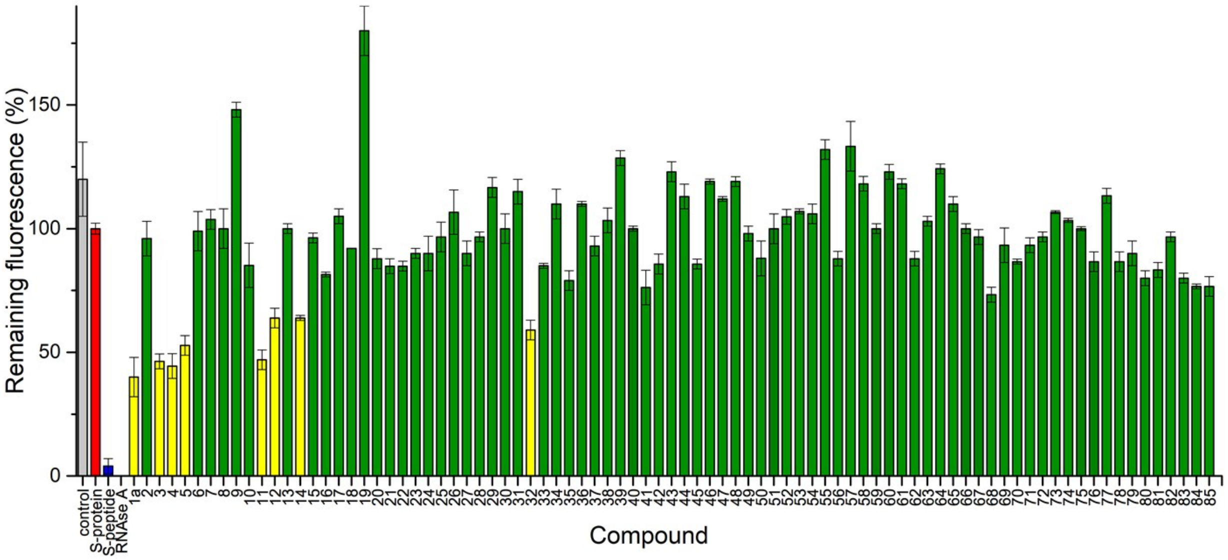

Supplementary Figure S1: Test of a library of S-peptide mimetics for restoring S-protein activity using RNA degradation assay, monitoring the decrease of EtBr fluorescence upon RNA degradation. A library of 85 alpha-helix mimetics (Hybrid, O-alkylated and N-alkylated mimetics) was tested at a single concentration of 100 μM in 50 mM of Tris, 100 mM of NaCl, pH = 6.5 using 0.05 μM S-protein, along with two negative control experiments: RNA only (grey), S-protein only (0.05 μM, red) and two positive controls: S-peptide (0.4 μM, light blue) and RNaseA (0.05 μM, dark blue). The most active mimetics (remaining fluorescence below 60%) are highlighted yellow.

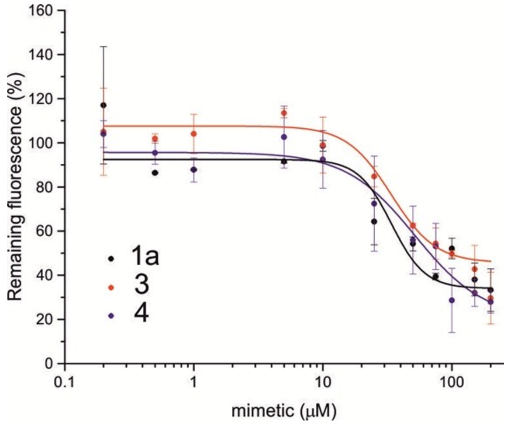

Supplementary Figure S2: Dose-response curves using RNA degradation assay of three highest activity mimetics **1a**, **3** and **4**.

Regulation of S-protein activity

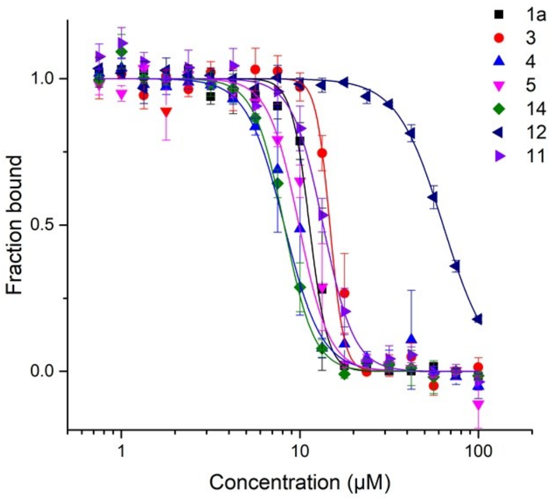

Supplementary Figure S3: Fluorescence anisotropy competition assay of mimetics **1a, 3, 4, 5, 11, 12** and **14** against the p53/hDM2 interaction in 50 mM phosphate buffer at pH 7.5, containing 200 mM NaCl and 0.02 $mg.mL^{-1}$ bovine serum albumin using FITC-$p53_{15\text{-}31}$ as tracer.

Supplementary Table S3. Comparison of the fitted IC_{50} values of selected mimetics derived from fluorescence anisotropy competition experiments against the p53/hDM2 interaction with their activity in the RNA degradation assay at a single 100 μM concentration.

Compounds	IC_{50} (μM)	% remaining fluorescence (100 μM)
**1a**	10.9 ± 0.6	40
**3**	14.6 ± 0.7	46
**4**	8.1 ± 0.3	44
**5**	9.5 ± 0.5	53
**12**	68.2 ± 6.1	64
**11**	13.4 ± 0.7	64
**14**	8.3 ± 0.3	47

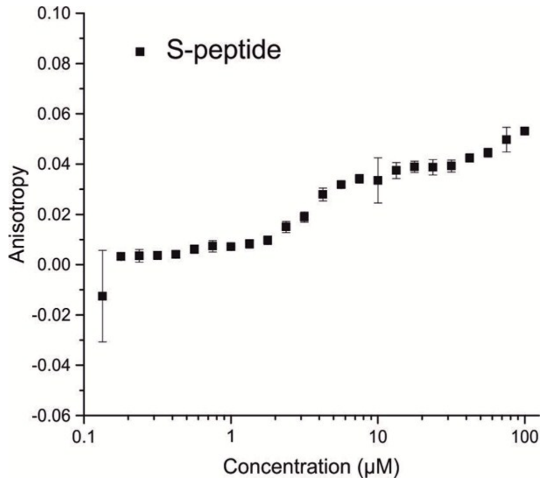

Supplementary Figure S4: Fluorescence anisotropy competition assay of S-peptide against the p53/hDM2 interaction in 50 mM phosphate buffer at pH 7.5, containing 200 mM NaCl and 0.02 mg.mL^{-1} bovine serum albumin using FITC-p53$_{15-31}$ as tracer.

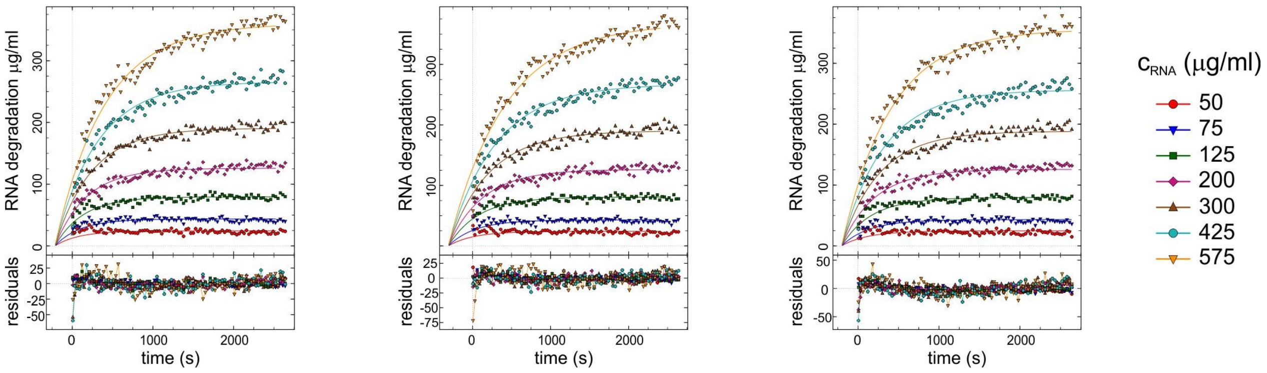

Supplementary Figure S5: Fitted progression curves for RNaseA (0.05 μM RNase A)

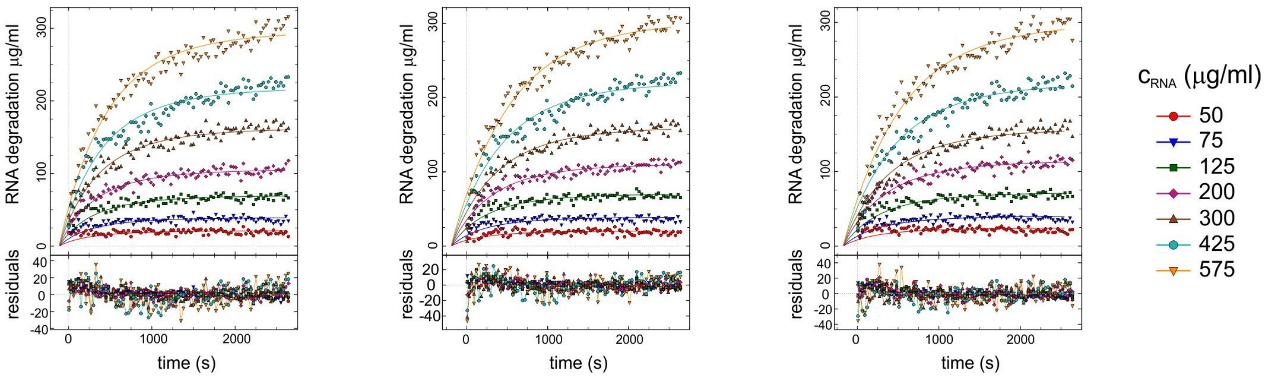

Supplementary Figure S6: Fitted progression curves for S-protein/S-peptide (0.05 μM S-protein, 0.4 μM S-peptide)

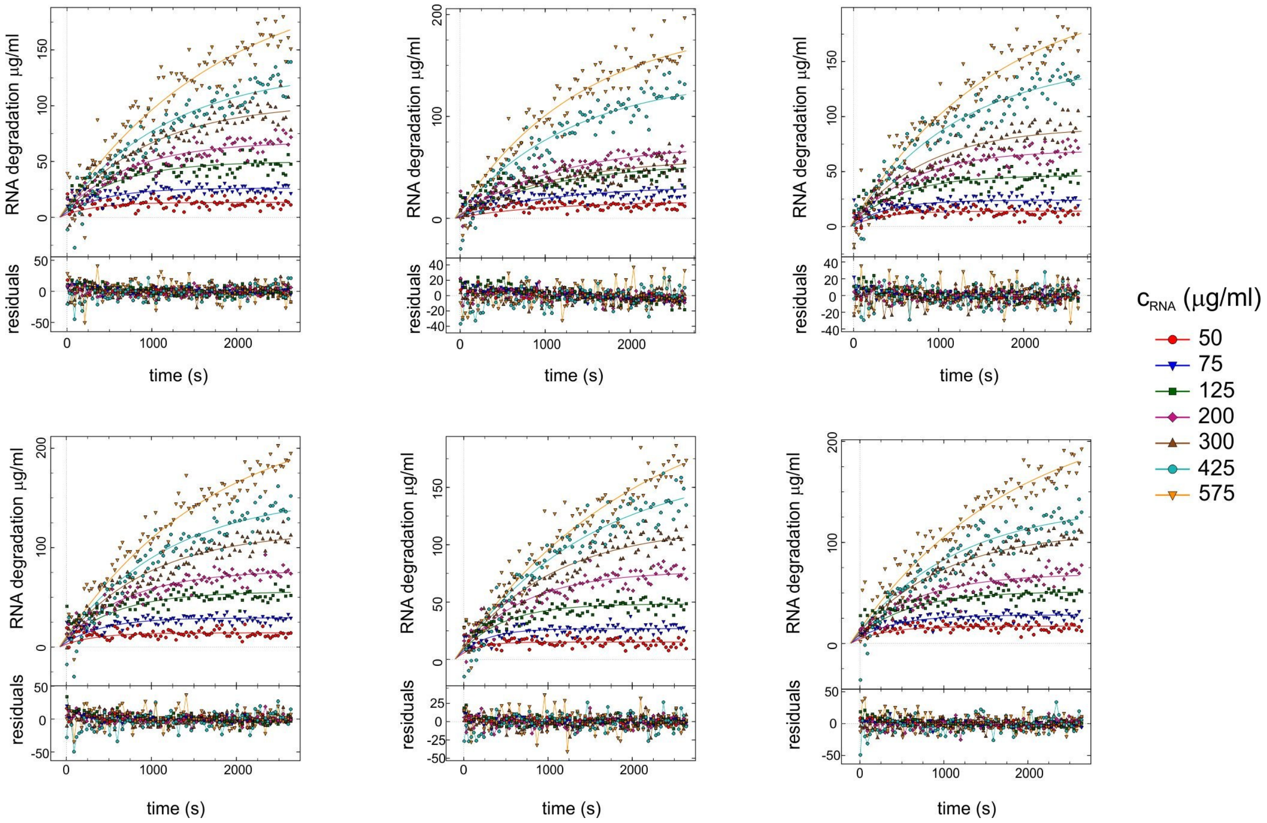

Supplementary Figure S7: Fitted progression curves for S-protein (0.05 μM S-protein)

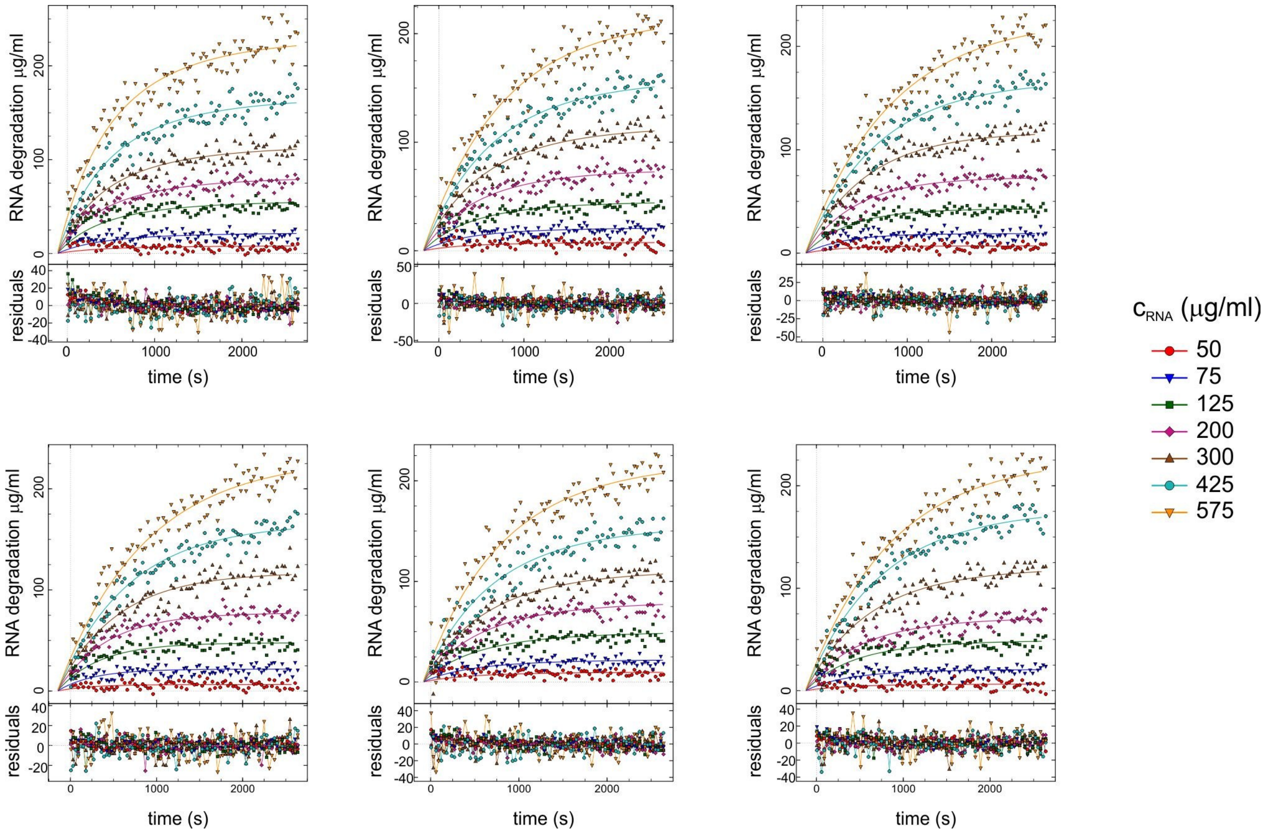

Supplementary Figure S8: Fitted progression curves for S-protein/**1a** (0.05 µM S-protein, 100 µM **1a**)

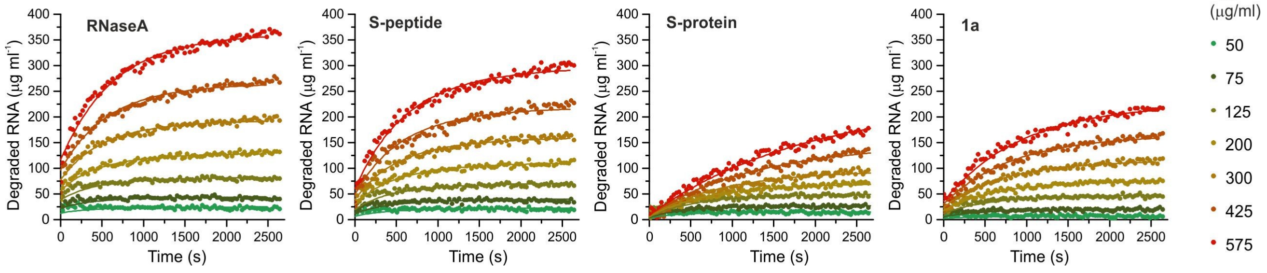

Supplementary Figure S9: Average of the fitted progression curves for RNA degradation at a fixed concentration of protein (0.05 µM RNase A or S-protein), S-peptide (0.4 µM) and **1a** (100 µM)

Table S4. Average initial rates for RNA degradation at a fixed concentration of protein (0.05 μM RNase A or S-protein), S-peptide (0.4 μM) and **1a** (100 μM). Raw data for Figure 2d.

	V_0 (μg ml^{-1} s^{-1})			
**C_{RNA} (μg/ml)**	**RNase A**	**S-peptide**	**1a**	**S-protein**
**50**	0.077 ± 0.003	0.057 ± 0.002	0.016 ± 0.001	0.038 ± 0.014
**75**	0.129 ± 0.006	0.097 ± 0.004	0.04 ± 0.004	0.056 ± 0.015
**125**	0.215 ± 0.008	0.159 ± 0.008	0.088 ± 0.009	0.077 ± 0.015
**200**	0.302 ± 0.015	0.226 ± 0.011	0.126 ± 0.011	0.089 ± 0.014
**300**	0.394 ± 0.022	0.296 ± 0.029	0.168 ± 0.016	0.096 ± 0.021
**425**	0.475 ± 0.029	0.362 ± 0.037	0.211 ± 0.032	0.112 ± 0.007
**575**	0.554 ± 0.037	0.431 ± 0.050	0.254 ± 0.053	0.124 ± 0.088

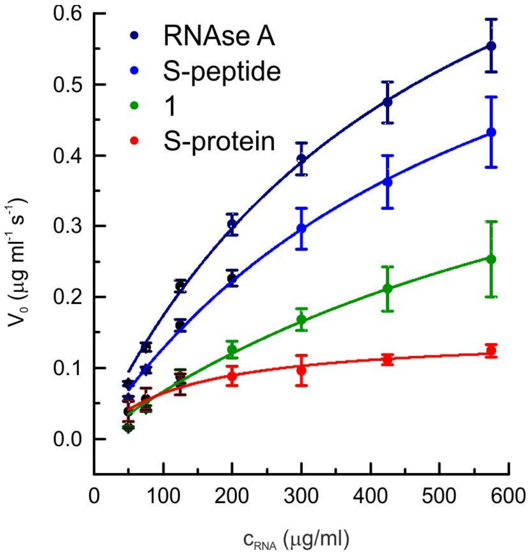

Supplementary Figure S10: Michaelis-Menten plots a fixed concentration of protein (0.05 μM RNase A or S-protein), S-peptide (0.4 μM) and **1a** (100 μM)

Table S5 ITC data for mimetic **1a**

	n	K_D (μM)	ΔH (kcal/mol)	ΔS (cal/molK)
**1a**	0.089	1.85 ± 0.97	-19.04 ± 1.90	-35.2

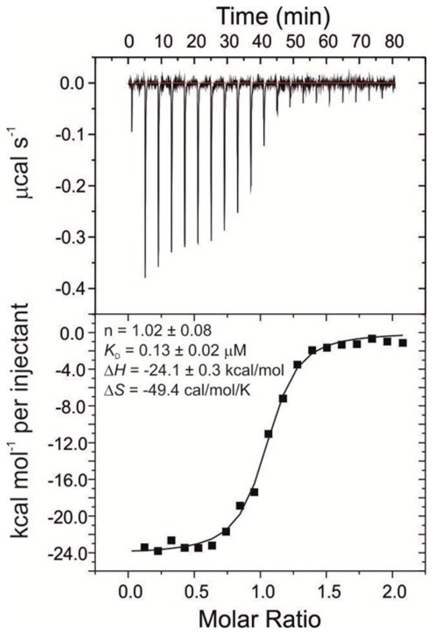

Supplementary Figure S11: ITC raw data (upper) and enthalpogram (lower) for S-peptide titrated to S-protein in 50 mM Tris, 100 mM NaCl pH 6.5 buffer. 20 μM S-protein was used in the cell titrated with 200 μM S-peptide from the syringe at 25°C.

Docking experiments

a)

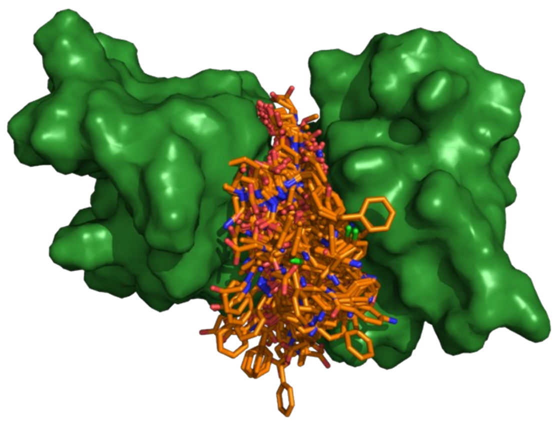

b) c) d)

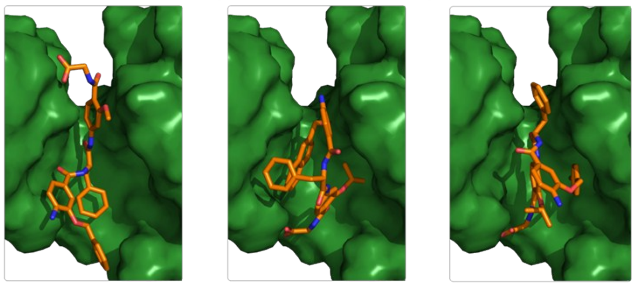

e)

	C-term to RNA	N-term to RNA	in RNA binding pocket
**1a**	75%	25%	0%
**4**	100 %	0%	0%
**12**	63%	0%	37%

Supplementary Figure S12: a) Conformational search was carried out on **1, 4** and **12,** the resulted 123 lowest energy conformers were docked against the S-protein (4YGW) using rigid sampling for docking. High score poses in the S-protein binding groove were generated for the three series of ligands with the majority binding to the S-peptide binding site having different orientations relative to the RNA binding site. Representative examples of different orientations docked to S-protein: b) ligand **12** N-terminus pointing toward RNA binding site, c) ligand **4** C-terminus pointing toward

RNA binding site, d) ligand **1a** random orientation. e) Distribution of the different orientations of the top 20% of the lowest energy poses.

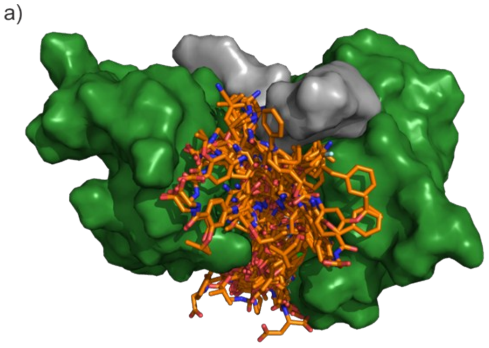

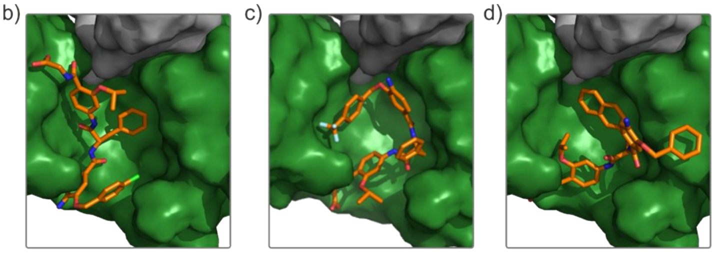

e)

C-term to RNA	N-term to RNA	Random orientation
24%	52%	24%

Supplementary Figure S13. a) hybrid mimetics were docked to S-protein/oligonucleotide complex (1RCN) using flexible sampling for docking. The lowest energy pose of each mimetic is docked in a different orientation but remains in the S-peptide binding groove. Representative orientations of the hybrid mimetics b) ligand **1a** C terminus pointing towards RNA, c) ligand **3** N terminus pointing towards RNA, d) ligand **19** random orientation. e) Distribution of the different orientations of the docked mimetics.

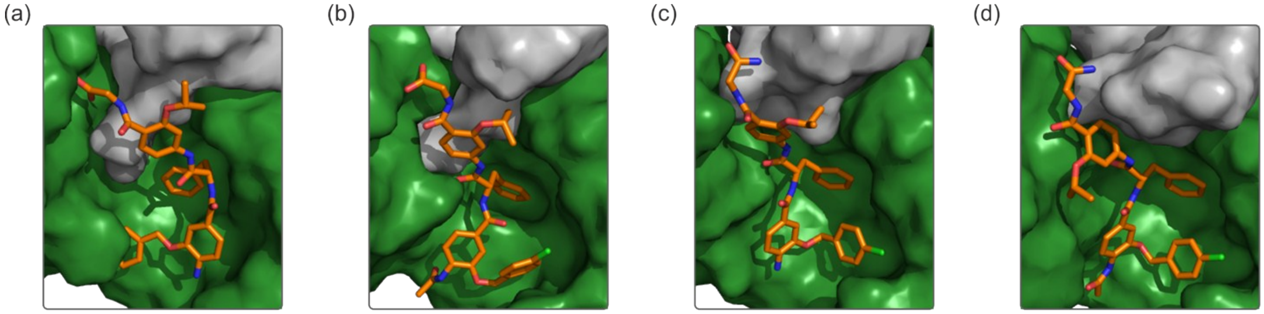

(e)

	C-term to RNA	N-term to RNA
**1a**	50%	50%
**1b**	100%	0%
**1c**	100%	0%
**1d**	100%	0%

Supplementary Figure S14. Protected variants of mimetic **1** docked against S-protein in complex with the oligonucleotide fragment using flexible sampling for docking followed by induced fit optimization. Lowest energy poses of a) **1a,** b) **1b,** c) **1c,** d) **1d.** e) Distribution of the different orientations of the docked mimetics of the top 20% of the lowest energy poses and all the poses (in brackets).

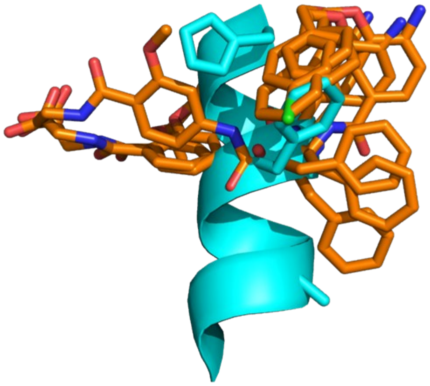

Supplementary Figure S15. Overlay of the S-peptide with the lowest energy poses of RNase S bound mimetics **1a**, **4** and **12** using flexible docking optimized by induced fit (possess taken from Fig. 4e-f; in interpreting the overlay, flexible docking permits both movement of the ligand and the protein)

Supplementary Methods

Preparation of S protein

20 mg RNase A (Sigma Aldrich) was dissolved in 1 ml 50 mM HEPES buffer (pH 8) and 100 ml subtilisin added from 1 mg/ml stock solution (protein to enzyme W/W ratio: 1:200), digestion was carried out on ice overnight. Cleavage was confirmed using high resolution mass spectrometry. To destroy subtilisin the pH was adjusted to 3 and the solution was kept on ice for an hour before purification. Protein S was purified using mass directed RP-HPLC on Phenomenex Proteo column using a gradient of 25-45 % B over 45 minutes (eluent A: H2O + 0,1% formic acid, B: ACN + 0,1% formic acid). Fractions were collected based on m/z: 1282 and 1442. Fractions containing protein S were pooled together and freezedried. Solid protein S was dissolved 50 mM Tris, 100 mM NaCl, pH 6.7 and dialysed overnight. Concentration was determined in 6M urea using the extinction coefficient of 9440 M-1 cm-1, and protein molecular weight was confirmed using high resolution mass spectrometry: calculated mw: 11533.96 (including 4 disulphide bridges), measured mw: 11533.30.

General procedure for RNA degradation assays

RNA degradation assays[9] were performed in 96-well black plates (Greiner Bio-one). Each experiment was run in triplicate and the fluorescence was measured using a Perkin Elmer EnVisionTM 2103 MultiLabel plate reader, with an excitation and emission at 510 nm and at 600 nm.[7] The buffer used in this enzymatic assay was Tris buffer (50 mM of Tris, 100 mM of NaCl, pH = 6.5). Before each experiment, RNA (RNA from Baker's yeast, Alfa Aesar) was dissolved in Tris buffer to 1 mg/ml concentration and kept on ice for an hour with vigorous shaking to fully dissolve, then Ethidium Bromide (Biorad) was added to a final concentration of 10 μg/ml.

The adequate quantity of buffer was first added to each well to reach a total volume of 200 μL. Then a variable amount of compounds (1 mM stock solution in buffer) was added to each well, followed by a variable amount of EtBr/RNA then S-protein (0.5 μM stock solution in buffer). Plates were immediately (10 seconds) read after the addition of EtBr-labelled RNA and every 30 sec for 45 min of incubation at a controlled temperature of 25 °C. Remaining fluorescence values were calculated using the 45 min time-points.

Two positive controls were performed for each run:

- 20 µL of RNase A (0.5 μM stock solution in buffer) and 30 µL of RNA are mixed in 150 µL of buffer.
- 20 µL of WT-S-peptide (4 µM stock solution), S-protein (0.5 μM stock solution in buffer) and 30 µL of RNA are mixed in 130 µL of buffer.

Several negative controls were performed for each experiment:

- A negative control was run to monitor the fluorescence of EtBr-labelled RNA: 30 µL of RNA and 170 µL of buffer.

- A negative control was run to monitor the basal activity of S-protein itself: 20 µL of S-protein (0.5 µM stock solution in buffer) and 30 µL of RNA were mixed in 150 µL of buffer.
- Negative controls were also run for each compound. The highest amount of compounds used within the assay (20 – 80 µL) and the highest amount of RNA used within the assay (30 – 80 µL) were mixed in the adequate amount of buffer.

Supplementary Table S7: General design of RNA degradation assays. Blue: negative controls, Pale red: positive controls, Green: experiments. Each case represents a triplicate.

Buffer	Buffer<br>RNA	Buffer<br>RNA<br>S-protein	Buffer<br>RNA<br>RNase A	Buffer<br>RNA<br>S-protein<br>S-peptide
Buffer<br>RNA<br>Compound X	Buffer<br>RNA<br>Compound X<br>S-protein			
Buffer<br>RNA<br>Compound Y	Buffer<br>RNA<br>Compound Y<br>S-protein			
Buffer<br>RNA<br>Compound Z	Buffer<br>RNA<br>Compound Z<br>S-protein			

Library of mimetics

In the experiment wells, 130µL of buffer, 20µL of compounds (1 mM stock solution in buffer with 10% of DMSO), 20 µL of S-protein (0.5 µM stock solution in buffer) and 30 µL of EtBr-labelled RNA (1 mg/mL in water) were mixed and the plate was read immediately.

Dose-response of best mimetics

In the experiment wells, the adequate amount of buffer to reach a total volume of 200µL, the adequate amount of compounds (40, 20, 10, 5 µL for 200, 150, 100, 75, 50, 25 µM of compound from a 1 mM stock solution in buffer with 10% of DMSO and 20, 10, 5, 2.5, 1.25 µL for 10, 5, 1, 0.5, 0.2 µM of compound from a 100 µM stock solution in buffer with 0.1% of DMSO), 20µL of S protein and 30µL of EtBr-labelled RNA were mixed and the plate was read immediately.

Kinetic data

In the experiment wells, the adequate amount of buffer was added to reach a total volume of 200µL, then 20 µL of compounds (1 mM stock solution of mimetic in buffer with 10% of DMSO or 4 µM stock solution of peptide S) and adequate amount of EtBr-labelled RNA for 50, 75, 125, 200, 300, 425, 575 µg/ml of RNA concentration and 20µL of S protein (0.5

μM stock in buffer) were mixed. The plate was read immediately and every 30 sec for 45 min of incubation at a controlled temperature of 25 °C. Final concentration of compounds in the experiment wells: S-protein/RNase A 0.05 μM, S-peptide: 0.4 μM, mimetic: 0.1 mM. A negative control was run to monitor the fluorescence of EtBr-labelled RNA for each RNA concentration, which was used to determine the amount of degraded RNA at every time-point.
Data analysis: A calibration curve was prepared by plotting fluorescence intensity against known RNA concentrations and this was then used to determine the remaining RNA concentration at each time-point in the measurement wells. Degraded RNA concentration was calculated by subtracting the remaining RNA from the initial RNA concentration and then these were plotted against time yielding the progression curves. Progression curves were fitted using Dynafit software[10] to Michaelis Menten mechanistic model in order to determine initial velocity (V_0). Initial velocities then were plotted against RNA concentration and the curves were fitted to Michaelis-Menten model in Origin Pro 9. The first 3 points of the Michaelis-Menten plots were fitted to a linear function in Origin Pro 9 and the resultant slopes were used to determine the V_{max}/K_M values.

Dose-response for SAR activity

Four mimetics were tested at a single concentration in the assay: **1a**, **1b**, **1c** and **1d**
In the experiment wells, the adequate amount of buffer to reach a total volume of 200μL, 20μL of compounds (from 1 mM stock solution in buffer with 10% of DMSO), 20μL of S protein and 30μL of EtBr-labelled RNA were mixed and the plate was read immediately.
The two active mimetics, **1a**, **1b**, were tested titrated in the assay.
In the experiment wells, the adequate amount of buffer to reach a total volume of 200μL, the adequate amount of compounds (40, 20, 10, 5 μL for 200, 100, 50, 25 μM of compound from 1 mM stock solution in buffer with 10% of DMSO and 20, 10, 5, 2.5, 1.25 μL for 1.25, 0.62, 0.31, 0.016, 0.08 μM of compound from 12.5 μM stock solution in buffer with 0.1% of DMSO), 20μL of S protein and 30μL of EtBr-labelled RNA were mixed and the plate was read immediately.

Regulation of S-protein activity

The reactivation of S-protein activity was regulated using the *h*DM2 protein at a single concentration of 10μM. A compromise was made between the affinity to *h*DM2 and the ability to restore S-protein activity to select the right mimetic **1** was therefore chosen.

A classical series of experiments was conducted with S-protein itself, RNase A, a mixture of S-protein and S-peptide, a mixture of S-protein and **1** as it was detailed above. The exact same series was conducted in presence of *h*DM2 (20 μL of *h*DM2 from a 100 μM stock solution in buffer) by incubating *h*DM2 and the peptide or the mimetic for 1hr:
In the experiment wells, the adequate amount of buffer to reach a total volume of 200μL, 20 μL of the compound (S-peptide from a 4μM stock solution, **1** from a 1 mM stock solution) and 20 μL of *h*DM2 (from a 100 μM stock solution in buffer) were mixed and incubated for 1 hr. Then 20μL of S-protein and 30μL of EtBr-labelled RNA were added and the plate was read immediately.

A titration of hDM2 was conducted in presence of **1a**, S-protein and labelled RNA: In the experiment wells, the adequate amount of buffer to reach a total volume of 200µL, 20 µL of **1a** and the adequate quantity of *h*DM2 (20, 6.6, 2 µL for 10, 3.3, 1 µM of *h*DM2 from a 100 µM stock solution in buffer and 20, 10, 5, 2.5 µL for 500, 250, 125, 75 nM of compound from 5 µM stock solution in buffer) were mixed and incubated for 1 hr. Then 20µL of S-protein and 30µL of EtBr-labelled RNA were added and the plate was read immediately. For this assay, an additional negative control was performed: 130µL of buffer, 20 µL of *h*DM2 (100 µM stock solution in buffer), 20 µL of compound (1 mM stock solution) and 30 µL of EtBr-labelled RNA were mixed.

Isothermal titration calorimetry

ITC measurements were performed on Microcal ITC200i instrument (Malvern) using 50 mM Tris, 100 mM NaCl pH 6.5 buffer. For proteomimetic **1a** a reverse titration was performed. Proteomimetic **1a** 0.5 mM in 10 % DMSO and S-protein was dialysed into 50 mM Tris, 100 mM NaCl, pH 6.5 using 0.5 kDa cellulose acetate membrane. The concentration of the dialysed sample was determined using an LC-MS calibration curve. 2.5 µL injections of 0.1 mM S-protein from the syringe was injected to the ITC cell containing 100 µM mimetic at 37°C, S-protein dilution heats were measured injecting the protein to the cell containing buffer and subtracted from the measurement. For S-peptide 20 µM S-protein was used in the cell titrated with 200 µM peptide from the syringe at 25°C using 2 µl injections. Peptide dilution heats were measured injecting the peptides to the cell containing buffer and substracted from the measurement. Data were analysed using Microcal Origin 8 and fitted to a one-binding site model.

Native MS

Spectra were recorded on a Bruker Maxis Impact Q-TOF mass spectrometer equipped with a CaptiveSpray™ LC-MS ion source. Samples were prepared in 20 mM NH_4Ac buffer (pH 6.5) using 2 µM S-protein with or without 10 µM **1a** and variable concentration of S-peptide (0.25, 0.5, 0.75, 1, 1.25, 1.5, 2.5, 5 µM). Measurements were carried out at 3 µL/min flow rate using a manual syringe pump. Ion source settings were the following: 1200 V capillary voltage, 150 °C drying gas temperature at 5 l/min flow rate, using 10 V collision energy in the collision cell. 240 scans were acquired over 4 minutes and averaged.

Docking experiments

Protein preparation

Two S-protein models were prepared from crystal structures:

- the crystal structure of S-protein$_{22\text{-}124}$ in complex with a stabilized S-peptide (PDB ID: 4YGW). The stabilized S-peptide$_{1\text{-}15}$ was removed from the structure.

- the crystal structure of RNAse A in complex with the oligonucleotide dApTpApApG (PDB ID: 1RCN). The fragment corresponding to the S-peptide$_{1-21}$ was manually removed from the RNAse A sequence; mimicking the subtilisin hydrolysis and giving the S-protein$_{22-124}$. The oligonucleotide fragment was kept in place.

The protein models were prepared and minimized at pH = 6.5, using the Protein Preparation wizard. The Hδ protonation state of H119 was imposed to respect the active state of the S-protein.

Grid generation

The minimized receptors without and with the oligonucleotide fragment were used as to generate the Receptor Grid. The enclosing box, confining the docked ligands, was centred on the centroid of the S-peptide, with a size of 36 Å^3. No restraints, forbidden area nor rewards were defined leaving a maximum of freedom for the docking experiment. All residues (Y(25), S(32), T(45), S(50), S(59), T(70), T(73), S(80), T(82), Y(92), Y(97), T(100), Y(115), S(123), and T(3)) presenting hydroxyl and thiol groups were allowed to rotate during calculation.

Ligand preparation: Molecular Mechanics calculation

A hybrid MCMM conformational search was carried out on **1a, 4** and **12** in a water medium using Macromodel software package from Schrödinger and the MMFF force field with no restraint. The ionization state of the compounds were drawn as a Cter carboxylate and an Nter amine according to the pH of the assay (pH = 6.5) (Supplementary Supplementary Figure S16).

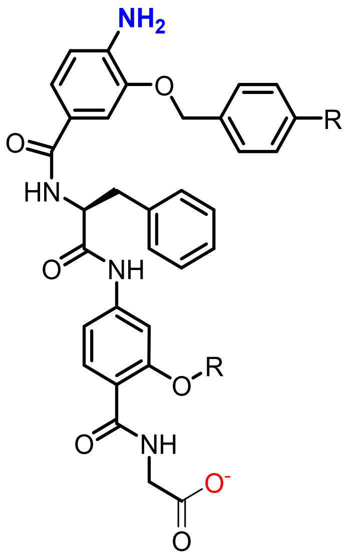

Supplementary Figure S16: General scheme showing the ionization state of the hybrid trimers used for modelling

The calculation was run to generate 10 000 conformer sets, of which the lowest energy conformers up to 10 kJ.mol^{-1} (29 conformers for **1a**, 41 for **4** and 53 **12**, 123 conformers in total) were retained for docking experiments. The lowest energy conformers belong to the same family of conformers and are well superimposed with the first lowest energy conformers.

Docking and score

The 123 lowest energy conformers of **1a**, **4**, and **12** were docked with the prepared S-protein (from 4YGW) and S-protein in complex with dApTpApApG (1RCN) using Glide XP method.[11-13] All of the input ligands gave a pose in the S-protein binding groove. The docking poses of the three candidates found to restore best S-protein activity, **1a**, **4** and **12**, were refined using the induced fit docking against S-protein in complex with the oligonucleotide. Side chains of S-protein binding groove were automatically trimmed (based on B-factor) during docking calculation and restored for the final refinement.
The library of hybrid mimetics were docked as flexible ligands against the S-protein in complex with the oligonucleotide fragment using Glide XP (extra precision) method.[11-13]
The three protected mimetics derived from **1a**, **1b**, **1c**, and **1d** were docked as flexible ligands against S-protein in complex with the oligonucleotide fragment using Glide XP method[11-13] and refined using induced fit docking (Fig S7).

Fluorescence anisotropy

The fluorescein-labelled p53$_{15-31}$–Flu[3,4] was purchased from peptide protein research Ltd.

Fluorescence anisotropy assays were performed in 384-well black plates (Greiner Bio-one). Each experiment was run in triplicate and fluorescence anisotropy was measured using a Perkin Elmer EnVisionTM 2103 MultiLabel plate reader. Fluorescein labelled peptides used an excitation wavelength of 480 nm (30 nm bandwidth) and emission wavelength of 535 nm (40 nm bandwidth) (polarised dichroic mirror at 505 nm).

Experiments were performed in 50 mM phosphate buffer at pH 7.5, containing 200 mM NaCl and 0.02 mg.mL^{-1} bovine serum albumin (BSA).

20 µL of assay buffer were first added to each well. 20 µL of a solution of α-helix mimetics (1 mM in 90:10 (v/v) assay buffer: DMSO) were added to the first column. The solution was well mixed and 20 µL were taken out and added to the next column and so on. This operation consisted on serial dilution of the peptides across the plate (24-points, 1/2 serial dilution). The tracer peptide and the targeted protein were added to each well to give a final concentration of 25 nM and 150 nM, respectively.
For control wells in both assays, the tracer peptide was replaced with an identical volume of assay buffer. The total volume in each well was 60 µL. Plates were read after 1 h of incubation at room temperature.

The data for both the P (perpendicular intensity) and S (parallel (same) intensity) channels, resulting from this measurement and corrected by subtracting the

corresponding control wells, were used to calculate the intensity and anisotropy for each well following Equations 1 and 2:

$$I = (2PG + S) \quad \text{(Equation 1)}$$

$$r = \frac{S - PG}{I} \quad \text{(Equation 2)}$$

Where I is the total intensity, G is an instrument factor which was set to 1 for all experiments and r is the anisotropy.

The average anisotropy (across three experimental replicates) and the standard deviation of these values were then calculated and fit to a sigmoidal logistic model (Equation 3) using OriginPro 9.0 which provided the IC50 and error values.

$$y = r_{max} + \frac{r_{min} - r_{max}}{1 + (\frac{x}{x_0})^p} \quad \text{(Equation 3)}$$

Synthesis of mimetics

General Comments

All amino acids and resins were purchased from either Novabiochem (Merck) or Sigma-Aldrich. All amino acids were *N*-Fmoc protected and side chains were protected with Boc (Lys, Trp); O*t*Bu (Asp, Glu, Ser, Thr); Trt (Cys, Asn, Gln); Pbf (Arg). BID-Cys and BID-hCys peptides were purchased from Severn Biotech Ltd. Syntheses of peptides were carried out in the Liberty CEM Peptide Synthesiser with or without microwave assistance. All solvents used were of ACS grade from Sigma-Aldrich.

UV absorbance analyses were recorded in a 1 mm path-length quartz cuvette on a Perkin Elmer Lambda 900 UV/VIS/NIR spectrometer.

High-resolution mass spectrometry (HRMS) data was recorded using electrospray ionization in positive mode (ESI^+) with a Bruker MaXis Impact spectrometer.

LC-MS analyses were conducted on a Thermo Scientific Ultimate 3000 using $UHPLC^+$ technology. All experiments were run through a Kinetex C-18 50x2.1 mm LC-Column, 2.6 μm particle size, for small molecules using a 5-95% gradient (acetonitrile:water, 0.1% formic acid) over 1.7 min and an Aeris Peptide XB-C18 100x2.1 mm LC-Column, 2.6 μm particle size, for peptides using a 5-95% gradient (acetonitrile:water, 0.1% formic acid) over 3.6 min for positive ion spectra. Samples were ionized by electrospray.

Analytical HPLC experiments were performed using an Agilent 1290 Infinity LC series system equipped with an Ascentis Express Peptide ES-C18 100x2.1 mm column, 2.7 μm particle size on a 5-95% gradient (acetonitrile:water, 0.1% formic acid) over 5 min.

CD data were recorded using an Applied Photophysics Chirascan Instrument and Software in a 1 mm path-length quartz cuvette. For each scan, the following parameters were used: 180-260 nm range; point time 1 s; 1 nm per point; step = 1; bandwidth 5 nm; temperature 20 °C. Scans were performed in triplicate.

Fourier-transform infrared absorption spectroscopy (IR) was performed on Bruker Platinum-ATR system equipped with an Alpha FT-IR spectrometer. Maximum absorbances are reported for significant bands in cm^{-1}.

General procedure for the synthesis of S peptide

S-peptide (1 eq., 0.1 mmol) was synthesized on Rink Amide MBHA resin (Loading Capacity 0.36 mmol g^{-1}) using DIC (5 eq., 0.5 mmol) and Oxyma pure (6 eq., 0.6 mmol) in DMF for amide coupling, and a solution of DMF/piperidine/formic acid:75/20/5 for the deprotection. Each amino acid (5 eq. 0.5 mmol) was coupled using the standard microwave method (75 °C, 0 W for 5 sec; 80 °C, 130 W for 20 sec; 86 °C, 65 W for 10 sec and 90 °C, 25 W for 240 sec) and deprotected using the standard microwave method (45 °C, 0 W for 5 sec; 78 °C, 130 W for 20 sec; 88 °C, 65 W for 10 sec and 90 °C, 25 W for 60 sec). Special care was taken for aspartic acid deprotection (75 °C, 50 W for 180 sec) and for arginine coupling (75 °C, 25 W for 1200 sec) and deprotection (75 °C, 50 W for 180 sec).

After the final amino acid coupling and deprotection, a solution of acetic anhydride (10 equiv.) and DIPEA (10 equiv.) in DMF (2 mL) was manually transferred to the resin. After 2 h, the resin was drained, washed with DMF (5 × 2 mL × 2 min), CH_2Cl_2 (5 × 2 mL × 2 min) and then Et_2O (3 × 2 mL × 2 min).

Peptides were simultaneously cleaved from the resin and side-chain deprotected using the cleavage "reagent H" TFA:H_2O:phenol:thioanisole:dimethylsulfide:1,2-ethanediol:ammonium iodide, 81:3:5:5:2:2.5:1.5 (8 mL) for 2 h. Reagent H prevented Methionine oxidation. The resin was washed with fresh TFA (2 mL) and the solution concentrated *in vacuo*. The resulting oils were precipitated with ice-cold ether (5 mL) and placed in a centrifuge (3000 rpm × 3 min). The supernatants were removed, the precipitate rinsed with ice-cold ether (3 × 10 mL) and dried in vacuo.

Crude peptide were suspended in DMF at an approximate concentration of 10 mg.mL^{-1}. Purification was performed using a UV HPLC with a Jupiter Proteo preparative column on gradient of acetonitrile/water (with 0.1% formic acid) for 35 min at a flow rate of 10 mL min^{-1}. Fractions were checked by LCMS, concentrated *in vacuo* and lyophilised.

LCMS characterization of S-peptide

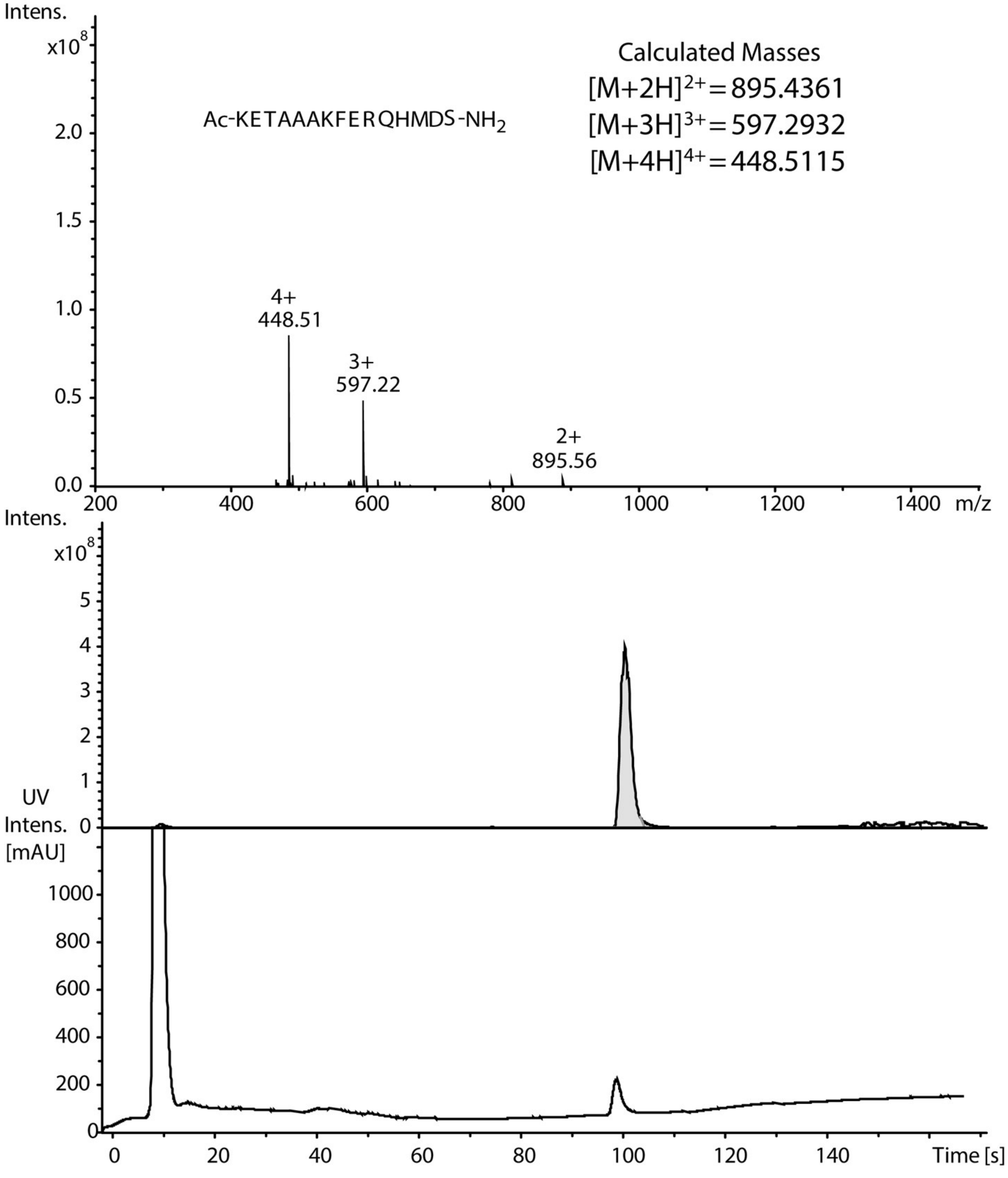

Supplementary Figure S17: Characterisation of S-peptide. Top: MS-spectrum; middle: MS traces; down: UV-LCMS trace (200-300 nm).

General procedure for the synthesis of mimetics

A generic procedure was followed adapting the previously reported solution and solid phase syntheses of *O*-alkylated oligobenzamides, *N*-alkylated oligobenzamides and hybrid mimetics.[1,8,14-16] The procedure is described as below and illustrated in Supplementary Scheme S1:

a. The Fmoc-Gly-Wang resin (1 eq., 0.1 mmol, 0.70 mmol.g^{-1}) was used for hybrid mimetics **1-8, 1a, 1b, 10-33, 36,38, 42-47, 49-54**, Fmoc-Lys-Wang for **9**, Fmoc-Asp-Wang for **34, 41**; Fmoc-Pro-Wang for **9**, **35, 39, 40**, Fmoc-Leu-Wang for **48** and the Rink-Gly-Amide MBHA (1 eq., 0.1 mmol, 0.50 mmol.g-1) was used for hybrid mimetics **1c, 1d**. Resins were swelled in anhydrous DMF/DCM:1/1 for 15 min and deprotected with a solution of DMF/piperidine:80/20.
2-*O*-alkylated monomer (5 eq., 0.5 mmol) in anhydrous DMF was pre-activated with HCTU (6 eq., 0.6 mmol) and DIPEA (8 eq., 0.8 mmol) for at least 1 hr at room temperature prior coupling. The activated 2-*O*-alkylated monomer was coupled twice using a double coupling microwave method (60 °C, 25 W for 1800 sec).

b. The coupled monomer or amino acid was deprotected twice (75 °C, 50 W for 180 sec) with a solution of DMF/piperidine:80/20.

c. The central amino acid (5 eq., 0.5 mmol) in anhydrous DMF was pre-activated with HCTU (6 eq., 0.6 mmol) and DIPEA (8 eq., 0.8 mmol) for at least 1 hr at room temperature prior coupling. The activated amino acid was coupled twice using a double standard microwave method (75 °C, 0 W for 5 sec; 80 °C, 130 W for 20 sec; 86 °C, 65 W for 10 sec and 90 °C, 25 W for 240 sec) and a single coupling method (75 °C, 25 W for 35 min).

d. The acetylation step was carried out manually. A solution of acetic anhydride (10 eq., 1 mmol) and DIPEA (10 eq., 1 mmol) in DMF (2 mL) was transferred to the resin. After 2 h, the resin was drained, washed with DMF (5 × 2 mL × 2 min), CH_2Cl_2 (5 × 2 mL × 2 min) and then Et_2O (3 × 2 mL × 2 min).

e. The final cleavage and deprotection were carried out manually. Hybrid mimetics were simultaneously cleaved from the resin and side-chain deprotected with a solution of CH_2Cl_2/TFA:1/1 (10 mL) for 4 hrs. The resin was washed with fresh TFA (2 mL) and the solution concentrated *in vacuo*. The resulting oils were concentrated under reduced pressure and the precipitate dried in vacuo.

Crude hybrid mimetics were suspended in DMF at an approximate concentration of 10 mg.mL^{-1}. Purification was performed using a UV HPLC with a Jupiter Proteo preparative column on gradient of acetonitrile/water (with 0.1% formic acid) for 35 min at a flow rate of 10 mL min^{-1}. Fractions were checked by LCMS, concentrated *in vacuo* and lyophilised.

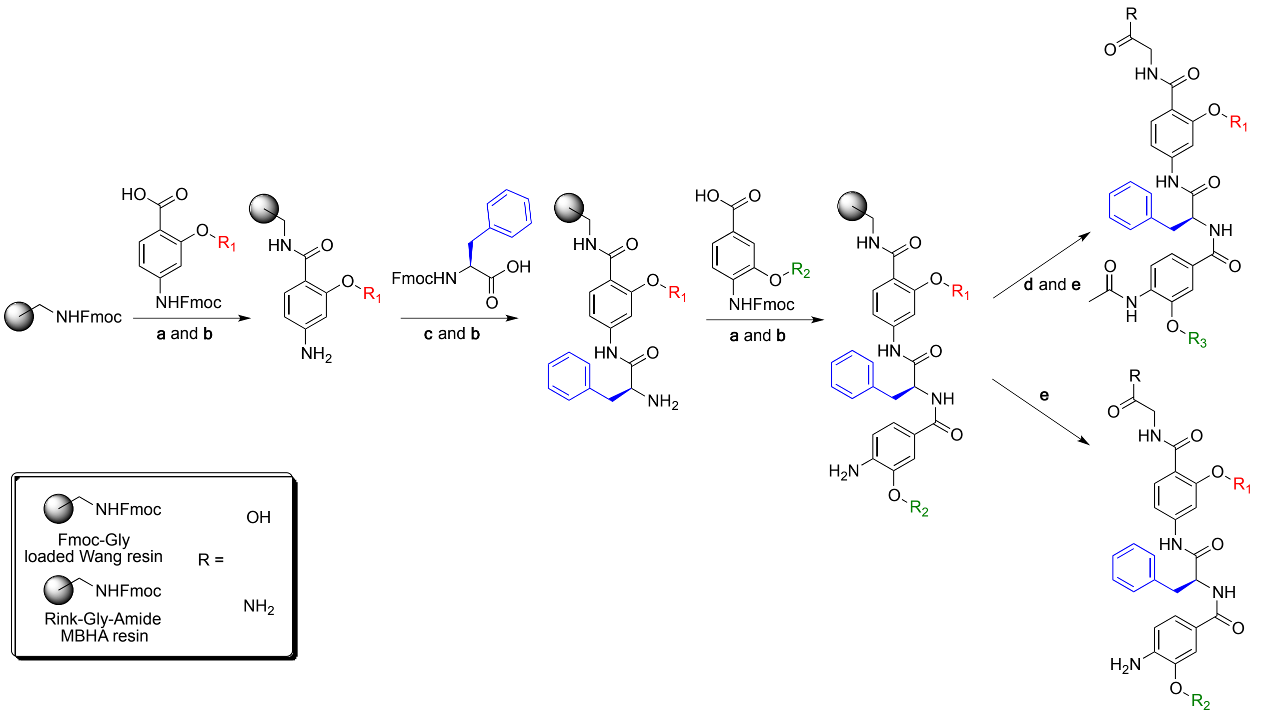

Supplementary Scheme S1: Strategy for the general synthesis of hybrid mimetics with different N- and C-termini: N-Ac, NH_2, -COOH, -$CONH_2$

Numbering system for proteomimetic scaffolds

To simplify the numbering and NMR assignment of the different proteomimetic scaffolds, a sequential nomenclature and numbering system has been created, where each of the monomer building blocks is considered separately. Assignment is as follows:

- The naming proceeds from *N* to *C* terminus. Following this order, each monomer is assigned a number corresponding to its position on the chain. This number will be added as a prefix to the individual carbon number for differentiation.
- In the 3-*O*-alkylated and hybrid scaffolds the *O*-alkylated monomers are named as [*R*-(*n*-HABA)], where *R* is the alkoxy side-chain, *n*- indicates the position of the alkoxy moiety on the aromatic ring (*e.g.* for a 2-*O*-alkylated monomer *n* = 2) and HABA is the acronym for Hydroxy Amino Benzoic Acid.
- Each alkylated monomer is numbered using the standard system, where the substituents are assigned to the lowest number, in the case of originally symmetrical monomers one of the side chains is differentiated with an apostrophe (′) after the carbon number. Side-chain assignment follows a peptide nomenclature pattern in which the carbon attached to the alkoxy oxygen is assigned as Cα and the numbering of the aliphatic part of the side chain continues with Cβ, etc. In the case of aromatic side chains, the aromatic carbons are numbered CAr1, CAr2, etc. The Fmoc carbons are differentiated by the prefix F; the CH_2 group is numbered as CFα, the neighbouring CH is CFβ, and the aromatic positions go from CF-Ar2 to CF-Ar5.
- Amino acids are numbered using the standard convention.
- The numbering of the protons is based on the carbon numbering system.

Examples of the numbering system for the proteomimetic scaffolds and monomer building blocks are given below

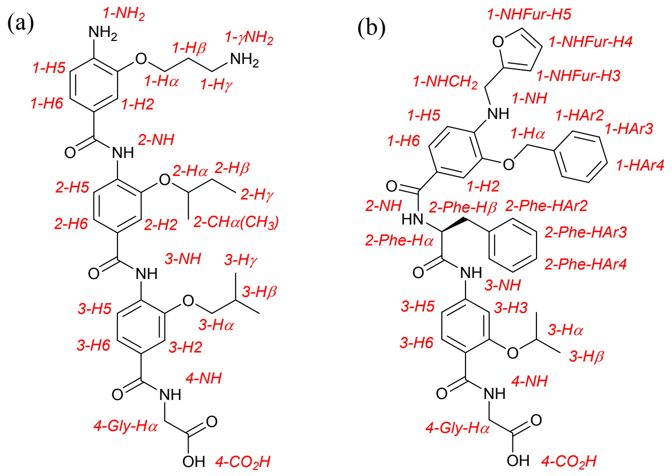

Supplementary Figure S18 Numbering system (a) 3-*O*-alkylated scaffold (b) Hybrid scaffold

The *O*-alkylated monomers employed in the hybrid scaffold syntheses were obtained using the same general procedures as for the 3-*O*-alkylated monomers unless stated otherwise. Only the characterization of novel monomers has been included here; the monomers which have been routinely synthesised and reported previously in the group have not been included.

LCMS characterization of mimetics 1a-d

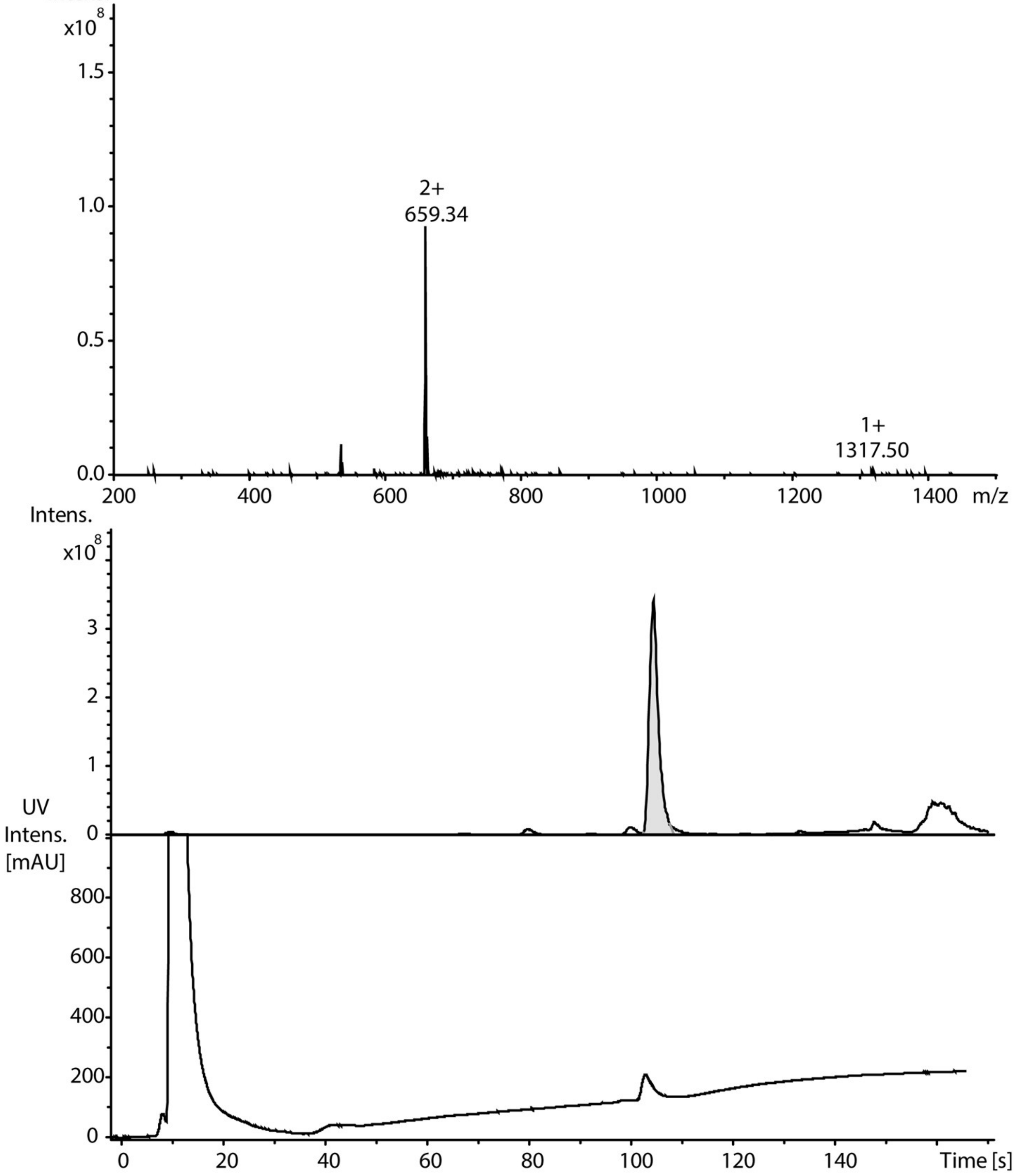

Supplementary Figure S19: Characterisation of 1a. Top: MS-spectrum; middle: MS traces; down: UV-LCMS trace (200-300 nm).

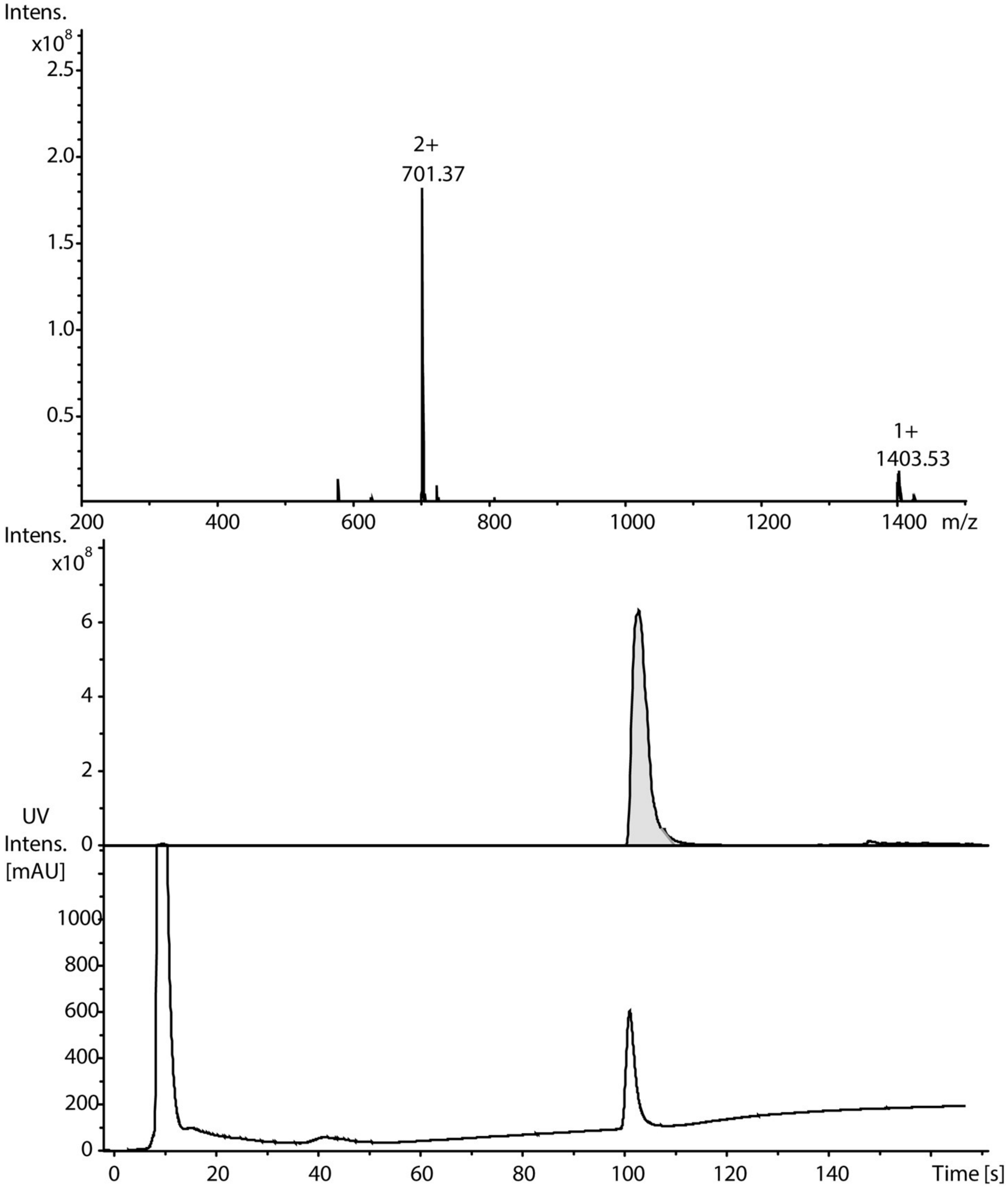

Supplementary Figure S20: Characterisation of 1b. Top: MS-spectrum; middle: MS traces; down: UV-LCMS trace (200-300 nm).

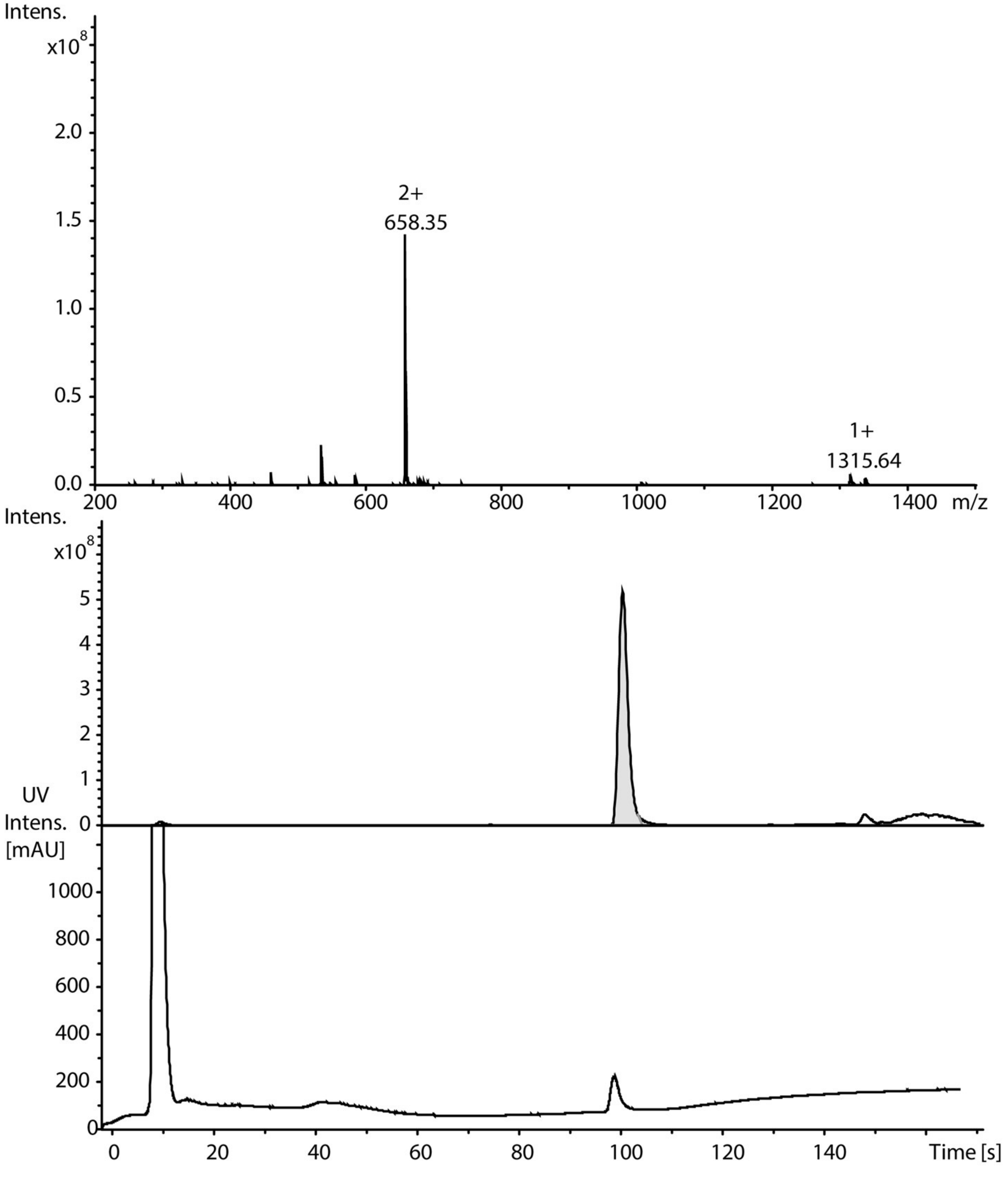

Supplementary Figure S21: Characterisation of 1c. Top: MS-spectrum; middle: MS traces; down: UV-LCMS trace (200-300 nm).

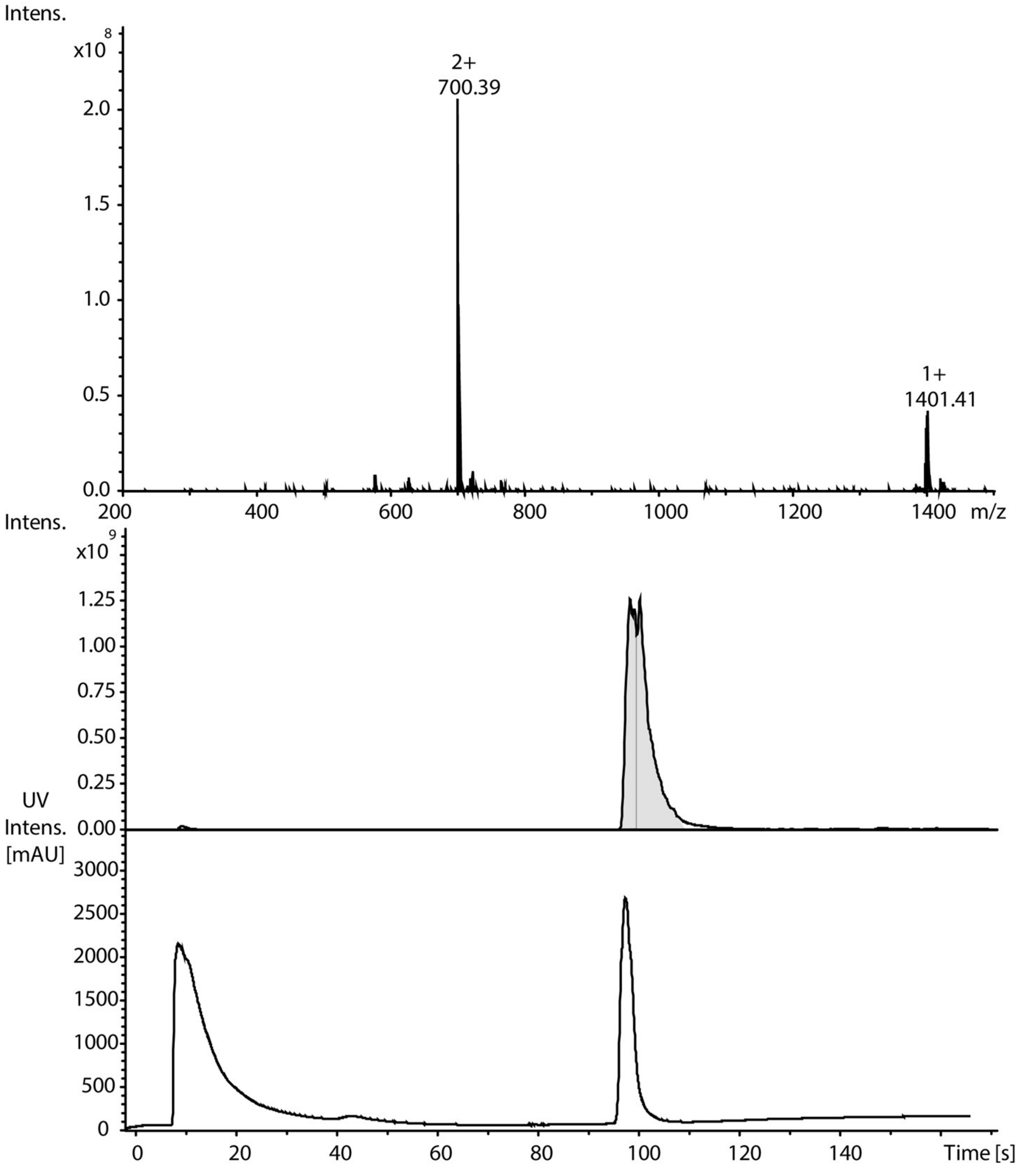

Supplementary Figure S22: Characterisation of 1d. Top: MS-spectrum; middle: MS traces; down: UV-LCMS trace (200-300 nm).

Syntheses and Characterization for Previously unpublished Helix Mimetics

Only characterization of novel monomers has been included in the experimental section; monomers which have been routinely synthesised and reported previously have not been included.[17]

Procedure A: RBr Alkylation To a stirred solution of methyl-3-hydroxy-4-nitrobenzoate (1 equiv) and potassium carbonate (3 equiv) in DMF (20 mL / g), was added RBr (1.2 equiv) and the resulting mixture stirred at 50 ºC overnight under a nitrogen atmosphere. Further portions of the RBr were added when the reaction was shown incomplete by TLC. The resultant mixture was allowed to cool to rt, poured into water (40 mL / g) and extracted with ethyl acetate. The combined organic fractions were thoroughly washed with water and further washed with brine, dried with $MgSO_4$ and evaporated to dryness.

Procedure B: Mitsunobu A stirred solution containing methyl-3-hydroxy-4-nitrobenzoate (1 equiv), ROH (1.1 equiv) and triphenylphosphine (1.5 equiv) in anhydrous tetrahydrofuran (30 mL / g) was cooled to 0 °C. Diisopropyl azodicarboxylate (1.5 equiv) was added and the resulting solution allowed to warm to rt and left stirring overnight under a nitrogen atmosphere. Organic solvents were removed under reduced pressure and the product was purified *via* column chromatography.

Procedure C: Hydrogenation A solution containing either i) nitro/ester or ii) nitro/acid (1 equiv) in methanol (20 mL / g) and palladium on carbon (10 wt %) was evacuated and flushed with nitrogen (3 times) and left under vacuum. Hydrogen was drawn into the flask and the reaction left stirring at rt overnight. On completion, the reaction mixture was filtered through a celite pad and evaporated to dryness.

Procedure D: NaOH Saponification

To a solution containing either i) amine/ester or ii) nitro/ester (1 equiv) in a 1:1 mixture of methanol: tetrahydrofuran (25 mL / g), a 10 % sodium hydroxide solution (5 mL / g) was added and the resulting mixture was allowed to stir at rt overnight. Further portions of the hydroxide solution were added when the reaction was shown incomplete by TLC. The organic solvents were removed under reduced pressure, the remaining solution was poured into water and extracted with dichloromethane (unreacted starting material). The aqueous layer was acidified *via* the addition of hydrochloric acid (conc) to pH 4 and the resulting precipitate was extracted into dichloromethane. The combined organic extracts were washed with water and brine, dried with $MgSO_4$ and evaporated to dryness.

Procedure E: Fmoc protection

A solution of amine/acid (1 equiv) in anhydrous tetrahydrofuran (20 mL / g) was held at a reflux under a nitrogen atmosphere. A solution of fluorenylmethyloxycarbonyl chloride (1.5 equiv) in anhydrous tetrahydrofuran (10 mL / g) was then added dropwise and the resulting solution was stirred at reflux overnight. The reaction mixture was evaporated to dryness, the resulting solid was crystallized from a chloroform/hexane mixture and the precipitate collected *via* filtration.

Methyl 2-methoxy-4-nitrobenzoate

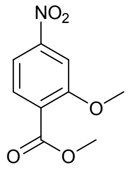

Procedure A; methyl 2-hydroxy-4-nitrobenzoate (2.0 g, 10.1 mmol), potassium carbonate (3.5 g, 25.3 mmol), in dimethylformamide (120 mL), methyl iodide (7.7 mL, 50.5 mmol). Work up afforded the title compound (2.08 g, 9.9 mmol, 98 %) as a pale yellow solid; δ_H (500 MHz, $CDCl_3$) 7.90 (d, *J* = 8.2 Hz, 1 H, *H*6), 7.85 (dd, *J* = 8.2, 1.8 Hz, 1 H, *H*5), 7.83 (d, *J* = 1.8 Hz, 1 H, *H*3), 4.01 (s, 3 H, *H*α), 3.95 (s, 3 H, CO_2CH_3); δ_C (125 MHz, $CDCl_3$) 165.3, 159.3, 150.8, 132.1, 126.0, 115.0, 107.0, 56.6, 52.6; v_{max}/cm^{-1} (solid state) = 3121, 2952, 2848, 1733, 1517, 1245, 1079, 735; ESI-HRMS found *m/z* 212.0552 $[M+H]^+$, $C_9H_{10}NO_5$ requires 212.0553.

Methyl 2-sec-butoxy-4-nitrobenzoate

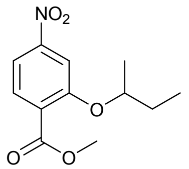

Procedure B; methyl 2-hydroxy-4-nitrobenzoate (1.0 g, 5.1 mmol), *sec*-butanol (415 mg, 5.6 mmol), triphenylphosphine (2.0 g, 7.6 mmol) in anhydrous tetrahydrofuran (40 mL) and diisopropyl azodicarboxylate (1.5 mL, 7.6 mmol). An extra portion of *sec*-butanol (1 equiv), triphenylphosphine (1 equiv) and diisopropyl azodicarboxylate (1 equiv) was added to bring the reaction to completion. The reaction mixture was purified by column chromatography (*Stationary Phase*: Silica; *Mobile Phase*: ethyl acetate/hexane, 1:1) to afford the desired product (850 mg, 3.4 mmol, 66%) as a yellow oil; δ_H (500 MHz, $CDCl_3$) 7.84 (d, *J* = 9.0 Hz, 1 H, *H*6), 7.75 – 7.81 (m, 2 H, *H*3, *H*5), 4.47 – 4.53 (m, 1 H, *H*α), 3.92 (s, 3 H, CO_2CH_3), 1.86 – 1.78 (m, 1 H, *H*β), 1.69 – 1.78 (m, 1 H, *H*β′), 1.38 (d, *J* = 6.2 Hz, 3 H, CHα(C*H*3)), 1.03 (t, *J* = 7.4 Hz, 3 H, Hγ); δ_C (125 MHz, $CDCl_3$) 165.7, 157.9, 150.6, 131.9, 127.4, 114.5, 109.1, 52.4, 33.6, 29.1, 18.8, 9.4; v_{max}/cm^{-1} (solid state) = 3108, 2973, 2880, 1736, 1526, 1346, 1245, 1077, 736; ESI-HRMS found *m/z* 254.1025 $[M+H]^+$, $C_{12}H_{16}NO_5$ requires 254.1023.

Methyl 4-amino-2-methoxybenzoate

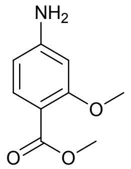

Procedure C; methyl 2-methoxy-4-nitrobenzoate (2.08 g, 9.9 mmol) in a 1:1 mixture of tetrahydrofuran/methanol (20 mL). After work up, the reaction mixture was purified by column chromatography (*Stationary Phase*: Silica; *Mobile Phase*: ethyl acetate/hexane, 3:1) to yield the title compound **3.10** (1.78 g, 9.9 mmol, quant.) as a pale orange solid; δ_H (500 MHz, $CDCl_3$) 7.75 (d, *J* = 8.5 Hz, 1 H, *H*6), 6.24 (dd, *J* = 8.5, 1.9 Hz, 1 H, *H*5), 6.21 (d, *J* = 1.9 Hz, 1 H, *H*3), 4.05 (s, broad, 2 H, N*H*2), 3.87 (s, 3 H, *H*α), 3.83 (s, 3 H, CO_2CH_3); δ_C (125 MHz, $CDCl_3$) 166.2, 161.8, 152.0, 134.2, 109.1, 106.4, 97.7, 55.8, 51.4; v_{max}/cm^{-1} (solid state) = 3461, 3364, 3236, 2948, 2837, 1698, 1606, 1434, 1254, 1217, 1086, 830, 774; ESI-HRMS found *m/z* 182.0814 $[M+H]^+$, $C_9H_{12}NO_3$ requires 182.0812.

Methyl 4-amino-2-sec-butoxybenzoate

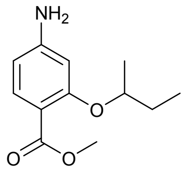

Procedure C; methyl 2-sec-butoxy-4-nitrobenzoate (850 mg, 3.4 mmol) in a 1:1 mixture of tetrahydrofuran/methanol (40 mL). Work up yielded the title compound (747 mg, 3.4 mmol, quant.) as a purple brown oil; δ_H (500 MHz, $CDCl_3$) 7.72 (d, *J* = 8.5 Hz, 1 H, *H*6), 6.23 (dd, *J* = 8.5, 2.1 Hz, 1 H, *H*5), 6.20 (d, *J* = 2.1 Hz, 1 H, *H*3), 4.27 – 4.33 (m, 1 H, *H*α), 3.97 (s, broad, 2 H, N*H*2), 3.82 (s, 3 H, CO_2CH_3), 1.74 – 1.86 (m, 1 H, *H*β), 1.62 – 1.74 (m, 1 H, *H*β′), 1.33 (d, *J* = 6.2 Hz, 3 H, CHα(C*H*3)), 1.01 (t, *J* = 7.4 Hz, 3 H, Hγ); δ_C (125 MHz, $CDCl_3$) 166.5,

160.5, 151.6, 134.2, 110.8, 106.7, 100.9, 76.5, 51.3, 29.2, 19.1, 9.6; v_{max}/cm^{-1} (solid state) = 3463, 3365, 3234, 2971, 2946, 2878, 1699, 1598, 1448, 1432, 1246, 1140, 1080, 773; ESI-HRMS found *m/z* 246.1103 $[M+Na]^+$, $C_{12}H_{17}NNaO_3$ requires 246.1101.

4-Amino-2-methoxybenzoic acid

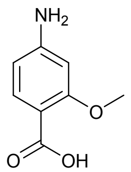

Procedure D; methyl 4-amino-2-methoxybenzoate (1.60 g, 8.8 mmol) in a 1:1 mixture of tetrahydrofuran/methanol (40 mL), 10% aqueous sodium hydroxide (30 mL). Additional 1:1 mixture of tetrahydrofuran/methanol (150 mL) was added to aid solubility over the course of the 4 day reaction. Work up yielded the title compound (1.15 g, 6.9 mmol, 78%) as a colourless solid; δ_H (500 MHz, MeOD) 7.69 (d, *J* = 8.5 Hz, 1 H, *H*6), 6.34 (d, *J* = 1.9 Hz, 1 H, *H*3), 6.28 (dd, *J* = 8.5, 1.9 Hz, 1 H, *H*5), 3.87 (s, 3 H, *H*α); δ_C (125 MHz, MeOD) 169.4, 162.8, 156.8, 135.6, 107.9, 106.2, 97.3, 56.3; v_{max}/cm^{-1} (solid state) = 3420, 3337, 3226, 1698, 1603, 1333, 1267, 1022, 823; ESI-HRMS found *m/z* 190.0476 $[M+Na]^+$, $C_8H_9NNaO_3$ requires 190.0475.

4-Amino-2-sec-butoxybenzoic acid

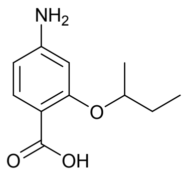

Procedure D; methyl 4-amino-2-sec-butoxybenzoate (750 mg, 3.4 mmol) in a 1:1 mixture of tetrahydrofuran/methanol (100 mL), 10% aqueous sodium hydroxide (30 mL). Further aliquots of 10% aqueous sodium hydroxide were added to bring the reaction to completion. Work up yielded the title compound (700 g, 3.3 mmol, quant.) as an orange oil; δ_H (500 MHz, $CDCl_3$) 10.94 (s, broad, 1 H, CO_2*H*), 7.96 (d, *J* = 8.5 Hz, 1 H, *H*6), 6.36 (dd, *J* = 8.5, 2.1 Hz, 1 H, *H*5), 6.23 (d, *J* = 2.1 Hz, 1 H, *H*3), 4.53 – 4.59 (m, 1 H, *H*α), 4.23 (s, broad, 2 H, NH_2), 1.81 – 1.92 (m, 1 H, *H*β), 1.71 – 1.81 (m, 1 H, *H*β′), 1.41 (d, *J* = 6.2 Hz, 3 H, CHα(C$\underline{H}_3$)), 1.03 (t, *J* = 7.6 Hz, 3 H, Hγ); δ_C (125 MHz, $CDCl_3$) 165.9, 158.5, 152.7, 135.5, 108.6, 108.3, 98.7, 78.5, 29.1, 19.2, 9.6; v_{max}/cm^{-1} (solid state) = 3468, 3358, 3233, 2973, 2936, 2880, 1707, 1601, 1458, 1394, 1268, 988; ESI-HRMS found *m/z* 210.1128 [M+H] $^+$, $C_{11}H_{16}NO_3$ requires 210.1125.

4-(((9H-Fluoren-9-yl)methoxy)carbonylamino)-2-methoxybenzoic acid

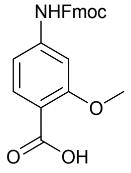

Procedure E; 4-amino-2-methoxybenzoic acid (1.0 g, 6.0 mmol) in tetrahydrofuran (40 mL) and fluorenylmethyloxycarbonyl chloride (2.6 g, 9.0 mmol) in tetrahydrofuran (20 mL). After work up, the reaction mixture was purified by column chromatography (*Stationary Phase*: Silica; *Mobile Phase*: A gradient of dichloromethane to dichloromethane/ethylacetate 1:1 to ethylacetate/methanol 9.5:0.5) to yield the title compound (1.13 g, 2.9 mmol, 48%) as a beige solid; δ_H (500 MHz, $CDCl_3$) 10.60 (s, broad, 1 H, CO_2*H*), 8.09 (d, *J* = 8.5 Hz, 1 H, *H*6), 7.79 (d, *J* = 8.5 Hz, 2 H, F*H*Ar5), 7.65 (s, 1 H, *H*3), 7.64 (d, *J* = 7.5 Hz, 2 H, F*H*Ar2), 7.43 (t, *J* = 7.5 Hz, 2 H, F*H*Ar4), 7.34 (t, *J* = 7.5 Hz, 2 H, F*H*Ar3), 7.16 (s, broad, 1 H, N*H*), 6.81 (dd, *J* = 8.5, 1.5 Hz, 1 H, *H*5), 4.59 (d, *J* = 6.5 Hz, 2 H, F*H*□), 4.28 (t, *J* = 6.5 Hz, 1 H, F*H*α), 4.06 (s, 3 H, *H*β); δ_C (125 MHz, $CDCl_3$) 165.1, 159.2, 152.9, 144.3, 143.4, 141.4, 134.6, 128.0, 127.4, 124.8, 120.2, 112.1, 111.4, 101.1, 67.2, 56.8, 47.0; v_{max}/cm^{-1} (solid state) = 3276, 3018, 2948, 1714, 1594, 1530, 1215, 755, 740; ESI-HRMS found *m/z* 390.1340 $[M+H]^+$, $C_{23}H_{20}NO_5$ requires 390.1336.

4-(((9H-Fluoren-9-yl)methoxy)carbonylamino)-2-sec-butoxybenzoic acid

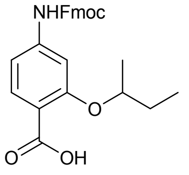

Procedure E; 4-amino-2-sec-butoxybenzoic acid (700 mg, 3.3 mmol) in tetrahydrofuran (50 mL) and fluorenylmethyloxycarbonyl chloride (1.3 g, 5.0 mmol) in tetrahydrofuran (10 mL). Work up yielded the title compound (435 mg, 1.0 mmol, 31%) as an off-white solid; δ_H (500 MHz, $CDCl_3$) 11.06 (s, broad, 1 H, CO_2*H*), 8.10 (d, *J* = 8.5 Hz, 1 H, *H*6), 7.80 (d, *J* = 7.5 Hz, 2 H, F*H*Ar5), 7.68 (s, 1 H, *H*3), 7.63 (d, *J* = 7.5 Hz, 2 H, F*H*Ar2), 7.44 (t, *J* = 7.5 Hz, 2 H, F*H*Ar4), 7.34 (t, *J* = 7.5 Hz, 2 H, F*H*Ar3), 7.02 (s, broad, 1 H, N*H*), 6.76 (dd, *J* = 8.5, 1.5 Hz, 1 H, *H*5), 4.65 – 4.72 (m, 1 H, *H*α), 4.58 (d, *J* = 6.5 Hz, 2 H, F*H*α), 4.29 (t, *J* = 6.5 Hz, 1 H, F*H*β), 1.85 – 1.93 (m, 1 H, *H*β), 1.74 – 1.84 (m, 1 H, *H*β′), 1.45 (d, *J* = 6.2 Hz, 3 H, CHα(C$\underline{H}_3$)), 1.04 (t, *J* = 7.4 Hz, 3 H, Hγ); δ_C (125 MHz, $CDCl_3$) 165.3, 157.8, 152.9, 149.6, 143.9, 143.4, 141.4, 134.6, 128.0, 127.2, 124.8, 120.2, 111.4, 103.2, 79.0, 67.2, 47.0, 29.0, 19.2, 9.6; v_{max}/cm^{-1} (solid state) = 3273, 2972, 2938, 1713, 1592, 1531, 1449, 1216, 736; ESI-HRMS found *m/z* 432.1810 $[M+H]^+$, $C_{26}H_{26}NO_5$ requires 432.1805.

Methyl 3-sec-butoxy-4-nitrobenzoate

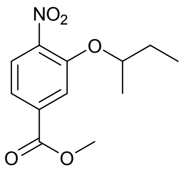

Procedure B; methyl 3-hydroxy-4-nitrobenzoate (2.42 g, 12.3 mmol), *sec*-butanol (1.0 g, 13.5 mmol), triphenylphosphine (4.8 g, 18.4 mmol) in anhydrous tetrahydrofuran (80 mL) and diisopropyl azodicarboxylate (3.61 mL, 18.4 mmol). The reaction mixture was purified by column chromatography (*Stationary Phase*: Silica; *Mobile Phase*: ethyl acetate/hexane, 1:1) to afford the desired product (3.10 g, 12.2 mmol, quant.) as a yellow oil; δ_H (500 MHz, $CDCl_3$) 7.76 (d, *J* = 8.2 Hz, 1 H, *H*5), 7.73 (d, *J* = 1.0 Hz, 1 H, *H*2), 7.66 (dd, *J* = 8.2, 1.0 Hz, 1 H, *H*6), 4.52 – 4.60 (m, 1 H, *H*α), 3.97 (s, 3 H, CO_2C*H*$_3$), 1.85 – 1.76 (m, 1 H, *H*β), 1.67 – 1.76 (m, 1 H, *H*β′), 1.37 (d, *J* = 6.0 Hz, 3 H, CHα(C$\underline{H}_3$)), 1.01 (t, *J* = 7.4 Hz, 3 H, Hγ); δ_C (125 MHz, $CDCl_3$) 165.4, 151.1, 143.7, 134.4, 125.1, 120.9, 116.7, 77.8, 52.8, 29.0, 19.0, 9.5; v_{max}/cm^{-1} (solid state) = 2974, 2937, 2880, 1726, 1605, 1528, 1290, 1235, 744; ESI-HRMS found *m/z* 276.0843 $[M+Na]^+$, $C_{12}H_{15}NNaO_5$ requires 276.0842.

Methyl 4-amino-3-sec-butoxybenzoate

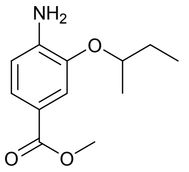

Procedure C; methyl 3-sec-butoxy-4-nitrobenzoate (3.10 g, 12.2 mmol) in a 1:1 mixture of tetrahydrofuran/methanol (40 mL). Work up yielded the title compound (2.72 g, 12.2 mmol, quant.) as a yellow oil; δ_H (500 MHz, $CDCl_3$) 7.52 (dd, *J* = 8.2, 1.5 Hz, 1 H, *H*6), 7.46 (d, *J* = 1.5 Hz, 1 H, *H*2), 6.67 (d, *J* = 8.2 Hz, 1 H, *H*5), 4.39 – 4.45 (m, 1 H, *H*α), 3.86 (s, 3 H, CO_2C*H*$_3$), 1.73 – 1.83 (m, 1 H, *H*β), 1.62 – 1.72 (m, 1 H, *H*β′), 1.32 (d, *J* = 6.2 Hz, 3 H, CHα(C$\underline{H}_3$)), 1.00 (t, *J* = 7.4 Hz, 3 H, Hγ); δ_C (125 MHz, $CDCl_3$) 167.4, 144.4, 142.1, 123.8, 119.5, 113.9, 113.4, 75.8, 51.6, 29.2, 19.3, 9.8; v_{max}/cm^{-1} (solid state) = 3486, 3369, 2971, 2878, 1698, 1614, 1518, 1440, 1290, 1258, 1210, 728; ESI-HRMS found *m/z* 224.1285 $[M+H]^+$, $C_{12}H_{18}NO_3$ requires 224.1281.

4-Amino-3-sec-butoxybenzoic acid

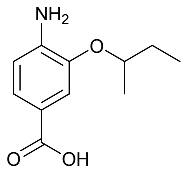

Procedure D; methyl 4-amino-3-sec-butoxybenzoate (2.72 g, 12.2 mmol) in a 1:1 mixture of tetrahydrofuran/methanol (40 mL), 10% aqueous sodium hydroxide (20 mL). Work up yielded the title compound (2.34 g, 11.2 mmol, 92%) as a pale purple solid; δ_H (500 MHz, $CDCl_3$) 7.61 (dd, *J* = 8.2, 1.5 Hz, 1 H, *H*6), 7.51 (d, *J* = 1.5 Hz, 1 H, *H*2), 6.70 (d, *J* = 8.2 Hz, 1 H, *H*5), 4.43 (app sxt, *J* = 6.0 Hz, 1 H, *H*α), 1.75 – 1.85 (m, 1 H, *H*β), 1.62 – 1.74 (m, 1 H, *H*β′), 1.34 (d, *J* = 6.0 Hz, 3 H, CHα(C*H*3)), 1.01 (t, *J* = 7.4 Hz, 3 H, Hγ); δ_C (125 MHz, $CDCl_3$) 172.0, 144.3, 143.0, 124.8, 118.3, 114.2, 113.3, 75.8, 29.2, 19.3, 9.8; v_{max}/cm^{-1} (solid state) = 3494, 3387, 2971, 2936, 2879, 1672, 1612, 1445, 1293, 1265, 1223, 769; ESI-HRMS found *m/z* 210.1122 $[M+H]^+$, $C_{11}H_{16}NO_3$ requires 210.1125.

4-(((9H-Fluoren-9-yl)methoxy)carbonylamino)-3-sec-butoxybenzoic acid

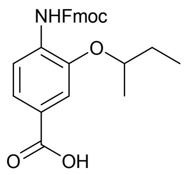

Procedure E; 4-amino-3-sec-butoxybenzoic acid (1.0 g, 4.78 mmol) in tetrahydrofuran (40 mL) and fluorenylmethyloxycarbonyl chloride (1.86 g, 7.17 mmol) in tetrahydrofuran (20 mL). Work up yielded the title compound (1.90 g, 4.40 mmol, 92%) as off-white solid; δ_H (500 MHz, $CDCl_3$) 8.17 (s, broad, 1 H, N*H*), 7.80 (d, *J* = 7.5 Hz, 2 H, F*H*Ar5), 7.73 (d, *J* = 8.0 Hz, 1 H, *H*5), 7.64 (d, *J* = 7.5 Hz, 2 H, F*H*Ar2), 7.59 (s, 1 H, *H*2), 7.56 (s, broad, 1 H, *H*6), 7.44 (t, *J* = 7.5 Hz, 2 H, F*H*Ar4), 7.34 (t, *J* = 7.5 Hz, 2 H, F*H*Ar3), 4.63 – 4.45 (m, 3 H, *H*α, F*H*α), 4.35 (t, *J* = 6.5 Hz, 1 H, F*H*β), 1.91 – 1.80 (m, 1 H, *H*β), 1.80 – 1.70 (m, 1 H, *H*β′), 1.39 (d, *J* = 6.2 Hz, 3 H, CHα(C*H*3)), 1.05 (t, *J* = 7.4 Hz, 3 H, Hγ); δ_C (125 MHz, $CDCl_3$) 169.9, 156.7, 145.5, 143.7, 141.4, 133.6, 127.9, 127.2, 125.0, 124.1, 123.1, 120.1, 117.4, 113.7, 76.9, 67.5, 47.1, 29.1, 19.3, 9.9; v_{max}/cm^{-1} (solid state) = 3431, 2958, 2928, 2874, 1743, 1687, 1594, 1534, 1188, 1026, 738; ESI-HRMS found *m/z* 432.1807 $[M+H]^+$, $C_{26}H_{26}NO_5$ requires 432.1805.

H_2N-[*O*-4-Cl-Bn-(3-HABA)]-Phe-[*O*-*i*Pr-(2-HABA)]-Gly-CO_2H 1a

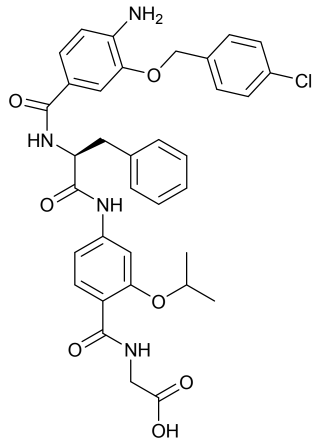

Pale brown solid; isolated yield: 23 mg, 35%; δ_H (500 MHz, MeOD) 7.95 (d, *J* = 8.5 Hz, 1 H, 3-*H*6), 7.60 (d, *J* = 1.7 Hz, 1 H, 3-*H*3), 7.44 (d, *J* = 8.3 Hz, 2 H, 1-*H*Ar2), 7.37 – 7.36 (m, 3 H, 1-*H*Ar3, 1-*H*2), 7.33 – 7.28 (m, 3 H, 2-Phe-*H*Ar2, 1-*H*6), 7.25 – 7.23 (m, 2 H, 2-Phe-*H*Ar3), 7.21 – 7.16 (m, 1 H, 2-Phe-*H*Ar4), 7.07 (dd, *J* = 8.5, 1.7 Hz, 1 H, 3-*H*5), 6.72 (d, *J* = 8.3 Hz, 1 H, 1-*H*5), 5.10 (s, 2 H, 1-*H*α), 4.90 (dd, *J* = 8.3, 6.6 Hz, 1 H, 2-Phe-*H*α), 4.77 (spt, *J* = 6.1, 1 H, 3-*H*α) 4.16 (s, 2 H, 4-Gly-*H*α), 3.27 (dd, *J* = 13.7, 6.6 Hz, 1 H, 2-Phe-*H*β), 3.17 (dd, *J* = 13.7, 8.3 Hz, 1 H, 2-Phe-*H*β′), 1.46 (d, *J* = 6.1 Hz, 3 H, 3-*H*β), 1.44 (d, *J* = 6.1 Hz, 3 H, 3-*H*β′); δ_C (125 MHz, MeOD) 172.9, 170.1, 167.2, 158.3, 146.4, 144.2, 143.1, 138.3, 137.3, 134.7, 133.1, 127.8, 122.9, 117.9, 114.5, 113.0, 112.4, 106.5, 73.6, 70.6, 57.6, 42.6, 38.9, 22.1; v_{max}/cm^{-1} (solid state) = 3357, 2979, 2930, 1683, 1522, 1407, 1211, 698; ESI-HRMS found *m/z* 659.2278 $[M+H]^+$, $C_{35}H_{36}ClN_4O_7$ requires 659.2267.

H_2N-[*O*-4-*t*Bu-Bn-(3-HABA)]-Phe-[*O*-*i*Pr-(2-HABA)]-Gly-CO_2H 2

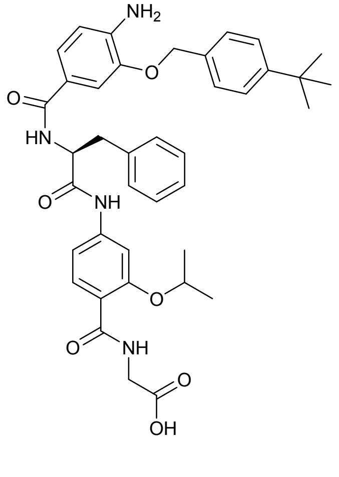

Beige solid; isolated yield: 24 mg, 35%; δ_H (500 MHz, MeOD) 7.95 (d, *J* = 8.7 Hz, 1 H, 3-*H*6), 7.59 (d, *J* = 1.8 Hz, 1 H, 3-*H*3), 7.43– 7.38 (m, 3 H, 1-*H*2, 1-*H*Ar2), 7.38 – 7.34 (m, 2 H, 1-*H*Ar3), 7.33 – 7.28 (m, 3 H, 2-Phe-*H*Ar2, 1-*H*6), 7.25 – 7.23 (m, 2 H, 2-Phe-*H*Ar3), 7.21 – 7.16 (m, 1 H, 2-Phe-*H*Ar4), 7.08 (dd, *J* = 8.7, 1.8 Hz, 1 H, 3-*H*5), 6.71 (d, *J* = 8.1 Hz, 1 H, 1-*H*5), 5.05 (s, 2 H, 1-*H*α), 4.92 (dd, *J* = 8.2, 6.9 Hz, 1 H, 2-Phe-*H*α), 4.74 (spt, *J* = 6.1, 1 H, 3-*H*α) 4.16 (s, 2 H, 4-Gly-*H*α), 3.27 (dd, *J* = 13.8, 6.9 Hz, 1 H, 2-Phe-*H*β), 3.17 (dd, *J* = 13.8, 8.3 Hz, 1 H, 2-Phe-*H*β′), 1.42 (d, *J* = 6.1 Hz, 3 H, 3-*H*β), 1.44 (d, *J* = 6.1 Hz, 3 H, 3-*H*β), 1.31 (s, 9 H, 1-HAr4-C($C\underline{H}_3)_3$); δ_C (125 MHz, MeOD) 173.0, 172.6, 170.2, 167.2, 158.3, 152.1, 146.7, 144.2, 143.0, 138.3, 135.4, 133.1, 130.3, 129.4, 128.6, 127.8, 126.4, 123.0, 122.7, 117.9, 114.4, 113.0, 112.2, 106.5, 73.6, 71.3, 57.6, 42.6, 39.0, 35.3, 31.7, 22.1; ν_{max}/cm^{-1} (solid state) = 3356, 2962, 2870, 1596, 1493, 1408, 1213, 1004, 699; ESI-HRMS found *m/z* 703.3104 $[M+Na]^+$, $C_{39}H_{44}N_4NaO_7$ requires 703.3102.

H_2N-[*O*-4-CF_3-Bn-(3-HABA)]-Phe-[*O*-*i*Pr-(2-HABA)]-Gly-CO_2H 3

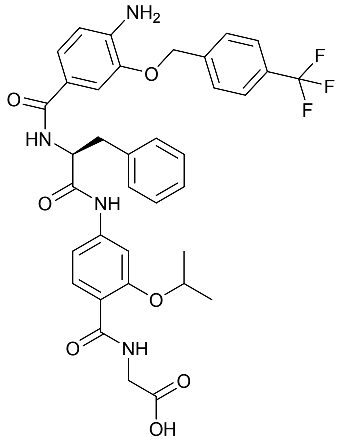

Beige solid; isolated yield: 29 mg, 42%; δ_H (500 MHz, DMSO-d_6) 10.42 (s. broad, 1 H, 4-Gly-CO_2*H*), 8.46 (t, *J* = 4.9 Hz, 1 H, 4-Gly-N*H*), 8.32 (d, *J* = 8.0 Hz, 1 H, 2-Phe-N*H*), 7.89 (d, *J* = 8.5 Hz, 1 H, 3-*H*6), 7.88 – 7.81 (m, 2 H, 1-*H*Ar2), 7.70 – 7.64 (m, 1 H, 1-*H*Ar3), 7.61 (d, *J* = 1.9 Hz, 1 H, 3-*H*3), 7.42 (d, *J* = 1.8 Hz, 1 H, 1-*H*2), 7.38 (d, *J* = 7.3 Hz, 2 H, 2-Phe-*H*Ar2), 7.33 (dd, *J* = 8.5, 1.9 Hz, 1 H, 3-*H*5), 7.28 – 7.22 (m, 3 H, 1-*H*6, 2-Phe-*H*Ar3), 7.17 (t, *J* = 8.5 Hz, 1 H, 2-Phe-*H*Ar4), 6.65 (d, *J* = 8.2 Hz, 1 H, 1-*H*5), 5.40 (s. broad, 2 H, 1-NH_2), 5.24 (s, 2 H, 1-*H*α), 4.84 – 4.76 (m, 2 H, 2-Phe-*H*α), 4.71 (quin, *J* = 5.9 Hz, 1 H, 3-*H*α), 4.04 (d, *J* = 5.0 Hz, 2 H, 4-Gly-*H*α), 3.16 – 3.04 (m, 2 H, 2-Phe-*H*β), 1.41 (dd, *J* = 8.5, 6.5 Hz, 6 H, 3-*H*β); δ_C (125 MHz, DMSO-d_6) 171.4, 171.2, 166.3, 163.9, 156.2, 143.9, 143.0, 141.7, 138.8, 138.1, 131.9, 131.4, 129.4, 129.2, 128.0, 126.3, 123.9, 122.0, 120.8, 116.6, 112.4, 111.6, 111.3, 104.7, 71.9, 68.7, 55.8, 41.6, 37.1, 21.6, 21.6; ν_{max}/cm^{-1} (solid state) = 3340, 2979, 2923, 1615, 1595, 1497, 1213, 1122, 699; ESI-HRMS found *m/z* 693.2549 $[M+H]^+$, $C_{36}H_{36}F_3N_4O_7$ requires 693.2531.

H_2N-[*O*-2-Nph-(3-HABA)]-Phe-[*O*-*i*Pr-(2-HABA)]-Gly-CO_2H 4

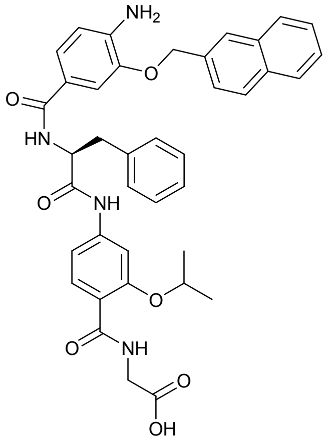

Pale brown; isolated yield: 11 mg, 16%; δ_H (500 MHz, MeOD) 7.95 (d, *J* = 8.6 Hz, 1 H, 3-*H*6), 7.92 – 7.81 (m, 4 H, 1-*H*Ar4, 1-*H*Ar5, 1-*H*Ar8, 1-*H*Ar7), 7.59 – 7.54 (m, 2 H, 1-*H*Ar6, 3-*H*3), 7.49 - 7.42 (m, 3 H, 1-*H*Ar1, 1-*H*Ar3, 1-*H*2), 7.33 – 7.26 (m, 3 H, 2-Phe-*H*Ar2, 1-*H*6), 7.24 – 7.21 (m, 2 H, 2-Phe-*H*Ar3), 7.19 – 7.14 (m, 1 H, 2-Phe-*H*Ar4), 7.07 (dd, *J* = 8.6, 1.8 Hz, 1 H, 3-*H*5), 6.73 (d, *J* = 8.3 Hz, 1 H, 1-*H*5), 5.27 (s, 2 H, 1-*H*α), 4.91 (dd, *J* = 8.3, 6.6 Hz, 1 H, 2-Phe-*H*α), 4.74 (spt, *J* = 6.1, 1 H, 3-*H*α), 4.15 (s, 2 H, 4-Gly-*H*α), 3.26 (dd, *J* = 13.8, 6.6 *H*z, 1 H, 2-Phe-*H*β), 3.17 (dd, *J* = 13.8, 8.3 Hz, 1 H, 2-Phe-*H*β′), 1.42 (d, *J* = 6.1 Hz, 3 H, 3-*H*β), 1.43 (d, *J* = 6.1 Hz, 3 H, 3-*H*β′); δ_C (125 MHz, MeOD) 172.9, 170.1, 167.2, 158.3, 146.7, 144.2, 143.6, 143.1, 138.3, 135.9, 134.7, 134.5, 133.1, 130.3, 129.4, 129.2, 128.9, 128.6, 127.8, 127.4, 127.2, 127.0, 126.4, 123.0, 122.9, 117.9, 114.5, 113.0, 112.5, 106.5, 73.6, 71.5, 57.6, 42.7, 39.0, 22.1; v_{max}/cm^{-1} (solid state) = 3357, 3276, 2979, 2929, 2872, 1594, 1507, 1496, 1213; ESI-HRMS found *m/z* 675.2827 $[M+H]^+$, $C_{39}H_{39}N_4O_7$ requires 675.2813.

H_2N-[*O*-1-Nph-(3-HABA)]-Phe-[*O*-*i*Pr-(2-HABA)]-Gly-CO_2H 5

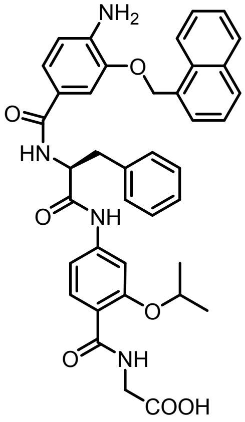

δ_H (500 MHz, MeOD) 8.01 (d, *J* = 8.6 Hz, 1 H, 3-*H*6), 7.92– 7.83 (m, 3 H, 1-*H*Ar4, 1-*H*Ar5, 1-*H*Ar7), 7.55 – 7.52 (m, 3 H, 1-*H*Ar6, 1-*H*Ar8, 3-*H*3), 7.49 - 7.41 (m, 3 H, 1-*H*Ar2, 1-*H*Ar3, 1-*H*2), 7.33 – 7.31 (m, 3 H, 2-Phe-*H*Ar2, 1-*H*6), 7.24 – 7.21 (m, 2 H, 2-Phe-*H*Ar3), 7.18 – 7.15 (m, 1 H, 2-Phe-*H*Ar4), 7.09 (dd, *J* = 8.7, 1.8 Hz, 1 H, 3-*H*5), 6.68 (d, *J* = 8.3 Hz, 1 H, 1-*H*5), 5.44 (s, 2 H, 1-*H*α), 4.99 (dd, *J* = 8.4, 6.7 Hz, 1 H, 2-Phe-*H*α), 4.65 (spt, *J* = 6.1, 1 H, 3-*H*α), 4.14 (s, 2 H, 4-Gly-*H*α), 3.28 (dd, *J* = 13.8, 8.5 *H*z, 1 H, 2-Phe-*H*β), 3.19 (dd, *J* = 13.8, 8.5 Hz, 1 H, 2-Phe-*H*β′), 1.37 (d, *J* = 6.1 Hz, 3 H, 3-*H*β), 1.39 (d, *J* = 6.1 Hz, 3 H, 3-*H*β′). ESI-HRMS found *m/z* 675.2812 $[M+H]^+$, $C_{39}H_{39}N_4O_7$ requires 675.2813.

H_2N-[*O*-*s*Bu-(3-HABA)]-Phe-[*O*-*i*Pr-(2-HABA)]-Gly-CO_2H 6

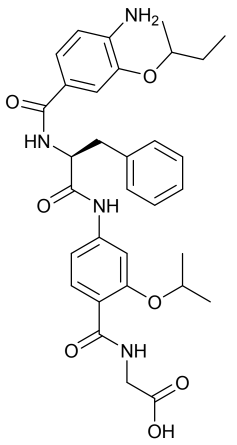

Beige solid; isolated yield: 22 mg, 37%; δ_H (500 MHz, MeOD) 7.95 (d, *J* = 8.5 Hz, 1 H, 3-*H*6), 7.60 (d, *J* = 1.8 Hz, 1 H, 3-*H*3), 7.34 – 7.28 (m, 3 H, 2-Phe-*H*Ar2, 1-*H*2), 7.28 – 7.23 (m, 3 H, 2-Phe-*H*Ar3, 1-*H*6), 7.21 – 7.16 (m, 1 H, 2-Phe-*H*Ar4), 7.07 (dd, *J* = 8.5, 1.8 Hz, 1 H, 3-*H*5), 6.71 (d, *J* = 8.3 Hz, 1 H, 1-*H*5), 4.90 (dd, *J* = 8.2, 6.8 Hz, 1 H, 2-Phe-*H*α), 4.77 (spt, *J* = 6.1 Hz, 1 H, 3-*H*α), 4.41 – 4.37 (m, 1 H, 1-*H*α), 4.16 (s, 2 H, 4-Gly-*H*α), 3.27 (dd, *J* = 13.5, 6.8 Hz, 1 H, 2-Phe-*H*β), 3.17 (dd, *J* = 13.5, 8.2 Hz, 1 H, 2-Phe-*H*β′), 1.80 – 1.71 (m, 1 H, 1-*H*β), 1.69 – 1.62 (m, 1 H, 1-*H*β′), 1.45 (d, *J* = 6.1 Hz, 3 H, 3-*H*β), 1.44 (d, *J* = 6.1 Hz, 3 H, 3-*H*β′), 1.28 (dd, *J* = 6.0, 4.3 Hz, 3 H, 1-CHα(C$\underline{H}_3$)), 1.00 – 0.91 (m, 3 H, 1-*H*γ); δ_C (125 MHz, MeOD) 172.9, 172.6, 170.3, 167.2, 158.3, 145.8, 144.2, 138.3, 133.1, 130.3, 129.4, 127.8, 123.1, 122.3, 117.9, 114.6, 113.6, 113.6, 113.0, 106.5, 77.0, 73.6, 57.6, 42.5, 38.9, 30.2, 22.1, 19.6, 10.0; ν_{max}/cm^{-1} (solid state) = 3350, 3034, 2973, 2929, 1670, 1497, 1215, 699; ESI-HRMS found *m/z* 591.2824 $[M+H]^+$, $C_{32}H_{39}N_4O_7$ requires 591.2813.

H_2N-[*O*-CH_2-CH_2-CH_2-S-CH_2-(3-HABA)]-Phe-[*O*-*i*Pr-(2-HABA)]-Gly-CO_2H 7

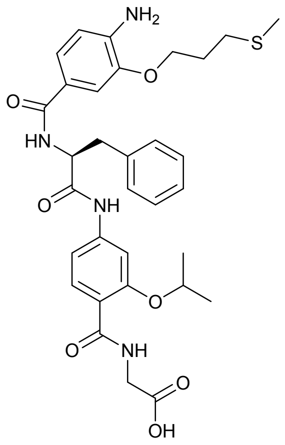

Dark yellow solid; isolated yield: 60 mg, 96%; δ_H (500 MHz, MeOD) 7.95 (d, *J* = 8.5 Hz, 1 H, 3-*H*6), 7.60 (d, *J* = 1.8 Hz, 1 H, 3-*H*3), 7.33 – 7.28 (m, 3 H, 2-Phe-*H*Ar2, 1-*H*2), 7.28 – 7.23 (m, 3 H, 2-Phe-*H*Ar3, 1-*H*6), 7.21 – 7.16 (m, 1 H, 2-Phe-*H*Ar4), 7.06 (dd, *J* = 8.5, 1.8 Hz, 1 H, 3-*H*5), 6.71 (d, *J* = 7.9 Hz, 1 H, 1-*H*5), 4.90 (dd, *J* = 8.3, 6.8 Hz, 1 H, 2-Phe-*H*α), 4.77 (spt, *J* = 6.1, 1 H, 3-*H*α), 4.16 (s, 2 H, 4-Gly-*H*α), 4.12 (t, *J* = 6.0 Hz, 2 H, 1-*H*α), 3.27 (dd, *J* = 13.5, 6.8 Hz, 1 H, 2-Phe-*H*β), 3.17 (dd, *J* = 13.5, 8.3 Hz, 1 H, 2-Phe-*H*β′), 2.69 (t, *J* = 7.2 Hz, 2 H, 1-*H*α), 2.09 (m, 5 H, 1-Hβ, 1-SC$\underline{H}_3$), 1.45 (d, *J* = 6.1 Hz, 3 H, 3-*H*β), 1.44 (d, *J* = 6.1 Hz, 3 H, 3-*H*β′); δ_C (125 MHz, MeOD) 172.9, 170.2, 167.2, 158.3, 152.3, 146.9, 144.2, 138.3, 133.1, 130.3, 129.4, 127.8, 123.3, 122.6, 117.9, 116.2, 114.5, 113.0, 111.8, 106.5, 73.6, 67.9, 57.6, 42.5, 38.9, 31.6, 29.8, 22.1, 15.3; ν_{max}/cm^{-1} (solid state) = 3357, 2974, 2918, 1595, 1508, 1497, 1212; ESI-HRMS found *m/z* 623.2540 $[M+H]^+$, $C_{32}H_{39}N_4O_7S$ requires 623.2534.

H_2N-[*O*-4-Cl-Bn-(3-HABA)]-Phe-[*O*-*i*Pr-(2-HABA)]-Gly-CO_2H 8

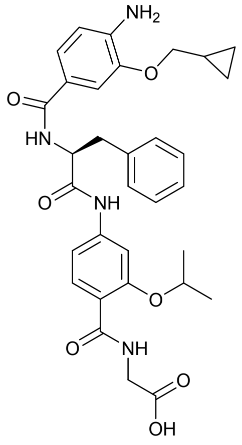

Off-white solid, > 95% pure by NMR; isolated yield: 55 mg, 93%; δ_H (500 MHz, DMSO-d_6) 10.47 (s. broad, 1 H, 4-Gly-CO_2*H*), 8.63 (s. broad, 1 H, 4-Gly-N*H*), 8.41 (d, *J* = 8.1 Hz, 1 H, 2-Phe-N*H*), 7.90 (d, *J* = 8.5 Hz, 1 H, 3-*H*6), 7.57 (s, 1 H, 3-*H*3), 7.39 (m, 3 H, 1-*H*2, 2-Phe-*H*Ar2), 7.32 – 7.22 (m, 4 H, 3-*H*5, 1-*H*6, 2-Phe-*H*Ar3), 7.17 (t, *J* = 8.5 Hz, 1 H, 2-Phe-*H*Ar4), 6.61 (d, *J* = 8.5 Hz, 1 H, 1-*H*5), 5.20 (s. broad, 2 H, 1-NH_2), 4.84 – 4.76 (m, 2 H, 2-Phe-*H*α), 4.68 (quin, *J* = 5.9 Hz, 1 H, 3-*H*α), 3.83 (d, *J* = 6.8 Hz, 2 H, 4-Gly-*H*α), 3.16 – 3.09 (m, 2 H, 2-Phe-*H*β), 1.40 (dd, *J* = 8.5, 6.5 Hz, 6 H, 3-*H*β), 0.91 – 0.82 (m, 1 H, 1-*H*α), 0.60 – 0.52 (m, 2 H, 1-*H*β), 0.37 – 0.32 (m, 2 H, 1-*H*β′); δ_C (125 MHz, DMSO-d_6) 171.4, 166.4, 163.0, 156.2, 144.4, 142.7, 141.4, 141.0, 138.3, 131.8, 129.2, 128.0, 126.3, 121.6, 121.0, 117.2, 112.2, 111.3, 111.1, 104.6, 72.5, 71.6, 56.0, 37.1, 29.0, 21.6, 10.3, 3.1; ν_{max}/cm^{-1} (solid state) = 3324, 3090, 2926, 2852, 1595, 1509, 1496, 1406, 1105, 1007, 919; ESI-HRMS found *m/z* 589.2669 $[M+H]^+$, $C_{32}H_{37}N_4O_7$ requires 589.2657.

H_2N-[*O*-Bn-(3-HABA)]-Phe-[*O*-*i*Pr-(2-HABA)]-Lys-CO_2H 9

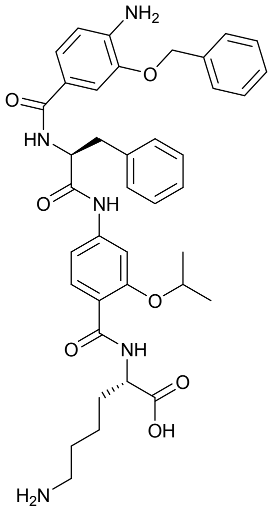

Brown solid; isolated yield: 41 mg, 59%; δ_H (500 MHz, MeOD) 7.94 (d, *J* = 8.5 Hz, 1 H, 3-*H*6), 7.55 (d, *J* = 1.8 Hz, 1 H, 3-*H*3), 7.45 – 7.44 (m, 2 H, 2-Phe-*H*Ar2), 7.41 (d, *J* = 1.7 Hz, 1 H, 1-*H*2), 7.37 – 7.33 (m, 2 H, 1-*H*Ar2), 7.33 – 7.24 (m, 4 H, 1-*H*Ar3, 1-*H*Ar4, 1-*H*6), 7.27 – 7.24 (m, 2 H, 2-Phe-*H*Ar3), 7.21 – 7.18 (m, 1 H, 2-Phe-*H*Ar4), 7.12 (dd, *J* = 8.5, 1.8 Hz, 1 H, 3-*H*5), 6.76 (d, *J* = 8.3 Hz, 1 H, 1-*H*5), 5.11 (d, *J* = 1.5 Hz, 2 H, 1-*H*α), 4.90 (dd, *J* = 8.1, 7.1 Hz, 1 H, 2-Phe-*H*α), 4.80 – 4.74 (m, 1 H, 3-*H*α), 4.69 (dd, *J*=6.8, 5.6 Hz, 1 H, 4-Lys-*H*α), 3.29 – 3.23 (dd, 2 H, 2-Phe-*H*β), 2.92 (t, *J* = 7.0 Hz, 2 H, 4-Lys-*H*ε), 2.11 – 1.81 (m, 2 H, 4-Lys-*H*β), 1.79 – 1.64 (m, 2 H, 4-Lys-*H*δ), 1.58 – 1.48 (m, 2 H, 4-Lys-*H*γ), 1.47 (d, *J* = 6.2 Hz, 3 H, 3-*H*β) 1.40 (d, *J* = 6.0 Hz, 3 H, 3-*H*β′); δ_C (125 MHz, MeOD) 174.7, 173.0, 170.1, 166.8, 158.2, 147.1, 144.3, 141.8, 138.3, 133.1, 130.3, 129.6, 129.5, 129.4, 129.0, 128.7, 127.8, 123.9, 122.7, 117.8, 115.1, 113.0, 112.4, 106.3, 73.5, 71.5, 57.7, 53.5, 40.4, 38.9, 33.2, 28.1, 23.3, 22.3, 22.0; ν_{max}/cm^{-1} (solid state) = 3032, 2932, 1670, 1626, 1600, 1495, 1198, 1182, 1132, 698, 681; ESI-HRMS found *m/z* 696.3410 $[M+H]^+$, $C_{39}H_{46}N_5O_7$ requires 696.3392.

H_2N-[*O*-Bn-(3-HABA)]-Phe-[*O*-*i*Bu-(2-HABA)]-Gly-CO_2H 10

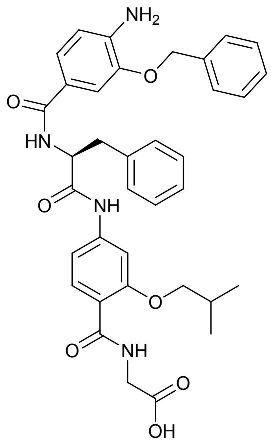

Brown solid; isolated yield: 41 mg, 63%; δ_H (500 MHz, MeOD) 7.90 (d, *J* = 8.6 Hz, 1 H, 3-*H*6), 7.47 (d, *J* = 1.7 Hz, 1 H, 3-*H*3), 7.38 (d, *J* = 1.9 Hz, 1 H, 1-*H*2), 7.37 - 7.33 (m, 2 H, 1-*H*Ar2), 7.33 - 7.24 (m, 6 H, 1-*H*Ar3, 1-*H*Ar4, 2-Phe-*H*Ar2, 1-*H*6), 7.18 - 7.24 (m, 2 H, 2-Phe-*H*Ar3), 7.14 - 7.18 (m, 1 H, 2-Phe-*H*Ar4), 7.09 (dd, *J* = 8.6, 1.7 Hz, 1 H, 3-*H*5), 6.67 (d, *J* = 8.3 Hz, 1 H, 1-*H*5), 5.00 (dd, *J* = 8.2, 6.8 Hz, 1 H, 2-Phe-*H*α), 4.96 (d, *J* = 3.2 Hz, 2 H, 1-*H*α), 4.15 (s, 2 H, 4-Gly-*H*α), 3.68 - 3.80 (m, 2 H, 3-*H*α), 3.13 - 3.29 (dd, *J* = 8.2, 6.8 Hz, 2 H, 2-Phe-*H*β), 2.17 (m, 1 H, 3-*H*β), 0.97 (dd, *J* = 4.5, 6.8 Hz, 6 H, 3-*H*γ); δ_C (125 MHz, MeOD) 173.3, 172.7, 170.0, 167.2, 159.4, 146.6, 144.3, 143.0, 138.4, 133.1, 130.3, 130.3, 129.5, 129.4, 128.9, 128.6, 127.8, 122.9, 122.8, 117.0, 114.4, 112.8, 112.3, 104.9, 76.8, 71.3, 57.8, 42.6, 38.9, 29.0, 19.6; ν_{max}/cm^{-1} (solid state) = 3370, 2956, 2924, 2873, 1600, 1498, 1394, 1215, 1016, 697; ESI-HRMS found *m/z* 639.2830 $[M+H]^+$, $C_{36}H_{39}N_4O_7$ requires 639.2813.

H_2N-[*O*-Bn-(3-HABA)]-Phe-[*O*-*s*Bu-(2-HABA)]-Gly-CO_2H 11

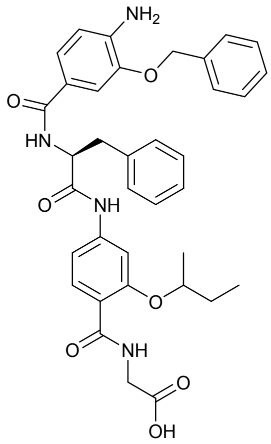

Brown solid; isolated yield: 15 mg, 23%; δ_H (500 MHz, MeOD) 7.94 (d, *J* = 8.5 Hz, 1 H, 3-*H*6), 7.53 (d, *J* = 1.6 Hz, 1 H, 3-*H*3), 7.40 – 7.39 (m, 3 H, 1-*H*2, 1-*H*Ar2), 7.36 – 7.25 (m, 6 H, 1-*H*Ar3, 1-*H*Ar4, 2-Phe-*H*Ar2, 1-*H*6), 7.23 (t, *J* = 7.4 Hz, 2 H, 2-Phe-*H*Ar3), 7.20 – 7.14 (m, 1 H, 2-Phe-*H*Ar4), 7.10 (dd, *J* = 8.5, 1.6 Hz, 1 H, 3-*H*5), 6.73 (d, *J* = 8.3 Hz, 1 H, 1-*H*5), 5.02 (s, 2 H, 1-*H*α), 4.99 – 4.96 (m, 1 H, 2-Phe-*H*α), 4.50 – 4.41 (m, 1 H, 3-*H*α), 4.15 (s, 2 H, 4-Gly-*H*α), 3.28 – 3.16 (m, 2 H, 2-Phe-*H*β), 1.91 – 1.83 (m, 1 H, 3-*H*β), 1.73 – 1.65 (m, 1 H, 3-*H*β′), 1.34 (dd, *J* = 11.2, 6.1 Hz, 3 H, 3-CHα($C\underline{H}_3$)), 1.00 – 0.91 (m, 3 H, 3-*H*γ); δ_C (125 MHz, MeOD) 173.1, 172.5, 170.0, 167.2, 158.5, 147.0, 144.2, 141.8, 138.3, 138.3, 133.2, 130.3, 129.5, 128.9, 128.7, 127.8, 123.7, 122.8, 117.7, 115.0, 112.9, 112.3, 106.3, 78.6, 71.2, 57.7, 42.5, 38.9, 29.8, 19.5, 10.1; ν_{max}/cm^{-1} (solid state) = 3334, 2966, 2924, 2853, 1696, 1495, 1219, 1201, 650; ESI-HRMS found *m/z* 639.2826 $[M+H]^+$, $C_{36}H_{39}N_4O_7$ requires 639.2813.

H_2N-[*O*-Bn-(3-HABA)]-Phe-[*O*-Me-(2-HABA)]-Gly-CO_2H 12

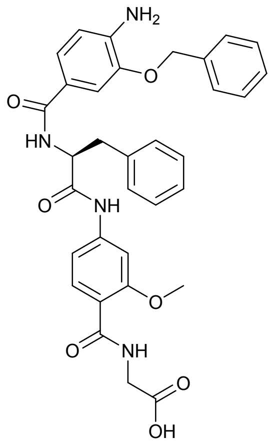

Brown solid; isolated yield: 30 mg, 50%; δ_H (500 MHz, MeOD) 7.93 (d, *J* = 8.5 Hz, 1 H, 3-*H*6), 7.58 (d, *J* = 1.8 Hz, 1 H, 3-*H*3), 7.47 – 7.45 (m, 2 H, 1-*H*Ar2), 7.41 – 7.34 (m, 3 H, 1-*H*2, 1-*H*Ar3), 7.34 – 7.28 (m, 4 H, 1-*H*Ar4, 2-Phe-*H*Ar2, 1-*H*6), 7.27 – 7.24 (m, 2 H, 2-Phe-*H*Ar3), 7.22 – 7.17 (m, 1 H, 2-Phe-*H*Ar4), 7.09 (dd, *J* = 8.6, 1.7 Hz, 1 H, 3-*H*5), 6.72 (d, *J* = 7.9 Hz, 1 H, 1-*H*5), 5.12 (s, 2 H, 1-*H*α), 4.91 (dd, *J* = 8.1, 6.9 Hz, 1 H, 2-Phe-*H*α), 4.11 (s, 2 H, 4-Gly-*H*α), 3.97 (s, 3 H, 3-*H*α), 3.27 (dd, *J* = 13.5, 6.8 Hz, 1 H, 2-Phe-*H*β), 3.17 (dd, *J* = 13.5, 8.3 Hz, 1 H, 2-Phe-*H*β′); δ_C (125 MHz, MeOD) 172.9, 170.2, 169.6, 167.2, 160.0, 146.7, 144.3, 143.0, 138.4, 138.3, 133.0, 130.3, 129.5, 129.4, 128.9, 128.7, 127.8, 123.0, 122.8, 117.3, 114.4, 112.9, 112.3, 104.2, 79.4, 71.4, 57.6, 56.5, 38.9; v_{max}/cm^{-1} (solid state) = 3370, 3029, 2924, 2852, 1601, 1498, 1452, 1405, 1217, 1026, 698; ESI-HRMS found *m/z* 597.2355 $[M+H]^+$, $C_{33}H_{33}N_4O_7$ requires 597.2344.

H_2N-[*O*-Bn-(3-HABA)]-*N*-Me-Phe-[*O*-*i*Pr-(2-HABA)]-Gly-CO_2H 13

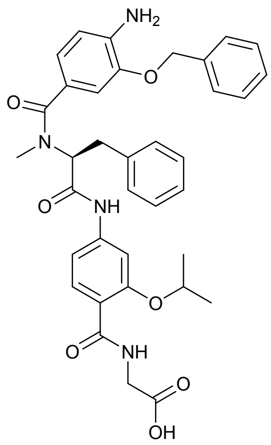

Beige solid; isolated yield: 15 mg, 23%; δ_H (500 MHz, DMSO-d_6) 10.28 (s. broad, 1 H, 4-Gly-CO_2*H*), 8.48 (t, *J* = 4.8 Hz, 1 H, 4-Gly-N*H*), 7.91 (d, *J* = 8.5 Hz, 1 H, 3-*H*6), 7.64 (s, 1 H, 3-*H*3), 7.30 – 7.20 (m, 11 H, 1-*H*Ar2, 1-*H*Ar3, 2-Phe-*H*Ar3, 2-Phe-*H*Ar2, 3-*H*5, 2-Phe-*H*Ar4, 1-*H*Ar4), 6.61 – 6.50 (m, 3 H, 1-*H*5, 1-*H*6, 1-*H*2), 5.15 (s. broad, 2 H, 1-NH_2), 4.99 – 4.83 (m, 2 H, 1-*H*α), 4.71 (spt, *J* = 5.9 Hz, 1 H, 3-*H*α), 4.00 (d, *J* = 4.7 Hz, 2 H, 4-Gly-*H*α), 3.29 (d, *J* = 12.2 Hz, 2 H, 2-Phe-*H*α), 3.12 (dd, *J* = 14.5, 10.9 Hz, 2 H, 2-Phe-*H*β), 2.88 (s, 3 H, 2-NCH_3), 1.39 (d, *J* = 6.0 Hz, 6 H, 3-*H*β); δ_C (125 MHz, DMSO-d_6) 171.9, 171.2, 170.1, 163.8, 156.2, 143.9, 142.8, 140.0, 137.6, 137.0, 131.9, 128.7, 128.3, 128.3, 127.6, 127.1, 126.4, 122.7, 121.4, 116.9, 112.7, 111.6, 105.0, 71.9, 69.3, 42.0, 34.9, 34.6, 34.5, 21.6; v_{max}/cm^{-1} (solid state) = 3366, 3033, 2979, 2930, 1604, 1454, 1389, 1235, 699; ESI-HRMS found *m/z* 639.2820 $[M+H]^+$, $C_{36}H_{39}N_4O_7$ requires 639.2813.

H_2N-[*O*-4-*t*Bu-Bn-(3-HABA)]-D-Phe-[*O*-*i*Pr-(2-HABA)]-Gly-CO_2H 14

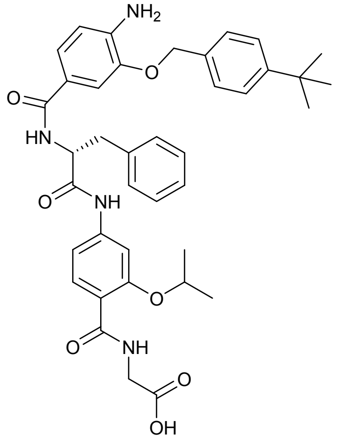

Beige solid; isolated yield: 34 mg, 50%; δ_H (500 MHz, MeOD) 7.94 (d, *J* = 8.6 Hz, 1 H, 3-*H*6), 7.58 (d, *J* = 1.9 Hz, 1 H, 3-*H*3), 7.42 – 7.38 (m, 3 H, 1-*H*2, 1-*H*Ar2), 7.37 – 7.33 (m, 2 H, 1-*H*Ar3), 7.32 – 7.29 (m, 3 H, 2-Phe-*H*Ar2, 1-*H*6), 7.25 – 7.23 (m, 2 H, 2-Phe-*H*Ar3), 7.19 – 7.17 (m, 1 H, 2-Phe-*H*Ar4), 7.08 (dd, *J* = 8.6, 1.9 Hz, 1 H, 3-*H*5), 6.70 (d, *J* = 8.2 Hz, 1 H, 1-*H*5), 5.03 (s, 2 H, 1-*H*α), 4.94 (dd, *J* = 8.2, 7.0 Hz, 1 H, 2-Phe-*H*α), 4.74 (spt, *J* = 6.3 Hz, 1 H, 3-*H*α), 4.15 (s, 2 H, 4-Gly-*H*α), 3.27 (dd, *J* = 13.9, 7.0 Hz, 1 H, 2-Phe-*H*β), 3.17 (dd, *J* = 13.9, 8.2 Hz, 1 H, 2-Phe-*H*β′), 1.41 (d, *J* = 6.3 Hz, 3 H, 3-*H*β), 1.42 (d, *J* = 6.3 Hz, 3 H, 3-*H*β′), 1.30 (s, 9 H, 1-HAr4-C($C\underline{H}_3$)$_3$); δ_C (125 MHz, MeOD) 173.0, 171.4, 170.2, 167.2, 158.3, 152.1, 146.7, 144.2, 143.0, 138.3, 135.4, 133.1, 130.3, 129.4, 128.6, 127.8, 126.4, 122.9, 122.8, 117.9, 114.4, 113.0, 112.2, 106.5□□73.6, 71.3, 57.6, 42.6, 39.0, 35.3, 31.7, 22.1; ν_{max}/cm^{-1} (solid state) = 3362, 2960, 2929, 2868, 1651, 1496, 1445, 1404, 1218, 818; ESI-HRMS found *m/z* 681.3297 $[M+H]^+$, $C_{39}H_{45}N_4O_7$ requires 681.3283.

3-H_2N-Pr-HN-[*O*-Bn-(3-HABA)]-Phe-[*O*-*i*Pr-(2-HABA)]-Gly-CO_2H 15

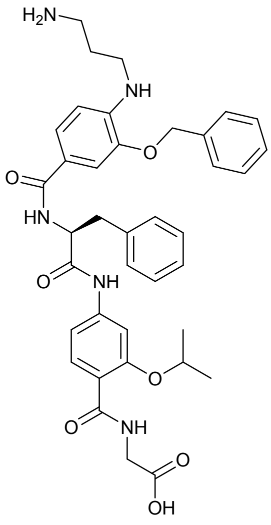

Off-white solid; isolated yield: 26 mg, 38%; δ_H (500 MHz, MeOD) 7.92 (d, *J* = 8.6 Hz, 1 H, 3-*H*6), 7.48 (d, *J* = 1.7 Hz, 1 H, 3-*H*3), 7.40 (dd, *J* = 8.4, 1.7 Hz, 1 H, 1-*H*6), 7.36 (d, *J* = 1.7 Hz, 1 H, 1-*H*2), 7.35 – 7.25 (m, 7 H, 2-Phe-*H*Ar2, 1-*H*Ar2, 1-*H*Ar3, 1-*H*Ar4), 7.24 – 7.21 (m, 2 H, 2-Phe-*H*Ar3), 7.18 – 7.15 (m, 1 H, 2-Phe-*H*Ar4), 7.10 (dd, *J* = 8.6, 1.7 Hz, 1 H, 3-*H*5), 6.54 (d, *J* = 8.4 Hz, 1 H, 1-*H*5), 5.06 (dd, *J* = 8.5, 7.0 Hz, 1 H, 2-Phe-*H*α), 4.94 (d, *J* = 4.5 Hz, 2 H, 1-*H*α), 4.58 (sept, *J* = 6.1 Hz, 1 H, 3-*H*α), 3.95 (s, 2 H, 4-Gly-*H*α), 3.29 – 3.25 (m, 3 H, 2-Phe-*H*β, 1-$NHC\underline{H}_2$), 3.23 (dd, *J* = 7.5, 13.5 Hz, 1 H, 2-Phe-*H*β′), 2.96 (t, *J* = 7.5, 2 H, 1-$NHCH_2CH_2C\underline{H}_2$), 1.95 – 1.86 (m, 2 H, 1-$NHCH_2C\underline{H}_2$), 1.35 (d, *J* = 10.9 Hz, 3 H, 3-*H*β), 1.34 (d, *J* = 10.6 Hz, 3 H, 3-*H*β′); ν_{max}/cm^{-1} (solid state) = 3355, 3033, 2979, 2928, 1594, 1509, 1491, 1256, 1211, 1105, 697; ESI-HRMS found *m/z* 341.6654 $[M+2H]^{2+}$, $C_{38}H_{45}N_5O_7$ requires 341.6654.

H_2N-[*O*-4-*t*Bu-Bn-(3-HABA)]-*p*-Br-Phe-[*O*-*i*Pr-(2-HABA)]-Gly-CO_2H 16

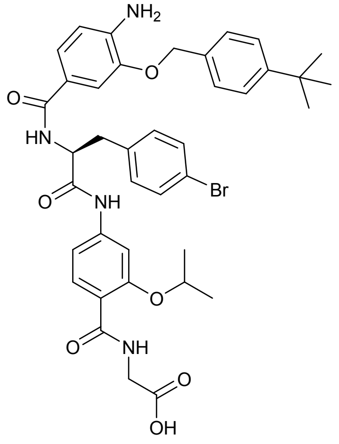

Beige solid; isolated yield: 23 mg, 30%; δ_H (500 MHz, DMSO-d_6) 10.43 (s. broad, 1 H, 4-Gly-CO_2*H*), 8.46 (t, *J* = 4.9 Hz, 1 H, 4-Gly-N*H*), 8.34 (d, *J* = 8.1 Hz, 1 H, 2-Phe-N*H*), 7.90 (d, *J* = 8.8 Hz, 1 H, 3-*H*6), 7.60 (d, *J* = 1.5 Hz, 1 H, 3-*H*3), 7.46 (d, *J* = 8.3 Hz, 2 H, 1-*H*Ar2), 7.44 – 7.38 (m, 5 H, 1-*H*2, 1-*H*Ar3, 2-Phe-*H*Ar3), 7.36 (d, *J* = 7.3 Hz, 2 H, 2-Phe-*H*Ar2), 7.30 (dd, *J* = 8.3, 1.7 Hz, 1 H, 3-*H*5), 7.25 (dd, *J* = 8.5, 1.7 Hz, 1 H, 1-*H*6), 6.63 (d, *J* = 8.3 Hz, 1 H, 1-*H*5), 5.30 (s. broad, 2 H, 1-NH_2), 5.11 (d, *J* = 2.1 Hz, 2 H, 1-*H*α), 4.84 – 4.76 (m, 2 H, 2-Phe-*H*α), 4.71 (quin, *J* = 5.9 Hz, 1 H, 3-*H*α), 4.04 (d, *J* = 5.1 Hz, 2 H, 4-Gly-*H*α), 3.16 – 3.04 (m, 2 H, 2-Phe-*H*β), 1.41 (dd, *J* = 8.5, 6.5 Hz, 6 H, 3-*H*β), 1.28 (s, 9 H, 1-HAr4-C($C\underline{H}_3$)$_3$); δ_C (125 MHz, DMSO-d_6) 171.2, 171.2, 166.4, 163.9, 156.2, 150.1, 144.1, 143.0, 141.6, 137.7, 134.2, 132.0, 131.5, 130.9, 127.4, 125.1, 121.7, 120.7, 119.5, 116.6, 112.3, 111.3, 111.1, 104.7, 71.9, 69.1, 55.6, 41.6, 36.4, 34.3, 31.1, 21.6; ν_{max}/cm^{-1} (solid state) = 2960, 2925, 2864, 1594, 1508, 1489, 1260, 1215, 1011; ESI-HRMS found *m/z* 759.2398 $[M+H]^+$, $C_{39}H_{44}BrN_4O_7$ requires 759.2388.

H_2N-[*O*-Bn-(3-HABA)]-2-Nal-[*O*-*i*Pr-(2-HABA)]-Gly-CO_2H 17

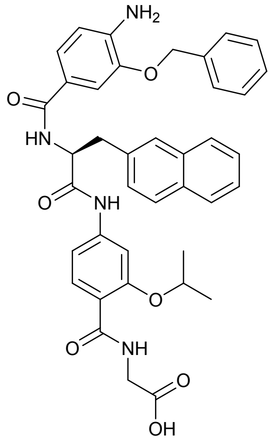

Brown solid; isolated yield: 18 mg, 27%; δ_H (500 MHz, MeOD) 7.93 (d, *J* = 8.5 Hz, 1 H, 3-*H*6), 7.81 – 7.68 (m, 4 H, 2-*H*Ar4, 2-*H*Ar5, 2-*H*Ar8, 2-*H*Ar7), 7.48 (d, *J* = 1.8 Hz, 1 H, 3-*H*3), 7.45 (dd, *J* = 8.5, 1.5 Hz, 1 H, 1-*H*2), 7.40 – 7.38 (m, 4 H, 1-Phe-*H*Ar2, 2-*H*Ar1, 2-*H*Ar6), 7.37 – 7.26 (m, 5 H, 2-*H*Ar3, 1-Phe-*H*Ar3, 1-Phe-*H*Ar4, 1-*H*6), 7.07 (dd, *J* = 8.7, 1.7 Hz, 1 H, 3-*H*5), 6.70 (d, *J* = 7.9 Hz, 1 H, 1-*H*5), 5.06 – 4.95 (m, 3 H, 1-*H*α, 2-Nal-*H*α), 4.65 (spt, *J* = 6.1, 1 H, 3-*H*α), 4.15 (s, 2 H, 4-Gly-*H*α), 3.44 (dd, *J* = 13.5, 8.0 Hz, 1 H, 2-Nal-*H*β), 3.34 (dd, *J* = 13.5, 7.0 Hz, 1 H, 2-Nal-*H*β′), 1.39 (d, *J* = 5.5 Hz, 3 H, 3-*H*β′), 1.38 (d, *J* = 5.5 Hz, 3 H, 3-*H*β); δ_C (125 MHz, MeOD) 172.9, 172.3, 170.2, 167.2, 158.3, 146.6, 144.1, 143.0, 138.4, 135.8, 134.9, 133.9, 133.1, 129.4, 129.0, 129.0, 128.9, 128.7, 128.6, 128.5, 128.4, 127.0, 126.6, 123.0, 122.8, 117.9, 114.4, 113.1, 112.3, 106.5, 73.6, 71.4, 57.5, 39.1, 22.1, 22.1; ν_{max}/cm^{-1} (solid state) = 3357, 2921, 2851, 1602, 1507, 1217, 697; ESI-HRMS found *m/z* 675.2827 $[M+H]^+$, $C_{39}H_{39}N_4O_7$ requires 675.2813.

H_2N-[*O*-Bn-(3-HABA)]-2-Nal-[*O*-*i*Pr-(2-HABA)]-Gly-CO_2H 18

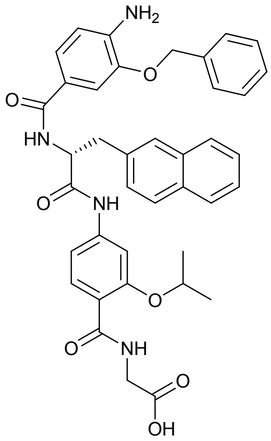

Brown solid; isolated yield: 25 mg, 37%; δ_H (500 MHz, MeOD) 7.91 (d, *J* = 8.8 Hz, 1 H, 3-*H*6), 7.77 – 7.66 (m, 4 H, 2-*H*Ar4, 2-*H*Ar5, 2-*H*Ar8, 2-*H*Ar7), 7.44 – 7.42 (m, 2 H, 3-*H*3, 1-*H*2), 7.38 – 7.34 (m, 2 H, 2-*H*Ar1, 2-*H*Ar6), 7.34 – 7.22 (m, 7 H, 1-Phe-*H*Ar2, 2-*H*Ar3, 1-Phe-*H*Ar3, 1-Phe-*H*Ar4, 1-*H*6), 7.08 (dd, *J* = 8.7, 1.8 Hz, 1 H, 3-*H*5), 6.67 (d, *J* = 7.9 Hz, 1 H, 1-*H*5), 5.10 (dd, *J* = 8.3, 6.8 Hz, 1 H, 2-Nal-*H*α), 4.88 (d, *J* = 7.9 Hz, 2 H, 1-*H*α), 4.53 (spt, *J* = 6.1, 1 H, 3-*H*α), 4.14 (s, 2 H, 4-Gly-*H*α), 3.44 (dd, *J* = 13.5, 8.0 Hz, 1 H, 2-Nal-*H*β), 3.34 (dd, *J* = 13.5, 7.0 Hz, 1 H, 2-Nal-*H*β′), 1.31 (d, *J* = 6.0 Hz, 6 H, 3-*H*β); δ_C (125 MHz, MeOD) 173.1, 172.6, 170.1, 167.1, 158.2, 146.6, 144.1, 143.0, 138.3, 135.8, 134.9, 133.8, 133.1, 129.4, 129.0, 129.0, 128.9, 128.7, 128.5, 128.5, 128.4, 127.0, 126.6, 122.9, 122.9, 117.8, 114.4, 113.0, 112.2, 106.4, 73.5, 71.3, 57.6, 39.1, 22.1, 22.0; ν_{max}/cm^{-1} (solid state) = 3376, 3054, 2979, 2929, 1603, 1443, 757, 697; ESI-HRMS found *m/z* 675.2820 $[M+H]^+$, $C_{39}H_{39}N_4O_7$ requires 675.2813.

H_2N-[*O*-CH_2-CH_2-CH_2-NH_2-(3-HABA)]-[*O*-*i*Bu-(3-HABA)]-[*O*-*s*Bu-(3-HABA)]-Gly-CO_2H 55

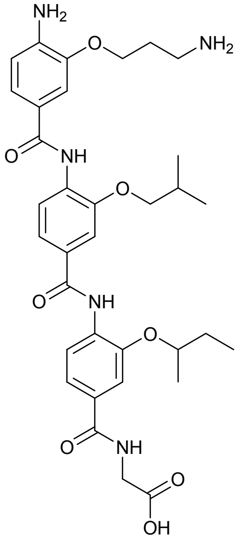

Brown solid; isolated yield: 63 mg, 97%; δ_H (500 MHz, MeOD) 8.34 (d, *J* = 8.1 Hz, 1 H, 2-*H*5), 8.30 (d, *J* = 8.3 Hz, 1 H, 3-*H*5), 7.55 (d, *J* = 1.7 Hz, 1 H, 2-*H*2), 7.49 – 7.46 (m, 3 H, 3-*H*2, 3-*H*6, 2-*H*6), 7.42 (d, *J* = 1.9 Hz, 1 H, 1-*H*2), 7.37 (dd, *J* = 8.1, 1.9 Hz, 2 H, 1-*H*6), 6.91 (d, *J* = 8.1 Hz, 1 H, 1-*H*5), 4.61 – 4.55 (m, 1 H, 2-*H*α), 4.21 (t, *J* = 5.8 Hz, 2 H, 1-*H*α), 4.10 (s, 2 H, 4-*H*α), 3.95 (d, *J* = 6.6 Hz, 2 H, 3-*H*α), 3.23 (t, *J* = 7.4 Hz, 2 H, 1-*H*γ), 2.26 – 2.16 (m, 4 H, 1-*H*β, 3-*H*β), 1.88 – 1.84 (m, 1 H, 2-*H*β), 1.84 – 1.74 (m, 1 H, 2-*H*β′), 1.41 (d, *J* = 6.0 Hz, 3 H, 2-CHα(C*H*$_3$)), 1.12 (d, *J* = 6.6 Hz, 6 H, 3-*H*γ), 1.06 (t, *J* = 7.5 Hz, 3 H, 2-*H*γ); δ_C (125 MHz, MeOD) 173.2, 169.6, 167.0, 166.5, 161.0, 160.7, 150.1, 148.9, 147.8, 133.4, 131.8, 131.1, 130.8, 125.7, 122.0, 121.3, 121.0, 120.6, 116.3, 113.1, 112.0, 111.3, 77.9, 76.2, 66.6, 42.3, 38.4, 30.2, 29.5, 28.3, 19.6, 19.5, 9.9; ν_{max}/cm^{-1} (solid state) = 3356, 2964, 2936, 1670, 1596, 1506, 1193, 1123, 1024, 721; ESI-HRMS found *m/z* 650.3198 $[M+H]^+$, $C_{34}H_{44}N_5O_8$ requires 650.3184.

H_2N-[*O*-*s*Bu-(3-HABA)]-[*O*-*s*Bu-(3-HABA)]-[*O*-*s*Bu-(3-HABA)]-Gly-CO_2H 56

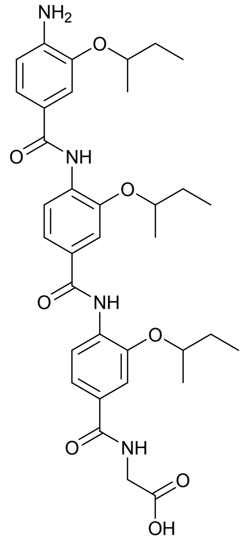

Beige solid; isolated yield: 48 mg, 74%; δ_H (500 MHz, $CDCl_3$) 8.85 (s, 1 H, 3-N*H*), 8.75 (s, 1 H, 2-N*H*), 8.68 (d, *J* = 8.3 Hz, 1 H, 2-*H*5), 8.59 (d, *J* = 8.1 Hz, 1 H, 3-*H*5), 7.58 (d, *J* = 1.0 Hz, 1 H, 2-*H*2), 7.53 (s, 1 H, 3-*H*2), 7.44 (d, *J* = 1.8 Hz, 1 H, 1-*H*2), 7. 42 – 7.37 (m, 2 H, 3-*H*6, 2-*H*6), 7.27 (dd, *J* = 8.1, 1.8 Hz, 2 H, 1-*H*6), 7.04 (s, broad, 1 H, 4-N*H*), 6.75 (d, *J* = 8.1 Hz, 1 H, 1-*H*5), 4.61 – 4.54 (m, 2 H, 3-*H*α, 2-*H*α), 4.49 – 4.43 (m, 1 H, 1-*H*α), 4.25 (s, 2 H, 4-*H*α), 1.89 – 1.65 (m, 6 H, 1-*H*β, 2-*H*β, 3-*H*β), 1.43 – 1.33 (m, 9 H, 1-CHα(C$\underline{H_3}$), 2-CHα(C$\underline{H_3}$), 3-CHα(C$\underline{H_3}$)), 1.08 – 0.97 (m, 9 H, 1-*H*γ, 2-*H*γ, 3-*H*γ); δ_C (125 MHz, $CDCl_3$) 167.7, 165.3, 164.8, 146.6, 146.6, 145.1, 141.5, 132.8, 132.3, 129.1, 128.2, 126.0, 124.6, 124.1, 119.9, 119.5, 119.0, 118.7, 113.7, 112.3, 111.9, 111.8, 76.7, 76.7, 76.0, 42.1, 29.3, 29.2, 29.2, 19.4, 19.3, 19.3, 9.8, 9.7, 9.6; v_{max}/cm^{-1} (solid state) = 3441, 3310, 3181, 2967, 2930, 1750, 1595, 1505, 1323, 1255, 1033, 746; ESI-HRMS found *m/z* 649.3245 $[M+H]^+$, $C_{35}H_{45}N_4O_8$ requires 649.3232.

H_2N-[*O*-CH_2-CH_2-CH_2-NH_2-(3-HABA)]-[*O*-*s*Bu-(3-HABA)]-[*O*-*i*Bu-(3-HABA)]-Gly-CO_2H 57

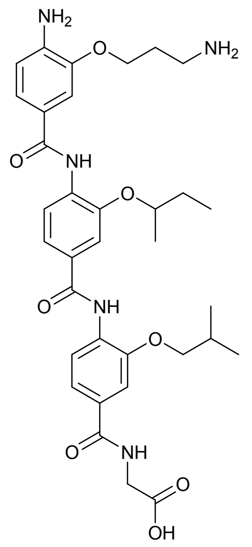

Brown solid; isolated yield: 61 mg, 94%; δ_H (500 MHz, MeOD) 8.37 (d, *J* = 8.3 Hz, 1 H, 2-*H*5), 8.26 (d, *J* = 8.1 Hz, 1 H, 3-*H*5), 7.57 (d, *J* = 1.9 Hz, 1 H, 2-*H*2), 7.53 (s, 1 H, 3-*H*2), 7.52 – 7.47 (m, 2 H, 3-*H*6, 2-*H*6), 7.44 (d, *J* = 1.9 Hz, 1 H, 1-*H*2), 7.35 (dd, *J* = 8.1, 1.9 Hz, 2 H, 1-*H*6), 6.93 (d, *J* = 8.1 Hz, 1 H, 1-*H*5), 4.64 – 4.56 (m, 1 H, 2-*H*α), 4.22 (t, *J* = 5.8 Hz, 2 H, 1-*H*α), 4.10 (s, 2 H, 4-*H*α), 3.94 (d, *J* = 6.6 Hz, 2 H, 3-*H*α), 3.23 (t, *J* = 7.3 Hz, 2 H, 1-*H*γ), 2.25 – 2.17 (m, 4 H, 1-*H*β, 3-*H*β), 1.88 – 1.84 (m, 1 H, 2-*H*β), 1.84 – 1.74 (m, 1 H, 2-*H*β′), 1.41 (d, *J* = 6.0 Hz, 3 H, 2-CHα(C$\underline{H_3}$)), 1.11 (d, *J* = 6.8 Hz, 6 H, 3-*H*γ), 1.06 (t, *J* = 7.5 Hz, 3 H, 2-*H*γ); δ_C (125 MHz, MeOD) 173.2, 169.6, 167.0, 166.8, 161.0, 160.7, 150.3, 148.9, 147.9, 133.4, 131.8, 131.1, 131.0, 125.8, 121.8, 121.2, 121.0, 120.6, 116.4, 113.0, 112.0, 111.6, 78.1, 76.2, 66.6, 42.3, 38.4, 30.2, 29.5, 28.2, 19.6, 19.5, 9.9; v_{max}/cm^{-1} (solid state) = 3421, 2964, 1595, 1507, 1127, 1026, 747; ESI-HRMS found *m/z* 650.3197 $[M+H]^+$, $C_{34}H_{44}N_5O_8$ requires 650.3184.

H_2N-[*O*-*s*Bu-(3-HABA)]-[*O*-*i*Bu-(3-HABA)]-[*O*-CH_2-CH_2-NH_2-(3-HABA)]-Gly-CO_2H 58

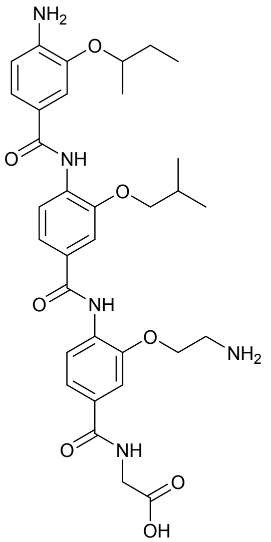

Beige solid; isolated yield: 8 mg, 13%; δ_H (500 MHz, DMSO-d_6) 8.99 (s, 1 H, 2-N*H*), 8.44 (s, broad, 1 H, 4-N*H*), 8.25 (d, *J* = 8.5 Hz, 1 H, 2-*H*5), 8.11 (d, *J* = 8.3 Hz, 1 H, 3-*H*5), 7.66 – 7.61 (m, 2 H, 2-*H*2, 2-*H*6), 7.59 (s, 1 H, 3-*H*2), 7.53 (d, *J* = 8.1 Hz, 1 H, 3-*H*6), 7.39 – 7.29 (m, 2 H, 1-*H*2, 1-*H*6), 6.72 (d, *J* = 7.9 Hz, 1 H, 1-*H*5), 5.42 (s, broad, 1 H, 1-N*H*), 4.42 – 4.37 (m, 1 H, 1-*H*α), 4.30 (s, 2 H, 4-*H*α), 4.21 (t, *J* = 5.8 Hz, 2 H, 3-*H*α), 3.97 (d, *J* = 6.0 Hz, 2 H, 2-*H*α), 3.74 (s, 2 H, 3-N*H*), 3.21 (s, 2 H, 3-*H*β), 2.21 – 2.12 (m, 1 H, 2-*H*β), 1.77 – 1.70 (m, 1 H, 1-*H*β), 1.68 – 1.60 (m, 1 H, 1-*H*β′), 1.27 (d, *J* = 6.0 Hz, 3 H, 1-CHα(C$\underline{H}_3$)), 1.06 (d, *J* = 6.6 Hz, 6 H, 2-*H*γ), 0.96 (t, *J* = 7.5 Hz, 3 H, 1-*H*γ); δ_C (125 MHz, DMSO-d_6) 172.0, 165.0, 164.8, 164.5, 148.5, 148.4, 143.4, 143.3, 131.2, 130.6, 130.0, 129.6, 121.9, 121.3, 120.9, 120.4, 120.3, 120.1, 112.7, 112.2, 111.1, 110.9, 75.3, 74.6, 66.9, 42.8, 35.6, 28.6, 27.8, 19.1, 19.1, 9.6; ν_{max}/cm^{-1} (solid state) = 3351, 2963, 2931, 1575, 1508, 1382, 1252, 1025, 753; ESI-HRMS found *m/z* 636.3043 $[M+H]^+$, $C_{33}H_{42}N_5O_8$ requires 636.3028.

REFERENCES

1 Azzarito, V., Miles, J. A., Fisher, J., Edwards, T. A., Warriner, S. & Wilson, A. Stereocontrolled Protein Surface Recognition Using Chiral Oligoamide Proteomimetic Foldamers. *Chem. Sci.* **6**, 2434-2443, (2015).

2 Azzarito, V. *Versatile Oligoamide α-helix Mimetic Scaffolds* PhD thesis, University of Leeds, (2014). http://etheses.whiterose.ac.uk/6888/

3 Azzarito, V., Rowell, P., Barnard, A., Edwards, T. A., Macdonald, A., Warriner, S. L. & Wilson, A. J. Probing Protein Surfaces: QSAR Analysis with Helix Mimetics. *ChemBioChem* **2016**, 768-773, (2016).

4 Plante, J. P., Burnley, T., Malkova, B., Webb, M. E., Warriner, S. L., Edwards, T. A. & Wilson, A. J. Oligobenzamide proteomimetic inhibitors of the p53-hDM2 protein-protein interaction. *Chem. Commun.*, 5091-5093, (2009).

5 Azzarito, V., Prabhakaran, P., Bartlett, A. I., Murphy, N. S., Hardie, M. J., Kilner, C. A., Edwards, T. A., Warriner, S. L. & Wilson, A. J. 2-*O*-Alkylated Para-Benzamide α–Helix Mimetics: The Role of Scaffold Curvature. *Org. Biomol. Chem.* **10**, 6469-6472, (2012).

6 Burslem, G. M., Kyle, H. F., Breeze, A. L., Edwards, T. A., Nelson, A., Warriner, S. L. & Wilson, A. J. Small-Molecule Proteomimetic Inhibitors of the HIF-1α–p300 Protein–Protein Interaction. *ChemBioChem* **15**, 1083-1087, (2014).

7 Barnard, A., Long, K., Martin, H. L., Miles, J. A., Edwards, T. A., Tomlinson, D. C., Macdonald, A. & Wilson, A. J. Selective and Potent Proteomimetic Inhibitors of Intracellular Protein–Protein Interactions. *Angew. Chem. Int. Ed.* **54**, 2960-2965, (2015).

8 Long, K., Edwards, T. & Wilson, A. Microwave assisted solid phase synthesis of highly functionalized N-alkylated oligobenzamide α-helix mimetics. *Bioorg. Med. Chem.* **21**, 4034-4040, (2013).

9 Tripathy, D. R., Dinda, A. K. & Dasgupta, S. A simple assay for the ribonuclease activity of ribonucleases in the presence of ethidium bromide. *Anal. Biochem.* **437**, 126-129, (2013).

10 Kuzmič, P. in *Methods Enzymol.* Vol. 467 247-280 (Academic Press, 2009).

11 Friesner, R. A., Murphy, R. B., Repasky, M. P., Frye, L. L., Greenwood, J. R., Halgren, T. A., Sanschagrin, P. C. & Mainz, D. T. Extra Precision Glide: Docking and Scoring Incorporating a Model of Hydrophobic Enclosure for Protein−Ligand Complexes. *J. Med. Chem.* **49**, 6177-6196, (2006).

12 Halgren, T. A., Murphy, R. B., Friesner, R. A., Beard, H. S., Frye, L. L., Pollard, W. T. & Banks, J. L. Glide: A New Approach for Rapid, Accurate Docking and Scoring. 2. Enrichment Factors in Database Screening. *J. Med. Chem.* **47**, 1750-1759, (2004).

13 Friesner, R. A., Banks, J. L., Murphy, R. B., Halgren, T. A., Klicic, J. J., Mainz, D. T., Repasky, M. P., Knoll, E. H., Shelley, M., Perry, J. K., Shaw, D. E., Francis, P. & Shenkin, P. S. Glide: A New Approach for Rapid, Accurate Docking and Scoring. 1. Method and Assessment of Docking Accuracy. *J. Med. Chem.* **47**, 1739-1749, (2004).

14 Murphy, N., Prabhakaran, P., Azzarito, V., Plante, J., Hardie, M., Kilner, C., Warriner, S. & Wilson, A. Solid-phase methodology for synthesis of O-alkylated aromatic oligoamide inhibitors of α-helix-mediated protein-protein interactions. *Chem. Eur. J.* **19**, 5546-5550, (2013).

[15] Plante, J., Campbell, F., Malkova, B., Kilner, C., Warriner, S. L. & Wilson, A. J. Synthesis of Functionalised Aromatic Oligamide Rods. *Org. Biomol. Chem.* **6**, 138-146, (2008).
[16] Burslem, G. M., Kyle, H. F., Prabhakaran, P., Breeze, A. L., Edwards, T. A., Warriner, S. L., Nelson, A. & Wilson, A. J. Synthesis of highly functionalized oligobenzamide proteomimetic foldamers by late stage introduction of sensitive groups. *Org. Biomol. Chem.* **14**, 3782-3786, (2016).
[17] Murphy, N. S., Prabhakaran, P., Azzarito, V., Plante, J. P., Hardie, M. J., Kilner, C. A., Warriner, S. L. & Wilson, A. J. Solid-Phase Methodology for Synthesis of O-Alkylated Aromatic Oligoamide Inhibitors of α-Helix-Mediated Protein–Protein Interactions. *Chem. Eur. J.* **19**, 5546-5550, (2013).
